# BBLN triggers CAMK2D pathology in mice under cardiac pressure overload and potentially in unrepaired hearts with tetralogy of Fallot

**DOI:** 10.1038/s44161-023-00351-6

**Published:** 2023-10-26

**Authors:** Joshua Abd Alla, Andreas Langer, Stefan Wolf, Xuebin Fu, Mohamed Abdelfattah Rageh, Ursula Quitterer

**Affiliations:** 1 Molecular Pharmacology, Department of Chemistry and Applied Biosciences, ETH Zurich, Zurich, Switzerland; 2 Department of Pediatrics, Northwestern University Feinberg School of Medicine, Chicago, IL USA; 3 Department of Cardiovascular–Thoracic Surgery, Ann & Robert H. Lurie Children’s Hospital, Chicago, IL USA; 4 Ain Shams University Hospitals, Cairo, Egypt; 5 Department of Pharmacology and Toxicology, University of Zurich, Zurich, Switzerland; 6 Present Address: Dalhousie University of Canada, Halifax, Nova Scotia Canada

**Keywords:** Molecular medicine, Heart failure, Cardiovascular diseases, Disease model

## Abstract

Tetralogy of Fallot (TOF) is one of the most prevalent congenital heart defects, with adverse cardiac remodeling and long-term cardiac complications. Here, searching for pathomechanisms, we find upregulated bublin coiled-coil protein (BBLN) in heart specimens of TOF patients with cyanosis, which positively correlates with cardiac remodeling pathways. Human BBLN, a protein with largely unknown function, promoted heart failure features, with increased mortality when overexpressed in mice, in a protein dosage-dependent manner. BBLN enhanced cardiac inflammation, fibrosis and necroptosis by calcium/calmodulin-dependent protein kinase II delta (CAMK2D) activation, whereas a BBLN mutant with impaired CAMK2D binding was inert. Downregulation of CAMK2D by an interfering RNA retarded BBLN-induced symptoms of heart failure. Endogenous BBLN was induced by hypoxia as a major TOF feature in human patients and by chronic pressure overload in mice, and its downregulation decreased CAMK2D hyperactivity, necroptosis and cardiovascular dysfunction. Thus, BBLN promotes CAMK2D-induced pathways to pathological cardiac remodeling, which are triggered by hypoxia in TOF.

## Abstract (web summary)

Abd Alla et al. identify bublin coiled-coil protein (BBLN) as a factor upregulated in unrepaired hearts of patients with tetralogy of Fallot, and show that this protein binds to and controls the function of calcium/calmodulin-dependent protein kinase II delta (CAMK2D). Overexpression of BBLN in mice induced pathological cardiac remodeling, which was precluded when the interaction of BBLN with CAMK2D was prevented or upon CAMK2D downregulation.

## Main

It is estimated that over 7,000 rare diseases affect about 300 million people worldwide^1^. By definition, a rare disease affects fewer than 5 out of 10,000 people, and so far, the causes of most rare diseases are not understood^1^. On the basis of many successful examples, the elucidation of rare diseases is not only relevant for the few directly affected patients but also supports precision medicine approaches for patients with similar common pathologies^2,3^. With a worldwide prevalence of 3–4 cases per 10,000 live births, tetralogy of Fallot (TOF) is also a rare disease. In addition, TOF is among the most prevalent congenital heart defects and the most frequent congenital cyanotic heart defect^4–6^. It is characterized by four major cardiac abnormalities, which consist of a ventricular septal defect, an overriding aortic root, an infundibular stenosis of the pulmonary artery and a right ventricular hypertrophy^4–6^. Heart defects of TOF cause impaired oxygen delivery to the infant and, depending on severity, culminate in cyanosis^4–6^. Treatment of TOF relies on surgery to correct cardiac defects^7^. Despite greatly improved surgical procedures in infancy and early childhood, long-term complications of corrected TOF remain a major problem^7–10^. Besides coronary artery disease, long-term cardiac complications of TOF include cardiac hypertrophy and cardiac remodeling, which predispose to an increased risk of cardiac death due to arrhythmias and heart failure^7–10^. Refinement of surgical correction methods for TOF improved the survival in the short term but the elevated cardiovascular long-term risk of TOF patients did not change over the past decades^7–10^. Besides surgery, specific treatment options for TOF patients are not available, mostly because the pathomechanisms underlying the cardiac remodeling process triggered by TOF are unknown^7–10^.

Previous studies have found that calcium/calmodulin-dependent protein kinase II delta (CAMK2D) levels were upregulated in obstructed right ventricular specimens of TOF infants compared with those with a ventricular septal defect, and CAMK2D is a major pathological factor of cardiac remodeling and heart failure^10–13^.

In this Article, to identify pathomechanisms triggered by TOF, we searched for CAMK2D-interacting proteins in cardiac specimens of TOF patients undergoing surgical correction of the right ventricular outflow tract (RVOT) obstruction.

We identified bublin coiled-coil protein (BBLN) as a CAMK2D-interacting protein that is upregulated in TOF cyanotic patients compared with noncyanotic ones. Given the largely unknown function of BBLN, we generated two transgenic mouse models overexpressing the gene 25 or 50 times more than wild-type mice, and found that *BBLN* overexpression induced cardiac dysfunction in mice in a dose-dependent way. Downregulation of CAMK2D by an interfering RNA retarded BBLN-induced symptoms of heart failure. We found that endogenous BBLN was induced by chronic pressure overload in mice, and it is probably induced by hypoxia in cyanotic TOF patients. *BBLN* downregulation in mice exposed to pressure overload, decreased CAMK2D-induced inflammation, fibrotic remodeling and necroptosis. Overall, our study suggests BBLN as a regulator of CAMK2D activity and a potential target to prevent adverse cardiac remodeling in different conditions, including TOF.

## BBLN is upregulated in TOF patients with cyanosis

**Fig. 1:**
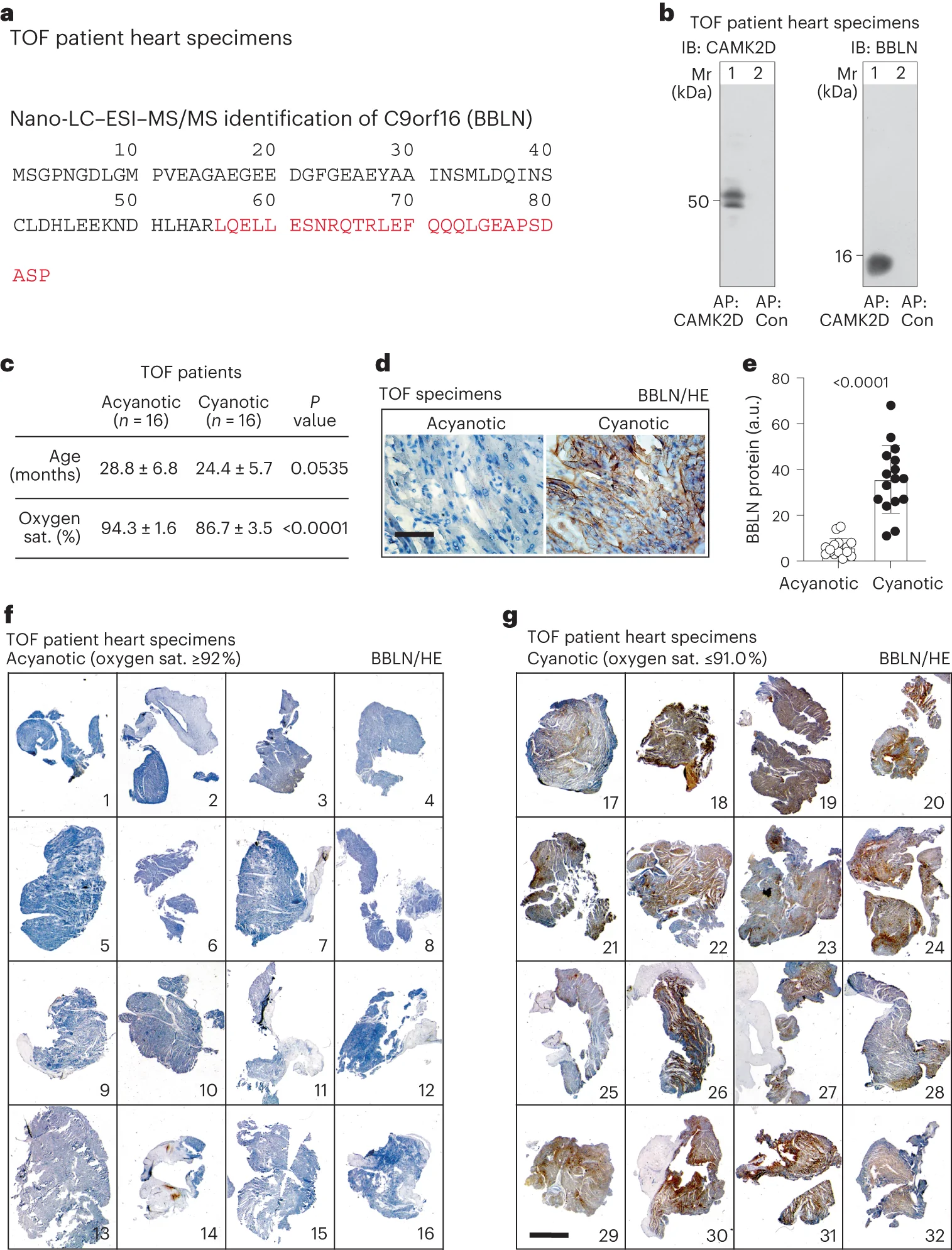
Identification of the CAMK2D-interacting protein BBLN in cardiac specimens of TOF patients. **a**, Identification of BBLN in TOF patient heart specimens by nano-LC–ESI–MS/MS analysis of CAMK2D-co-enriched proteins in the 8–14 kDa range. **b**, AP of CAMK2D from cardiac specimens of TOF patients (AP: CAMK2D) followed by IB detection of enriched CAMK2D (IB: CAMK2D) and co-enriched BBLN (IB: BBLN). The control immunoaffinity matrix (AP: con) did not enrich CAMK2D nor BBLN. The experiment was repeated three times with similar results. **c**, Characteristics of TOF patients who were included in the study for the immunohistological determination of cardiac BBLN. Age between acyanotic and cyanotic TOF patients was comparable while oxygen saturation (sat.) was significantly different. **d**, Immunohistological detection of BBLN on cardiac specimens of TOF patients without (left) and with cyanosis (right). Scale bar, 40 μm. Immunohistological detection of BBLN was performed on specimens of 16 acyanotic TOF patients and 16 cyanotic TOF patients (cf. **f** and **g**). **e**–**g**, Immunohistological determination of BBLN on cardiac specimens of 16 acyanotic TOF patients^1–16^ and 16 cyanotic TOF patients^17–32^. Counterstaining was performed with hematoxylin (HE). Quantitative BBLN data (**e**) and immunohistological images of cardiac specimens from acyanotic TOF patients (**f**) and cyanotic TOF patients (**g**) are shown. Scale bar, 2 mm. Data are mean ± s.d. (*n* = 16 patients per group; unpaired, two-tailed *t*-test; d.f. 30; **c**, *t* = 2.010 and 7.820, *P* = 0.05354 and 9.99913 × 10^−9^; **e**, *t* = 7.811 and *P* = 1.02375 × 10^−8^). Source data

Immunoaffinity enrichment (AP) of CAMK2D from TOF patient heart specimens, and nanoliquid chromatography–electrospray ionization–tandem mass spectrometry (nano-LC–ESI–MS/MS) identification of co-enriched proteins in the low molecular weight 8–14 kDa range, revealed BBLN as a previously unrecognized CAMK2D-interacting protein (Fig. 1a). Immunoblot (IB) detection confirmed the identity of the CAMK2D-interacting protein as BBLN (Fig. 1b). Immunohistological analyses found that BBLN protein contents were increased sixfold (range 1.9- to 11.45-fold) on cardiac biopsy specimens of TOF patients with cyanosis compared with those of acyanotic TOF cases (Fig. 1c–g).

**Extended Data Fig. 1:**
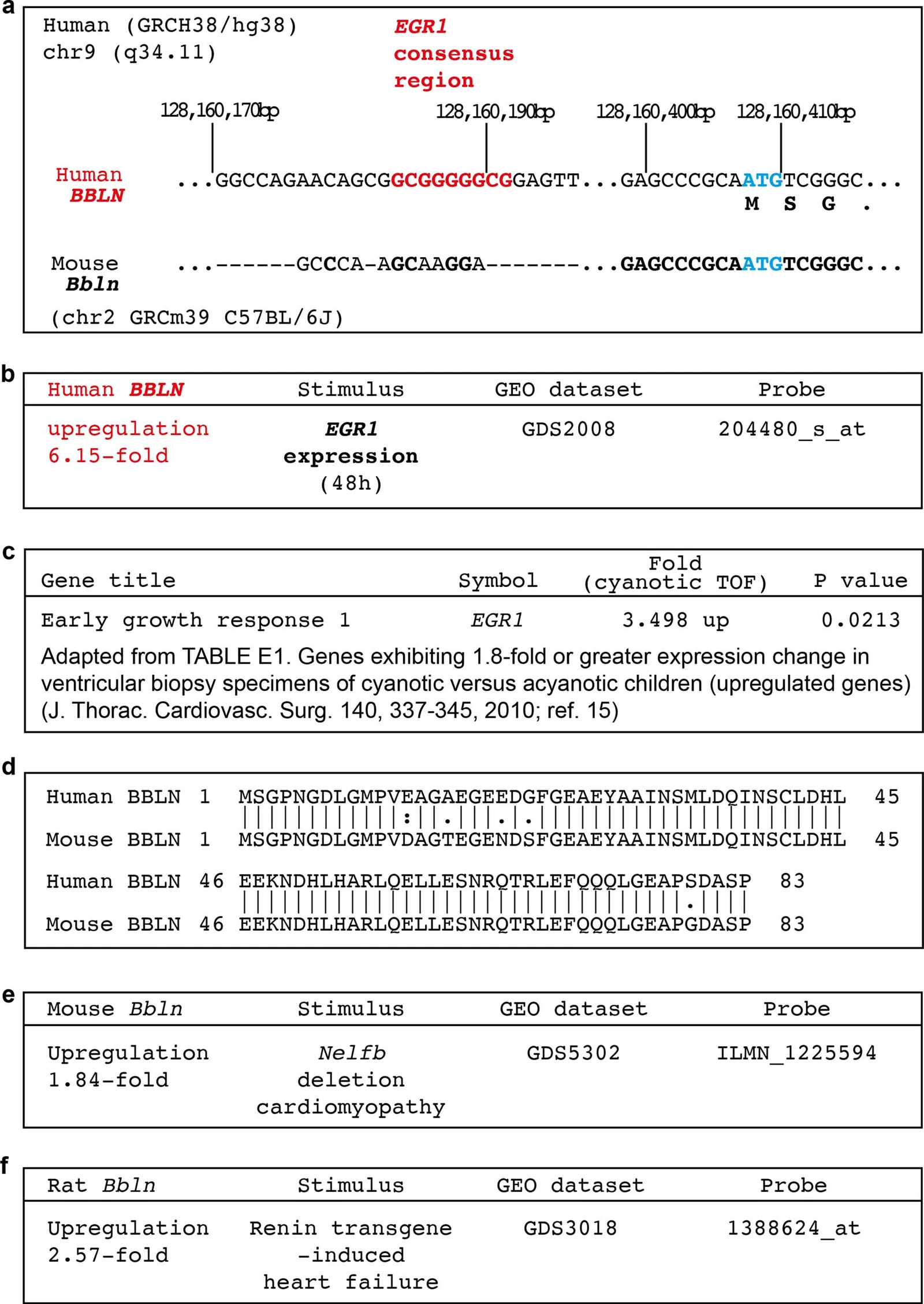
The human *BBLN* gene is an *EGR1*-responsive gene, and the rodent *Bbln* is up-regulated in mouse and rat heart failure models. **a**, Identification of the *EGR1* (Early growth response 1) consensus region (marked in red) in the human *BBLN* gene. The *Egr1* consensus region is absent in the mouse *Bbln* gene. Nucleotides which are identical in the human *BBLN* and mouse *Bbln* genomic sequences are highlighted in bold in the murine sequence. The initiator ATG is marked in blue. **b**, The GEO DataSet GDS2008 documents that the human *BBLN* transcript is strongly up-regulated by *EGR1* expression. **c**, The *EGR1* transcript was found up-regulated in infants with TOF and cyanosis. Data were taken from TABLE E1 of ref. ^15^. The p-value was determined with an unpaired, two-tailed, equal variance Student’s t-test, and Benjamini and Hochberg multiple testing correction for false discovery rate, FDR (ref. ^15^). **d**, Sequence alignment of human and mouse BBLN amino acid sequences. Sequence alignment shows 94.0 % identity (78/83) and 95.2 % similarity (79/83) between human and mouse BBLN amino acid sequences. Pairwise sequence alignment was performed with EMBOSS Needle (Matrix: EBLOSUM62; Gap penalty: 10.0; Extend penalty: 0.5). Residues with conservation between groups of strongly similar properties (:), and residues with conservation between groups of weakly similar properties (.) are marked. **e**,**f**, GEO DataSet GDS5302 in the NCBI GEO database shows that mouse *Bbln* expression is up-regulated in murine hearts with *NelfB* deletion cardiomyopathy (**e**), and GDS3018 shows that the rat *Bbln* transcript is up-regulated in hearts with renin transgene-induced heart failure (**f**). Source data

The human *BBLN* gene is an early growth response 1 (*EGR1*)-responsive gene with an *EGR1* consensus site in the promoter region, and according to Gene Expression Omnibus (GEO) dataset GDS2008, *BBLN* is induced by *EGR1* expression (Extended Data Fig. 1a,b). The upregulation of the human BBLN protein in cyanotic TOF patient hearts could thus be a direct consequence of the low oxygen condition because *EGR1* is upregulated under hypoxic conditions^14^. In agreement with this notion, ventricular specimens from cyanotic TOF patients displayed significantly upregulated *EGR1* transcript levels compared with acyanotic TOF cases (data from ref. ^15^ are shown in Extended Data Fig. 1c). Although the human and murine BBLN amino acid sequences have 94% identity, the murine *Bbln* gene lacks the *Egr1* consensus site (Extended Data Fig. 1a,d) and could therefore be upregulated by other modalities in different heart failure models (Extended Data Fig. 1e,f).

## Tg-*BBLN* mice developed dilative cardiac hypertrophy

**Fig. 2:**
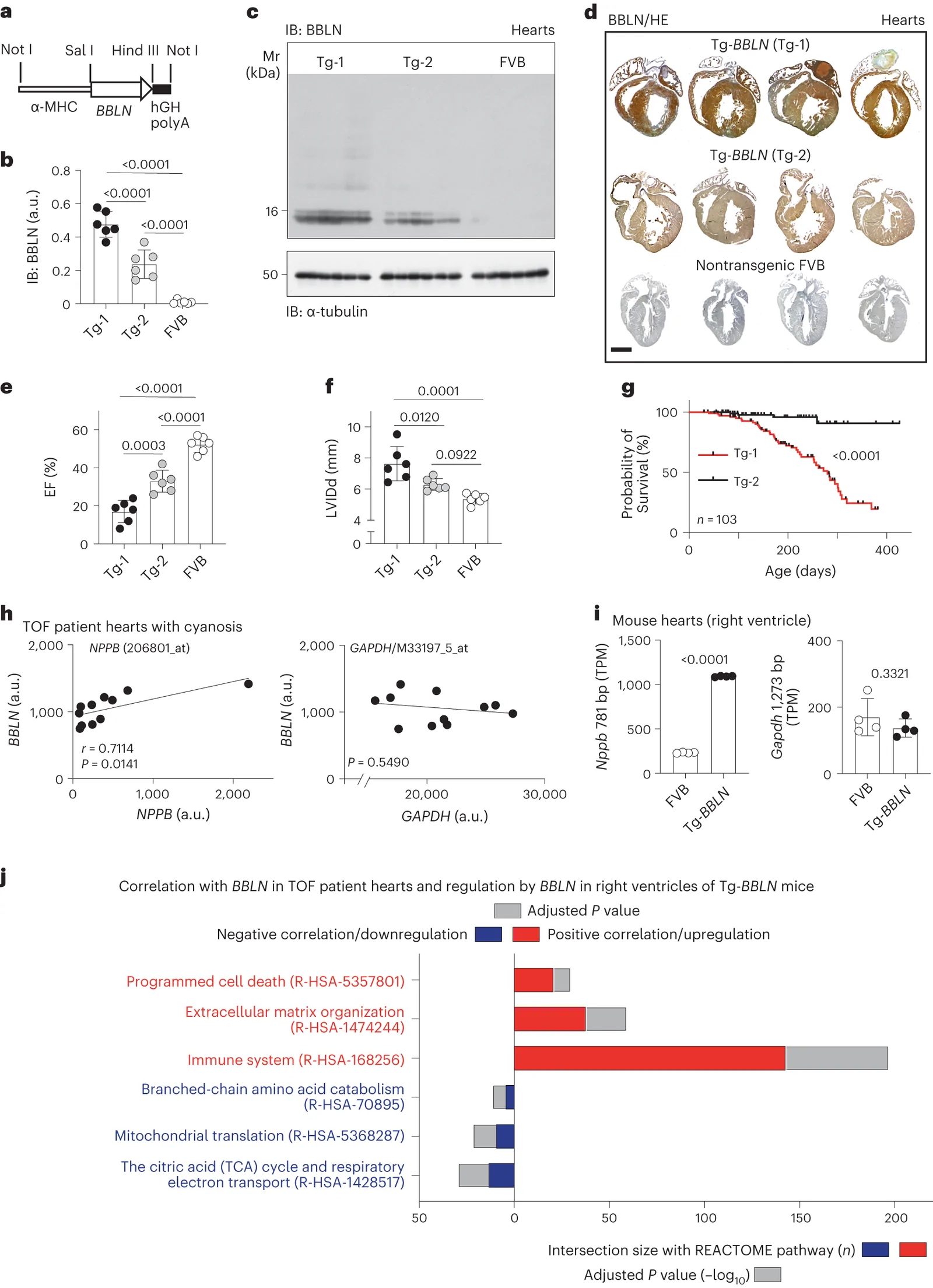
Tg-*BBLN* mice developed dilative cardiac hypertrophy with features of TOF patient hearts. **a**, A scheme of the transgenic vector (Not I: NotI restriction endonuclease from *Nocardia otidiscaviarum*; Sal I: SalI restriction endonuclease from *Streptomyces albus G;* Hind III: HindIII restriction endonuclease from *Haemophilus influenzae Rd*). **b**,**c**, Quantitative IB data of cardiac BBLN (IB: BBLN) of two different Tg-*BBLN* mouse lines (Tg-1 and Tg-2) compared with nontransgenic FVB mice (**b**). Representative IBs are shown in **c**. Data are mean ± s.d., *n* = 6 male mice per group, age: 3–4 months (one-way ANOVA and Tukey’s test; *F*(2,15) = 73.28 and *P* = 0.00004711, 1.106 × 10^−8^ and 0.00008219). **d**, Immunohistological analysis of BBLN in Tg-*BBLN* hearts compared with FVB controls (*n* = 4 male mice per group, age 3–4 months. Scale bar, 2 mm; counterstaining with HE). **e**,**f**, The left ventricular EF (**e**) and the left ventricular internal diameter in diastole (LVIDd) (**f**) were determined by echocardiography. Data are mean ± s.d. (*n* = 6 male mice per group, age 3–4 months; one-way ANOVA and Tukey’s test; *F*(2,15) = 65.62 (**e**) and 15.83 (**f**); *P* = 0.0003107, 2.384 × 10^−8^ and 0.00004306 (**e**); *P* = 0.01204, 0.0001435 and 0.09218 (**f**). **g**, Decreased lifespan of male Tg-*BBLN* mice (*n* = 103 mice per group; Kaplan–Meier survival analysis with log-rank (Mantel–Cox) test); *F*(2,15) = 28.20 and *P* < 0.0001). **h**, Cardiac transcript levels of *BBLN*, *NPPB* and *GAPDH* in TOF patients with cyanosis were determined by microarray. Linear regression analysis was performed, and the Pearson correlation coefficient (*r*) and *P* values (two tailed) were determined (*n* = 11 TOF patients with cyanosis; *P* = 0.0141 and 0.5490). **i**, NGS determination of transcript levels of *Bbln* and *Gapdh* in right ventricular cardiac tissue of Tg-*BBLN* mice compared with FVB mice. Data are mean ± s.d. (*n* = 4 male mice per group, age 3–4 months; unpaired, two-tailed *t*-test; d.f. 6, *t* = 193.7 and 1.055; *P* = 1.27855 × 10^−12^ *Nppb* and *P* = 0.3321 *Gapdh*). **j**, Overrepresentation analysis of transcripts having positive correlation with *BBLN* in RVOT specimens of TOF patients with cyanosis and showing upregulation in right ventricles of Tg-*BBLN* mice (red), or having negative correlation with *BBLN* in TOF patient hearts and downregulation in right ventricles of Tg-*BBLN* mice (blue). Red indicates a positive correlation with *BBLN* in TOF patients and upregulation in Tg-*BBLN* mice, whereas blue indicates a negative correlation with *BBLN* in TOF patients and downregulation in Tg-*BBLN* mice. The stable Reactome pathway identifiers (R-HSA-xxx) of major up-regulated and down-regulated pathways are indicated. Adjusted *P* values (−log_10_) are shown in gray (Fisher’s one-tailed test, multiple testing correction with G:SCS algorithm of g:GOSt; *P* = 7.57 × 10^−10^, 3.23 × 10^−22^, 5.67 × 10^−55^, 1.19 × 10^−7^, 6.22 × 10^−13^ and 9.84 × 10^−17^). Source data

As the in vivo function of the human BBLN protein is largely unknown, we investigated the impact of an increased cardiac BBLN content by generation of *BBLN*-transgenic (Tg-*BBLN*) mice. Although TOF originates in the right ventricle, the BBLN-inducing hypoxia in cyanotic TOF patients affects the whole heart, and the deoxygenated blood flows through the right and left ventricle and the coronary blood vessels. Therefore, *BBLN* was expressed under control of the alpha-myosin heavy chain (MHC) promoter, which induces myocardium-specific *BBLN* expression in the whole heart (Fig. 2a). Two different Tg-*BBLN* mouse lines were generated (Tg-1 and Tg-2) with 50.4 ± 8.2-fold and 25.1 ± 9.0-fold increased cardiac BBLN protein contents, respectively, compared with those of nontransgenic Friend leukemia virus B susceptible (FVB) control mice (Fig. 2b,c), which feature the increased BBLN contents of TOF patient hearts (cf. Fig. 1e). Immunohistological analysis of hearts from 3–4-month-old Tg-*BBLN* mice confirmed increased cardiac BBLN protein contents and showed cardiomegaly with enlargement of right and left ventricles (Fig. 2d). Frequently, Tg-*BBLN* mice (Tg-1) developed atrial myxomas (Fig. 2d), which are also reported in some TOF cases^16^ and could arise from multipotent cardiac stem cells during unsuccessful heart repair^17^. However, increased BBLN contents in cardiac specimens of TOF patients were a consequence and not a cause of TOF defects because Tg-*BBLN* mice with heart failure had no septal defect (Fig. 2d). Concomitant with cardiac enlargement, the increased cardiac BBLN protein content triggered cardiac dysfunction (Fig. 2e). The cardiac phenotype of Tg-*BBLN* mice was BBLN protein dosage dependent. Tg-*BBLN* mice with 50.4 ± 8.2-fold increased cardiac BBLN protein levels (Tg-1), had an early heart failure phenotype with a strongly reduced left ventricular ejection fraction (EF) of 18.0 ± 6.0% and an increased mortality starting at an age of 3 months (Fig. 2e,g).

**Extended Data Fig. 2:**
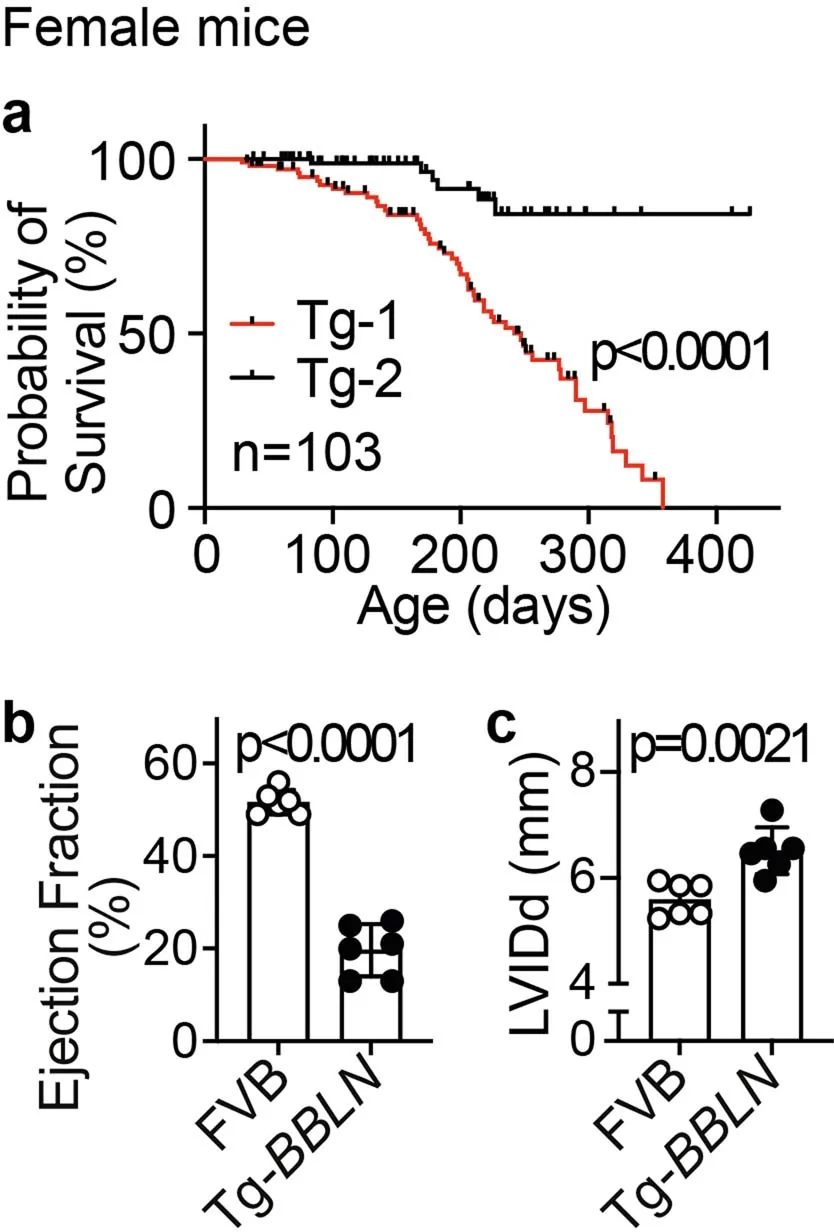
Female Tg-*BBLN* mice developed a heart failure phenotype with an increased mortality. **a**, Decreased lifespan of female Tg-*BBLN* mice (Tg-1 and Tg-2), was detected by Kaplan-Meier survival analysis with log-rank (Mantel-Cox) test (n = 103 mice per group; p < 0.0001). **b**, Symptoms of heart failure with a significantly decreased left ventricular ejection fraction (EF) were detected by echocardiography in female Tg-*BBLN* mice (Tg-1) at an age of 3–4 months. **c**, Female Tg-*BBLN* mice (Tg-1) also developed cardiac hypertrophy as documented by an increased left ventricular internal diameter in diastole (LVIDd). Data (**b**,**c**) are mean ± s.d. (n = 6 female mice per group; age: 3–4 months; unpaired, two-tailed t-test; df=10; **b**, t = 12.56; p = 1.89718E-07; **c**, t = 4.113; p = 0.0021). Controls were age-matched, non-transgenic, female FVB mice. Source data

At the same age, Tg-*BBLN* mice with 25.1 ± 9.0-fold increased cardiac *BBLN* levels (Tg-2) had a significantly decreased left ventricular EF of 33.0 ± 5.8% compared with 52.3 ± 4.2% of age-matched, nontransgenic FVB mice (Fig. 2e). The 1-year survival rate was 19.6% of male Tg-1 mice and 90.8% of male Tg-2 mice (Fig. 2g). The heart failure phenotype with an increased mortality was also observed in female Tg-*BBLN* mice (Extended Data Fig. 2a,b). Echocardiographic analyses confirmed the immunohistological assessment and showed that the heart failure phenotype of Tg-*BBLN* mice was accompanied by enlargement of the left cardiac ventricles (Fig. 2f and Extended Data Fig. 2c).

## Tg-*BBLN* mice reproduce features of TOF patients

**Fig. 3:**
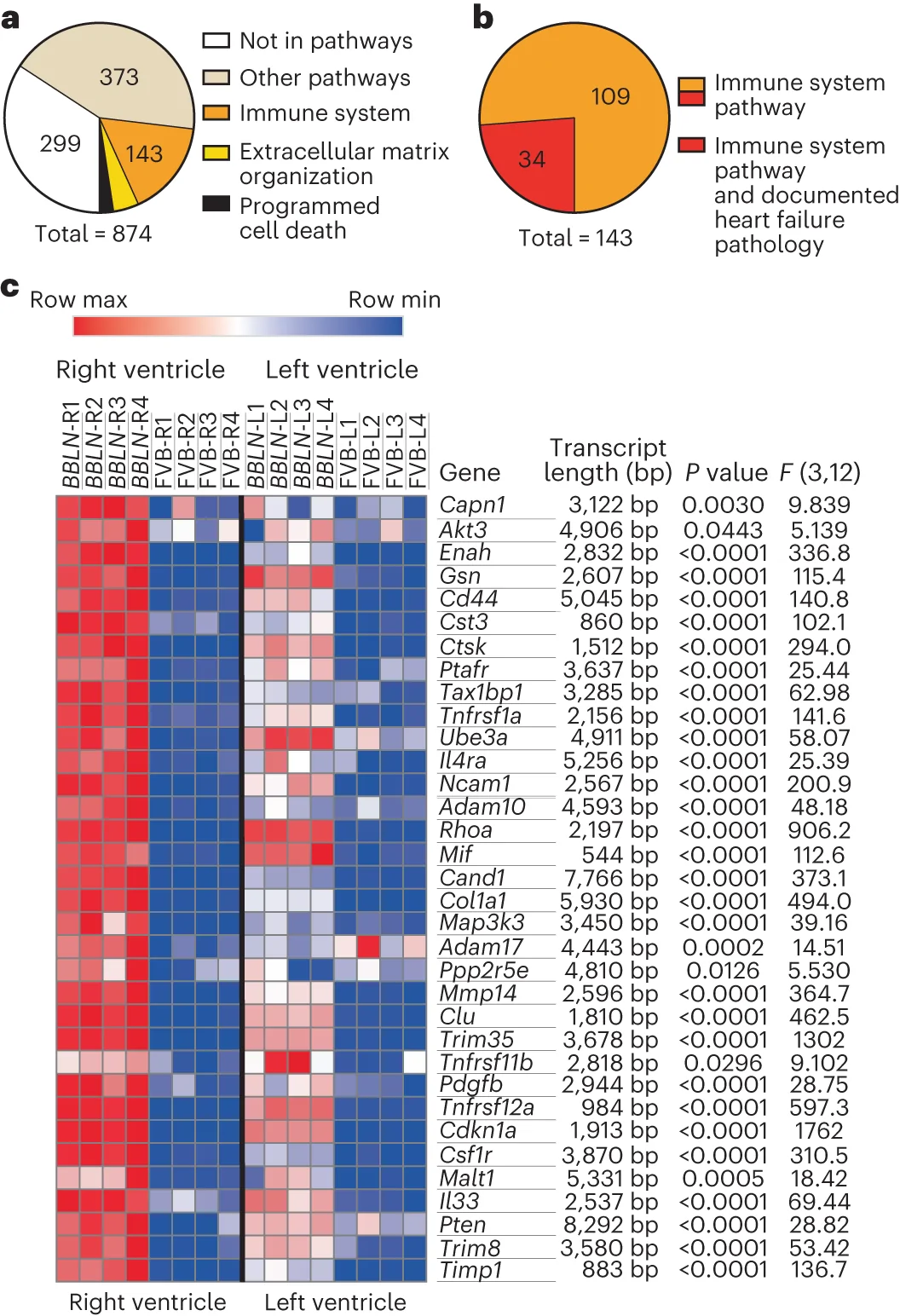
BBLN triggered heart failure-enhancing transcripts of the immune system pathway in right ventricular TOF patient heart specimens and right ventricles of Tg-*BBLN* mice. **a**, Overrepresentation analysis identified the immune system pathway (orange) as the predominant pathway with 143 transcripts having positive correlation with *BBLN* in RVOT specimens of TOF patients with cyanosis and upregulation in right ventricles of Tg-*BBLN* mice. **b**, Among the 143 *BBLN*-induced (Tg-*BBLN* mice) and *BBLN* positively correlated (TOF patients) transcripts in the immune system pathway, 34 transcripts have documented involvement in heart failure pathology (red). **c**, The heat map shows right and left ventricular NGS data of these heart failure-enhancing transcripts in Tg-*BBLN* and nontransgenic FVB mice. There was a predominant upregulation of heart failure-enhancing transcripts in right ventricular tissue of Tg-*BBLN* mice (Tg-1) compared with the left ventricular tissue. All transcript levels are significantly different between right ventricles of Tg-*BBLN* mice and right ventricles of nontransgenic FVB mice (*P* = 0.002979 *Capn1*, *P* = 0.04434 *Akt3*, *P* = 8.308 × 10^−12^ *Enah*, *P* = 2.803 × 10^−8^ *Gsn*, *P* = 8.21 × 10^−9^ *Cd44*, *P* = 9.194 × 10^−8^ *Cst3*, *P* = 6.21 × 10^−11^ *Ctsk*, *P* = 0.00002662 *Ptafr*, *P* = 1.004 × 10^−7^ *Tax1bp1*, *P* = 8.937 × 10^−9^ *Tnfrsf1a*, *P* = 4.113 × 10^−7^ *Ube3a*, *P* = 0.00004165 *Il4ra*, *P* = 6.08 × 10^−10^ *Ncam1*, *P* = 3.578 × 10^−7^ *Adam10*, *P* = 4.803 × 10^−13^ *Rhoa*, *P* = 8.234 × 10^−8^ *Mif*, *P* = 3.537 × 10^−12^ *Cand1*, *P* = 1.957 × 10^−12^ *Col1a1*, *P* = 0.000001511 *Map3k3*, *P* = 0.0002117 *Adam17*, *P* = 0.01257 *Ppp2r5e*, *P* = 6.772 × 10^−12^ *Mmp14*, *P* = 2.345 × 10^−12^ *Clu*, *P* = 4.519 × 10^−14^ *Trim35*, *P* = 0.02955 *Tnfrsf11b*, *P* = 0.00002365 *Pdgfb*, *P* = 1.232 × 10^−12^ *Tnfrsf12a*, *P* = 2.842 × 10^−14^ *Cdkn1a*, *P* = 2.409 × 10^−11^ *Csf1r*, *P* = 0.0004835 *Malt1*, *P* = 0.000002022 *Il33*, *P* = 0.000006043 *Pten*, *P* = 6.462 × 10^−7^ *Trim8* and *P* = 4.634 × 10^−9^ *Timp1*; one-way ANOVA and Tukey’s test; *n* = 4 male mice per group, age 3–4 months). Source data

**Extended Data Fig. 3:**
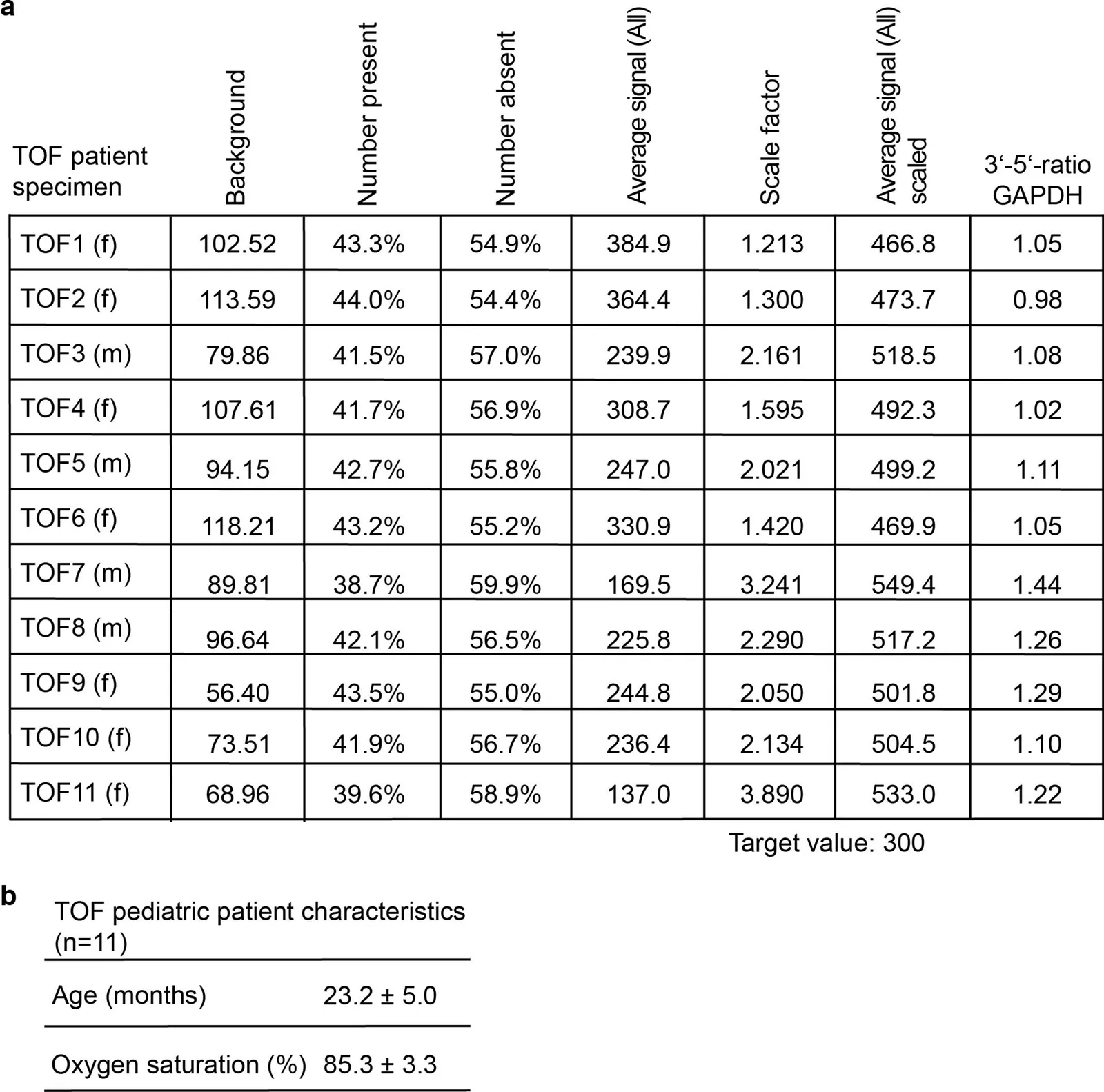
Whole genome microarray gene expression profiling of tetralogy of Fallot (TOF) patient heart specimens. **a**, Overview of microarray hybridization quality control features of whole genome microarray gene expression data of cardiac specimens from 11 patients diagnosed with TOF cardiac pathology and cyanosis (7 females, 4 males). Total cardiac RNA was extracted, processed for whole genome microarray gene expression profiling, and fragmented cRNA was hybridized to the gene chip (Affymetrix GeneChip Human Genome U133 Plus 2.0 Array). Raw data were analyzed with the GeneChip Operating Software (GCOS) and scaled to a target value of 300. **b**, Characteristics of 11 pediatric patients with TOF cardiac defects, from whom cardiac specimens were recovered for whole genome microarray gene expression profiling. Data are mean ± s.d.; n = 11 (7 females, 4 males). Source data

**Extended Data Fig. 4:**
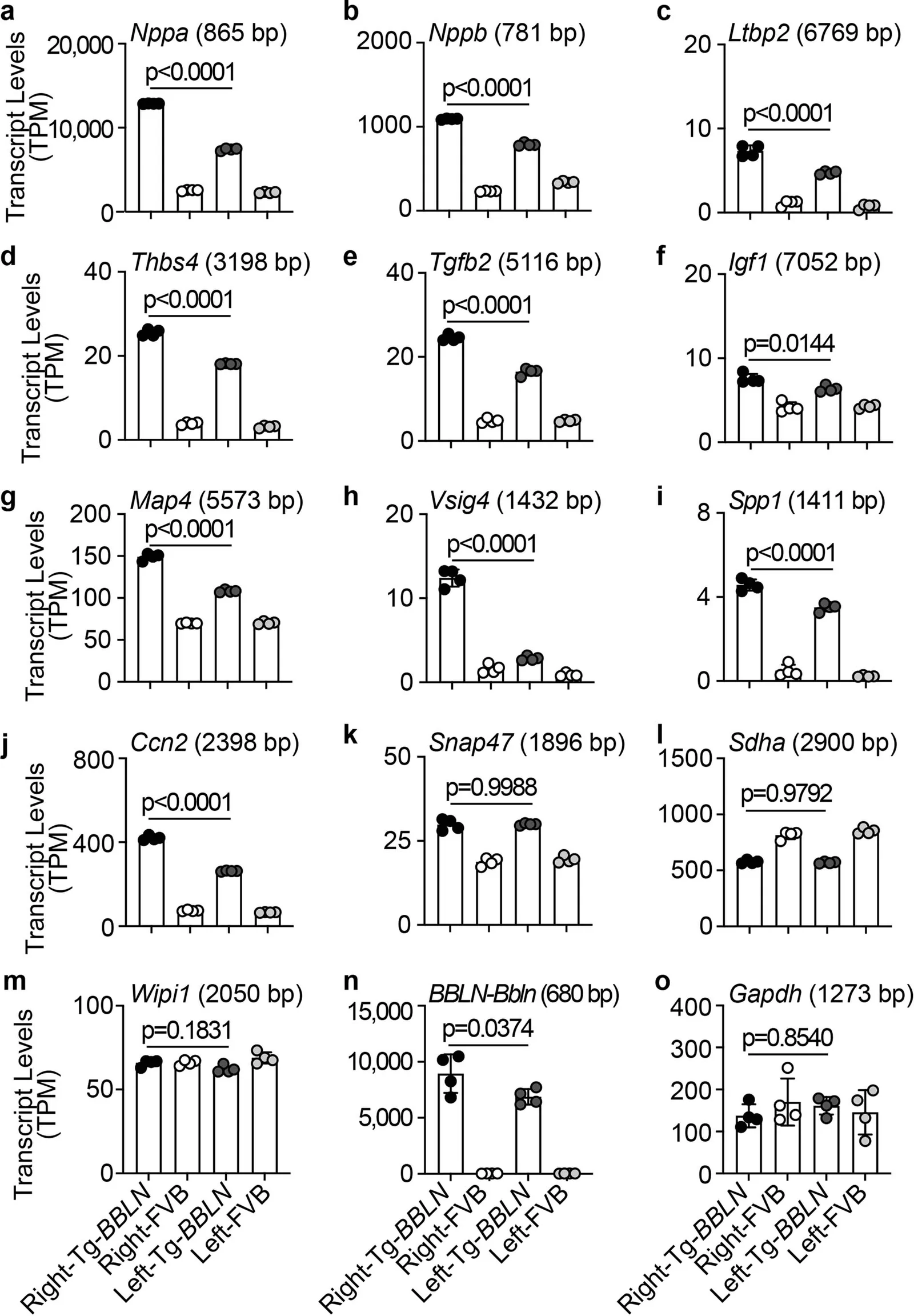
Validation of right ventricular NGS transcriptome profiling data by detection of heart failure-related differences between right and left ventricular heart specimens of Tg-*BBLN* mice. **a-j**, NGS transcriptome data of right and left ventricular heart specimens of Tg-*BBLN* mice (Tg-1) were validated by detection of differential gene expression between right and left ventricles. Transcript selection is based on literature data of differential gene expression between right and left ventricles from hypoxic rats, heart failure rats, and/or right ventricular heart failure patients^46–48^. Differences between right and left ventricles were attributed to the fact that the right ventricle has a lower capacity to adapt to systemic load and therefore maladaptive factors are higher in the right ventricle compared to the left ventricle^48^. Panels (**a**-**j**) show transcripts from Tg-*BBLN* mice with increased right ventricular expression levels compared to those of left ventricular heart tissue. **k**,**l**,**m**, *Snap47* levels were concordantly up-regulated (**k**), *Sdha* levels were concordantly down-regulated in right and left ventricles of Tg-*BBLN* mice compared to right and left ventricles of non-transgenic FVB mice (**l**), and *Wipi1* levels were not different between right and left ventricles of Tg-*BBLN* mice (**m**). **n**,**o**, As controls, increased transgenic *BBLN* levels in Tg-*BBLN* mice were comparable between right and left ventricles (**n**), and levels of the house-keeping gene, *Gapdh*, were not significantly different between right and left ventricles of Tg-*BBLN* mice and non-transgenic FVB mice (**o**). Data are mean ± s.d.; n = 4 male mice per group; age: 3–4 months. P-values for comparisons between right and left ventricular tissue of Tg-*BBLN* mice are indicated (one-way ANOVA and Tukey’s test; F(3,12) = 10797 (*Nppa*), p = 2.554e-14; 4161 (*Nppb*), p = 1.287e-12; 230.9 (*Ltbp2*), p = 0.000006432; 2452 (*Thbs4*), p = 0.000006432; 929.7 (*Tgfb2*), p = 2.535e-9; 51.13 (*Igf1*), p = 0.01436; 1061 (*Map4*), p = 3.773e-11; 322.5 (*Vsig4*), p = 1.707e-10; 397.5 (*Spp1*), p = 0.00008119; 3333 (*Ccn2*), p = 6.464e-13; 119.7 (*Snap47*), 0.9988; 162.5 (*Sdha*), p = 0.9792; 5.431 (*Wipi1*), p = 0.1831; 99.96 (*BBLN*; *Bbln*, *1110008P14Rik*), p = 0.03736; 0.4981 (*Gapdh*), p = 0.8540. Source data

To investigate the relationship between increased cardiac *BBLN* contents and TOF-related gene expression, we performed a whole-transcriptome analysis of RVOT specimens from TOF patients with cyanosis (Extended Data Fig. 3) and right ventricular heart specimens from Tg-*BBLN* mice (Extended Data Fig. 4). Next-generation sequencing (NGS) transcriptome profiling of right ventricular heart tissue of Tg-*BBLN* mice was validated by the detection of right ventricle-specific transcript changes in comparison with left ventricular tissue (Extended Data Fig. 4). Whole-transcriptome analysis found that about 60% of transcripts, which positively correlated with *BBLN* levels in TOF patient heart specimens with cyanosis (874 transcripts), also showed upregulation in the right ventricles of Tg-*BBLN* mice (Supplementary Dataset 1). The positive correlation of a transcript with cardiac *BBLN* transcript levels in TOF patient heart specimens is exemplified for the heart failure and myocardial ischemia marker, natriuretic peptide type b (*NPPB*) (Fig. 2h), which could be induced in TOF patients with cyanosis by the low oxygen condition^18,19^. As in TOF patient heart specimens with cyanosis, *Nppb* was also upregulated in right ventricular heart specimens of Tg-*BBLN* mice (Fig. 2i). The overrepresentation analysis of transcripts having positive correlation with *BBLN* levels in right heart specimens of TOF patients with cyanosis and showing upregulation in right ventricles of Tg-*BBLN* mice documented that *BBLN* triggered major biological pathways involved in heart remodeling (Figs. 2j and 3a and Supplementary Dataset 1). These pathways included the immune system pathway, the extracellular matrix organization pathway and the programmed cell death pathway with transcripts regulating apoptosis and necroptosis (Figs. 2j and 3a and Supplementary Dataset 1).

The immune system pathway was the major upregulated pathway triggered by *BBLN* in right TOF patient heart specimens and in right Tg-*BBLN* mouse hearts (Fig. 2j and Fig. 3a,b). Of those *BBLN* dependently upregulated cardiac transcripts of the ‘immune system’ pathway of TOF patients and Tg-*BBLN* mice, 23.8% have a documented pathological relevance for cardiac inflammation, cardiac remodeling and/or heart failure (Fig. 3b,c). The transcript intensity heat map documents the predominant upregulation of the heart failure-enhancing transcripts in right ventricles of Tg-*BBLN* mice compared with left cardiac ventricles (Fig. 3c). Thus, BBLN triggered the predominant upregulation of heart failure-enhancing transcripts in the right ventricles of Tg-*BBLN* mice and TOF patients.

The transcriptome analysis also found that 114 transcripts showed negative correlation with *BBLN* in TOF patients with cyanosis and downregulation in right ventricular heart specimens of Tg-*BBLN* mice (Supplementary Dataset 2). The overrepresentation analysis of those 114 transcripts displaying negative correlation with *BBLN* in TOF patient hearts and downregulation in Tg-*BBLN* mice revealed that increased *BBLN* led to downregulation of major cardiac metabolism pathways, such as the citric acid cycle and respiratory electron transport pathway, the mitochondrial translation pathway and the branched-chain amino acid catabolism pathway (Fig. 2j). Reduced mitochondrial respiratory chain activity, defective mitochondrial protein translation and impaired branched-chain amino acid catabolism are prominent features of patients with TOF and/or other forms of cardiomyopathy and heart failure^20–22^. Taken together, the right ventricular heart tissue of transgenic Tg-*BBLN* mice with increased cardiac *BBLN* contents reproduced major cardiac gene expression changes and alterations of biological pathways, which are characteristic of right ventricular TOF patient heart specimens.

## BBLN promoted CAMK2D activation in vivo

**Fig. 4:**
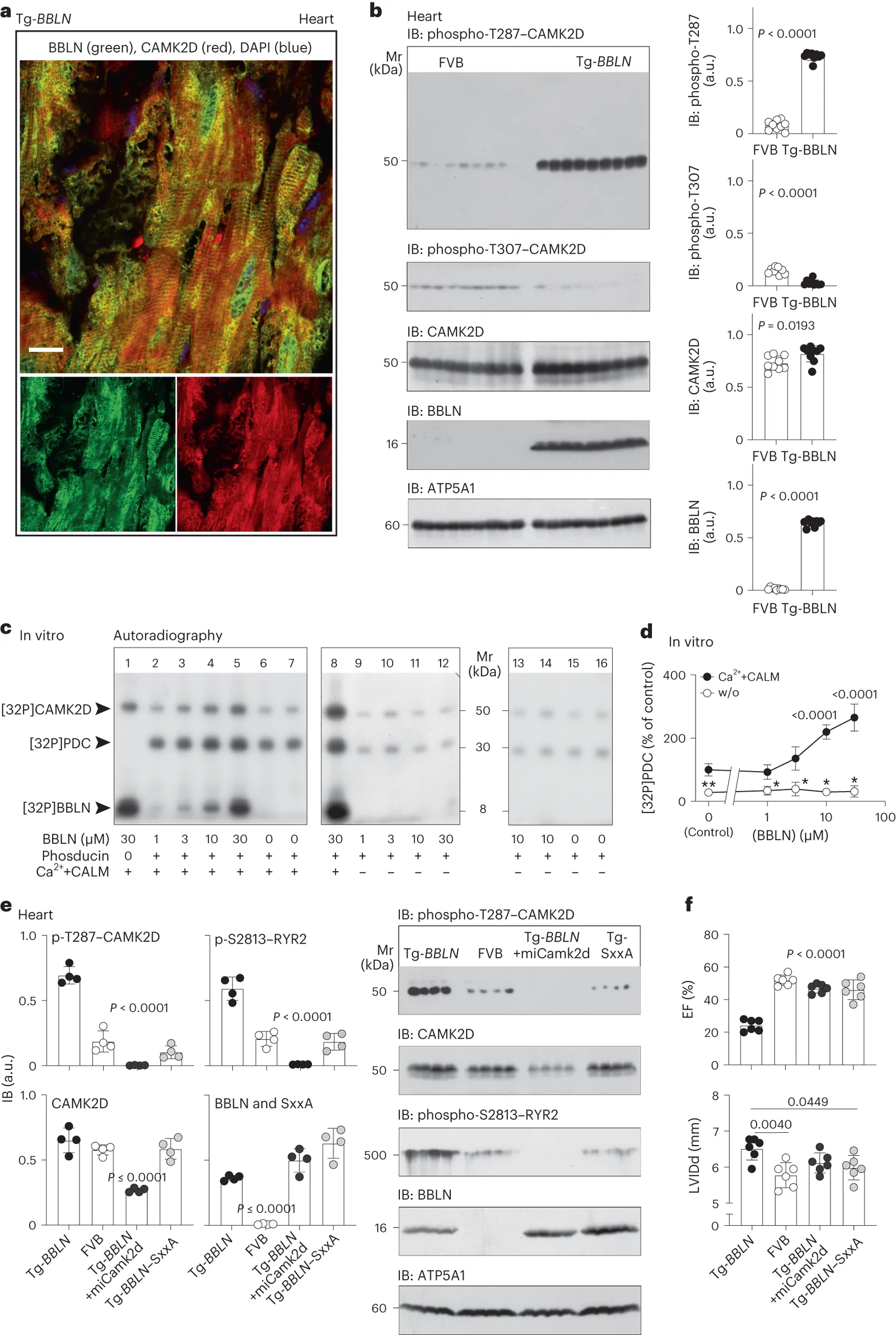
BBLN promoted CAMK2D activation in vivo and in vitro. **a**, Immunofluorescence colocalization of BBLN with CAMK2D on a heart specimen of an 8-month-old, male Tg-*BBLN* mouse (scale bar, 40 μm). The immunofluorescence colocalization was performed with heart specimens of five different Tg-*BBLN* mice (Tg-1) and five different nontransgenic FVB controls. All immunofluorescence images are shown in Supplementary Fig. 1. **b**, Quantitative IB determination of cardiac contents of activated phospho-T287–CAMK2D, inactive phospho-T307–CAMK2D, total CAMK2D and BBLN in 8-month-old, Tg-*BBLN* mice (Tg-1) and age- and sex-matched nontransgenic FVB control mice. The lower control IB detects ATP5A1. Left: IB images and right: quantitative IB data (mean ± s.d., *n* = 9 mice per group, 5 males and 4 females; unpaired, two-tailed *t*-test; d.f. 16, *t* = 32.92, 8.467, 2.602 and 56.01; *P* = 3.9625 × 10^−16^, 2.63699 × 10^−7^, 0.01927 and 8.64361 × 10^−20^). **c**,**d**, In vitro data show that recombinant BBLN protein enhanced the autophosphorylation of recombinant CAMK2D (200 nM) and the CAMK2D-mediated substrate phosphorylation of recombinant PDC and BBLN. Representative autoradiography images (**c**) and quantitative data (**d**) of BBLN-enhanced PDC phosphorylation by CAMK2D (50 nM) in vitro, in the presence and absence (w/o) of Ca^2+^+calmodulin (CALM) (mean ± s.d., *n* = 3 biological replicates, one-way ANOVA and Dunnett’s test; *F*(9,20) = 38.33; *P* = 0.9998, 0.3728, <0.0001 and <0.0001 BBLN+Ca^2+^+CALM versus Cont.+Ca^2+^+CALM; **P = 0.0052 versus Cont.+Ca^2+^+CALM; *P = 0.0182, 0.0321, 0.0113 and 0.0135 versus Cont.+Ca^2+^+CALM). **e**, Quantitative IB determination of cardiac contents of phospho-T287–CAMK2D, total CAMK2D, phospho-S2813–RYR2 and BBLN (and BBLN–SxxA) in 8-month-old, male Tg-*BBLN* mice (Tg-2), nontransgenic FVB mice and Tg-*BBLN* mice (Tg-2) after 4 weeks of lentiviral transduction of miCamk2d (Tg-*BBLN*+miCamk2d) and Tg-*BBLN*–SxxA mice. The control IB detects ATP5A1. Quantitative data (left) and IB images (right) (mean ± s.d.; *n* = 4 male, 8-month-old mice per group, one-way ANOVA and Tukey’s test; *F*(3,12) = 111.4, 27.29, 62.58 and 52.25; upper left: *P* = 1.914 × 10^−7^, 5.873 × 10^−9^ and 3.424 × 10^−8^; lower left: *P* = 0.4303, 0.00001428 and 0.5147; upper right: *P* = 0.000007374, 8.287 × 10^−8^ and 0.000004093; lower right: *P* = 0.0001016, 0.0933 and 0.001294); **f**, The left ventricular EF and the LVIDd of 8-month-old, male mice were determined by echocardiography (mean ± s.d., *n* = 6 mice per group, one-way ANOVA and Tukey’s test; *F*(3,20) = 54.28 and 5.562; *P* = 1.19 × 10^−9^, 3.58 × 10^−8^, 6.628 × 10^−8^ EF; *P* = 0.004012, 0.1793 and 0.0449 LVIDd). Source data

**Extended Data Fig. 5:**
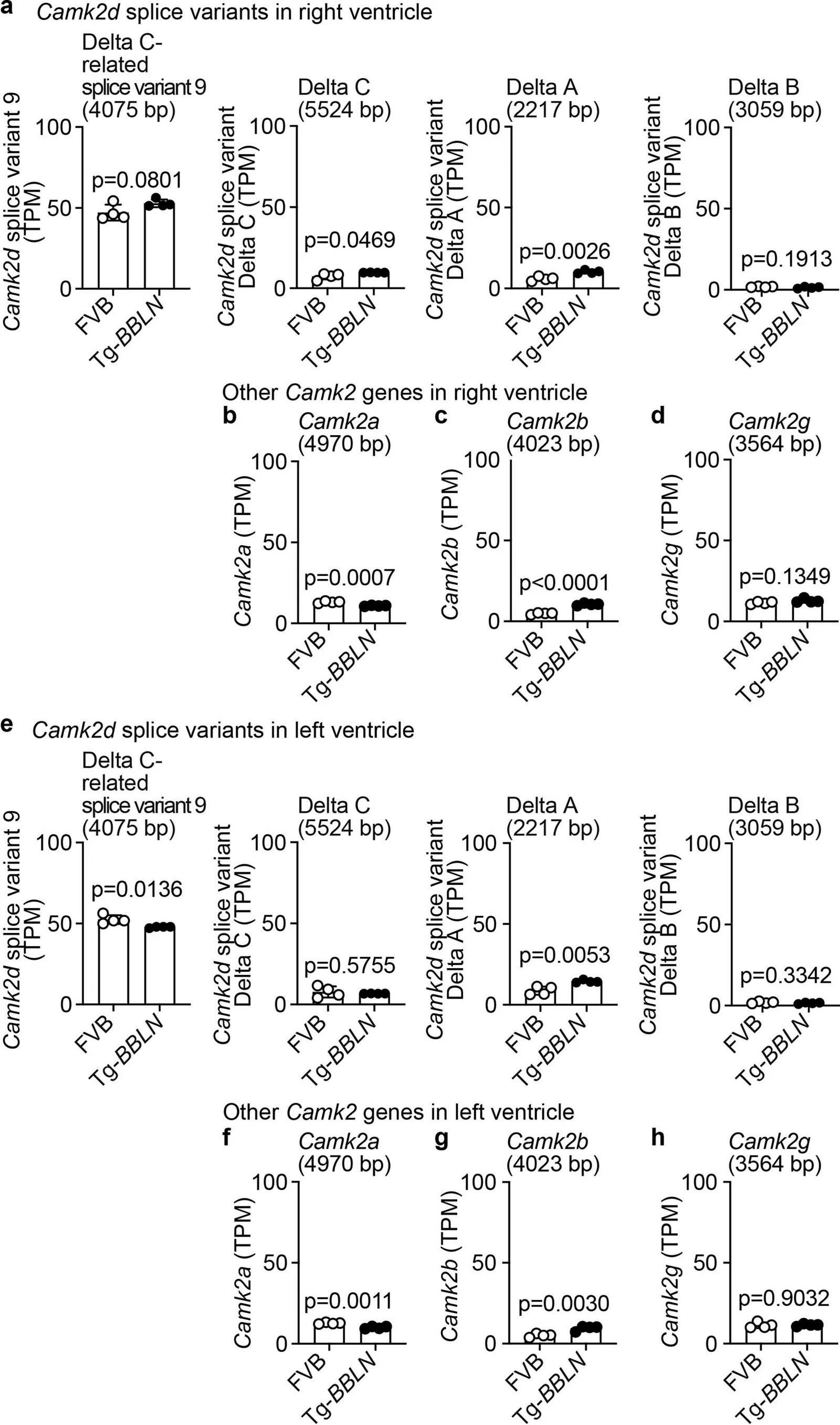
Cardiac transcript levels of *Camk2d* splice variants in right and left ventricles of Tg-*BBLN* and FVB mice. **a**,**e** Cardiac transcript levels of different *Camk2d* splice variants were determined by NGS in right (**a**) and left ventricles (**e**) of 3–4-month-old, male non-transgenic FVB mice and transgenic Tg-*BBLN* mice (Tg-1), and are presented as TPM (transcripts per million). **b**-**d**, **f-h**, Transcript levels of cardiac *Camk2a*, *Camk2b* and *Camk2g* were determined by NGS in right ventricular (**b-d**) and left ventricular heart specimens (**f-h)** of non-transgenic FVB mice and transgenic Tg-*BBLN* mice. Data are mean ± s.d. P-values were determined by the unpaired, two-tailed t-test; just alpha; n = 4 mice per group (df=6; **a**, t = 2.103, 2.494, 4.952, 1.473; p = 0.0801, 0.0469, 0.0026, 0.1913; **b**, t = 6.433; p = 0.0007; **c**, t = 13.39; p = 1.0757e-05; **d**, t = 1.727; p = 0.1349; **e**, t = 3.453, 0.5919, 4.256, 1.050; p = 0.0136, 0.5755, 0.0053, 0.3342; **f**, 5.857; p = 0.0011; **g**, 4.797; p = 0.0030; **h**, 0.1269; p = 0.9032). Source data

We searched for molecular mechanisms evoked by BBLN in the heart because the function of the human BBLN protein is largely unknown. BBLN was identified as a CAMK2D-interacting protein in TOF patient hearts (cf. Fig. 1). Immunofluorescence detected colocalization of BBLN with CAMK2D in areas with cardiac degeneration in Tg-*BBLN* heart specimens (Fig. 4a and Supplementary Fig. 1). In addition, Tg-*BBLN* mice showed increased cardiac CAMK2D activity with significantly elevated levels of autophosphorylated phospho-T287-CAMK2D, which were 9.2 ± 0.5-fold higher in Tg-*BBLN* hearts than in nontransgenic FVB controls (Fig. 4b). Of note, autophosphorylation of CAMK2D on threonine 287 causes autonomous kinase activity in the absence of its stimuli Ca^2+^ and calmodulin^23^. In contrast, the inactivating autophosphorylation of CAMK2D on T307 (and its neighbor T306), which occurs in the absence of calcium and prevents Ca^2+^/calmodulin binding^24^, was almost undetectable in Tg-*BBLN* hearts, whereas inactivating autophosphorylation on T307 was present in nontransgenic FVB hearts (Fig. 4b). For comparison, total CAMK2D protein levels of Tg-*BBLN* hearts were slightly but significantly (1.12-fold) above those of nontransgenic FVB controls (Fig. 4b). Likewise, cardiac transcript levels of the most abundant delta-C-related *Camk2d* splice variant 9 in the heart^12,25^ and of the delta-C splice variant were slightly (1.12- and 1.32-fold) increased in right ventricles of Tg-*BBLN* hearts (Extended Data Fig. 5a). Notably, these transcripts were only increased in right ventricles but not in left ventricles of Tg-*BBLN* hearts (Extended Data Fig. 5a,e).

**Extended Data Fig. 6:**
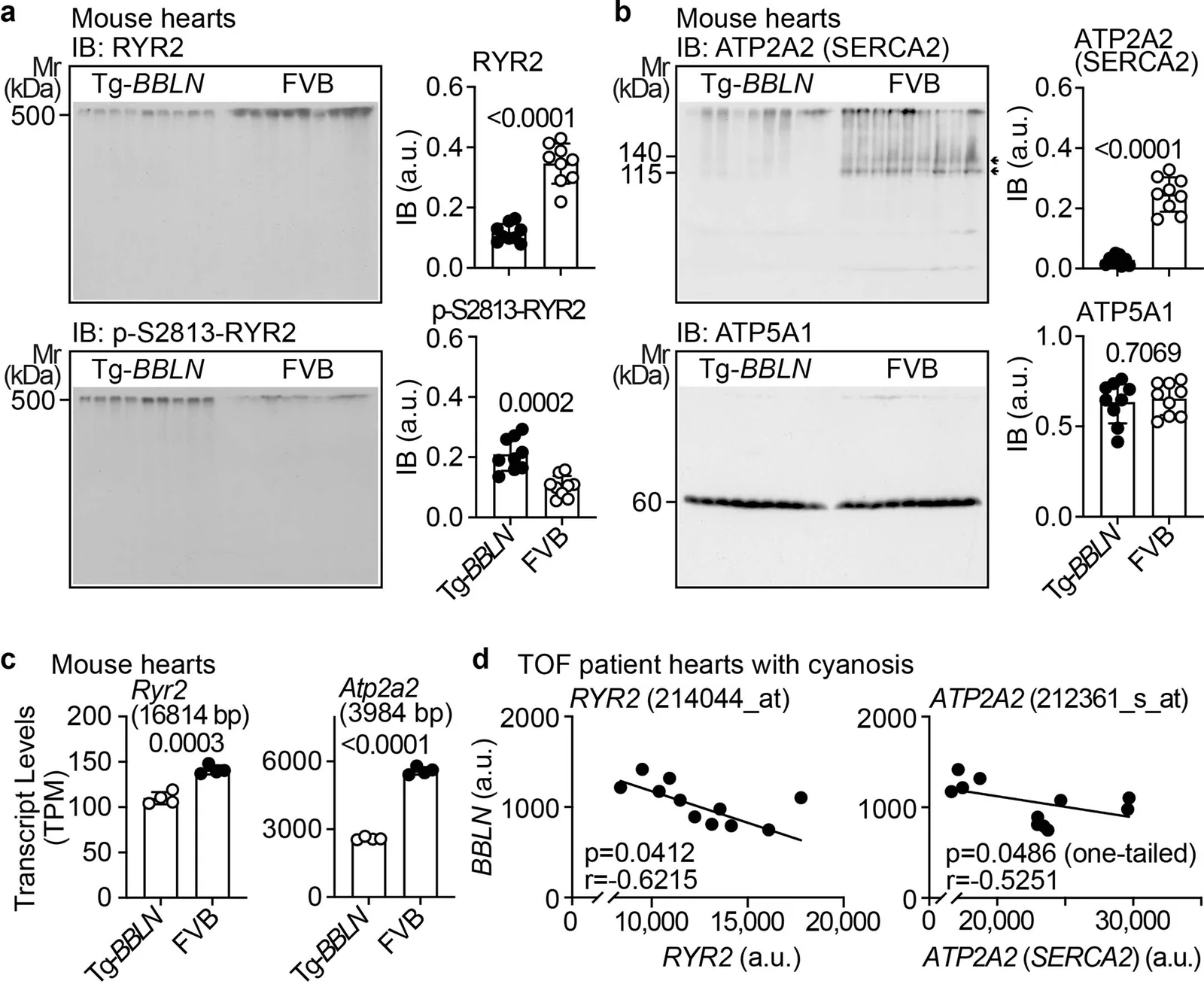
Enhanced CAMK2D-mediated phosphorylation of RYR2 in Tg-*BBLN* mice, and BBLN-dependent down-regulation of calcium handling proteins, RYR2 and SERCA2, in Tg-*BBLN* mice and TOF patients. **a**, Immunoblot analysis detected reduced total cardiac RYR2 protein contents and increased CAMK2D-mediated phosphorylation of RYR2 on serine-2813 in Tg-*BBLN* mice (Tg-1) compared to non-transgenic FVB controls. Representative immunoblots (left panels), and quantitative immunoblot data (right panels) are shown. **b**, Cardiac ATP2A2 (SERCA2) levels are decreased in Tg-*BBLN* (Tg-1) hearts. The lower control blot detects ATP5A1. Representative immunoblots (left panels), and quantitative data (right panels) are shown. Data (**a**,**b**) are mean ± s.d. (n = 9 mice per group, 5 male, 4 female; age: 8 months). P-values were determined by the unpaired, two-tailed t-test (df=16; **a**, t = 9.4818, 4.9131; p = 5.7293e-08, p = 0.000156; **b**, t = 11.09, 0.3828; p = 6.42141e-09, p = 0.7069). **c**, Transcript levels of *Ryr2* (left panel) and *Atp2a2* (right panel) were determined by NGS in heart specimens of Tg-*BBLN* mice (Tg-1) and non-transgenic FVB mice. Data are mean ± s.d. (n = 4 male mice per group; age: 8 months). P-values were determined by the unpaired, two-tailed t-test (df=6; t = 7.4612, p = 0.000299 (*Ryr2*); t = 33.28, p = 4.8989e-08 (*Atp2a2*)). **d**, *BBLN* transcript levels in right ventricular heart specimens of TOF patients with cyanosis were determined by microarray analysis (Affymetrix probe set ID: 204480_s_at) and negatively correlated with *RYR2* (214044_at) and *ATP2A2* (212361_s_at). Linear regression analysis was performed, and the Pearson correlation coefficient (r) and p-values (left panel, two-tailed, p = 0.0412; right panel, one-tailed, p = 0.0486) were determined (n = 11 TOF patients with cyanosis, 7 females, 4 males; age: 23.2 ± 5 months). Source data

Transcript levels of the *Camk2d* delta-A splice variant were low and slightly higher in both right and left ventricles of Tg-*BBLN* mice compared with those of nontransgenic FVB mice, whereas the delta-B splice variant was barely detectable (Extended Data Fig. 5a,e). Other *Camk2* genes (*Camk2a*, *Camk2b* and *Camk2g*) showed low cardiac expression levels (Extended Data Fig. 5b–d,f–h). Taken together, BBLN increased the cardiac levels of activated phospho-T287–CAMK2D. Concomitantly, CAMK2D substrate phosphorylation was also enhanced in Tg-*BBLN* hearts as exemplified for the ryanodine receptor 2 (RYR2) phosphorylation on serine 2813 (Extended Data Fig. 6a). Tg-*BBLN* hearts had increased contents of serine 2813-phosphorylated RYR2 (Extended Data Fig. 6a), which could contribute to BBLN-induced symptoms of heart failure^26^. Together, these experiments showed that BBLN enhanced the CAMK2D activity in vivo, in Tg-*BBLN* mice.

## BBLN enhanced CAMK2D activation in vitro

**Extended Data Fig. 7:**
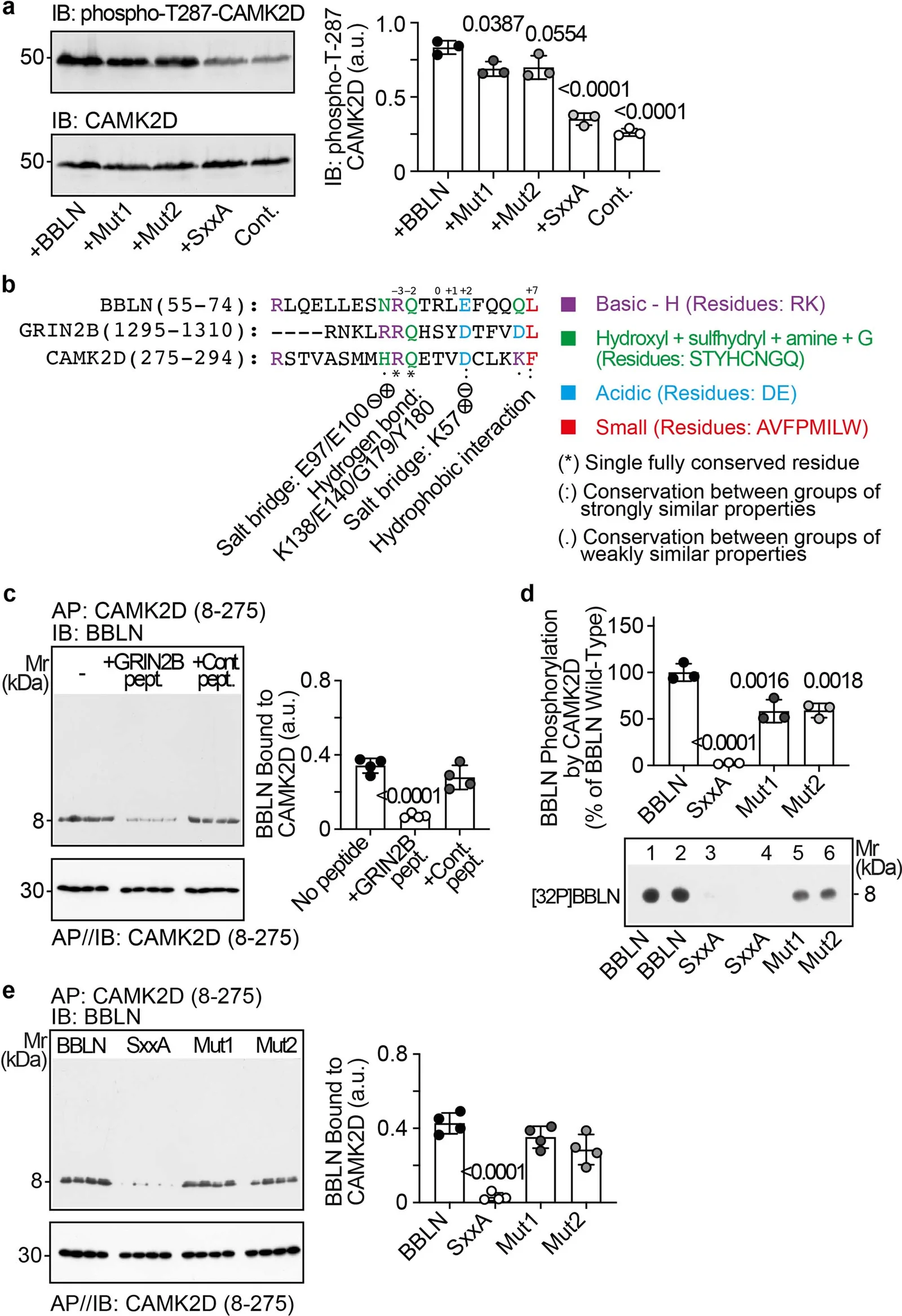
Enhancement of CAMK2D autophosphorylation on T287 by BBLN in vitro. **a**, Autophosphorylation of CAMK2D on T287 in the absence (Cont.) and presence of BBLN, BBLN-Mut1, BBLN-Mut2, and BBLN-SxxA (P-values vs. +BBLN). **b**, Sequence alignment of BBLN (residues 55–74) with the prototypical CAMK2 kinase domain-interacting region of GRIN2B (residues 1295–1310), and the regulatory domain residues 275–294 of CAMK2D was performed with CLUSTAL O. Potential interaction sites with CAMK2(D) kinase domain (based on ref. ^28^) are shown below the sequence alignment. **c**, Interaction of BBLN with CAMK2D kinase domain residues 8–275 is strongly reduced in the presence of the GRIN2B peptide (30 μM). CAMK2D kinase domain was immobilized on anti-FLAG affinity matrix (AP) and incubated with BBLN in the absence and presence of GRIN2B peptide or control peptide. After washing steps and protein elution with SDS-PAGE sample buffer, BBLN was detected by immunoblot (IB) with anti-BBLN antibody, and CAMK2D was detected by anti-CAMK2D antibody (lower blot) (P-value vs. no peptide and +Cont. pept.). **d**, In vitro phosphorylation assay of BBLN, BBLN-SxxA, BBLN-Mut1 and BBLN-Mut2 by CAMK2D. Phospho-site-deficient BBLN-SxxA was not phosphorylated (BBLN wild-type set to 100 %). **e**, Interaction of BBLN, BBLN-SxxA, BBLN-Mut1, and BBLN-Mut2 with CAMK2D residues 8–275 was determined by binding assay. CAMK2D kinase domain was immobilized on anti-Flag affinity matrix (AP), and incubated with BBLN and different BBLN mutants. After washing steps, proteins were eluted and quantitated by immunoblot (IB) (P-value vs. BBLN, BBLN-Mut1 and BBLN-Mut2). Data are mean ± s.d (**a**,**d**, n = 3; **c**,**e**, n = 4). P-values were determined by one-way ANOVA and Tukey’s test (**a**, F(4,10) = 69.97; p = 0.03874, 0.05538, 0.000003179, 6.453e-7 vs.+BBLN; **c**, F(2,9) = 40.32; p = 0.00003353 and 0.0002668 + GRIN2B pept. vs. no peptide and +Cont. pept. **d**, F(3,8) = 64.42; p = 0.000003474, 0.00164, 0.001832 vs. BBLN; **e**, F(3,12) = 34.40; p = 0.000003081, 0.00002688, and 0.000255 SxxA vs. BBLN, Mut1, and Mut2). Source data

In vitro experiments confirmed a causal relationship between BBLN and CAMK2D activity enhancement (Fig. 4c,d). Recombinant purified BBLN protein augmented the activating CAMK2D autophosphorylation on T287 in vitro (Fig. 4c and Extended Data Fig. 7a). BBLN also enhanced the CAMK2D-mediated substrate phosphorylation as proved with phosducin (PDC) (Fig. 4c,d), which was used as a CAMK2 substrate^27^. In addition, CAMK2D also phosphorylated BBLN (Fig. 4c). The CAMK2D-enhancing effect of BBLN was linked to the presence of Ca^2+^/calmodulin because without addition of Ca^2+^/calmodulin, the CAMK2D-enhancing effect of BBLN was abolished, and the basal CAMK2D autophosphorylation and substrate phosphorylation were strongly reduced (Fig. 4c,d).

Searching for the mode of interaction between BBLN and CAMK2D, we performed sequence alignment of BBLN with the prototypical CAMK2-interacting peptide of glutamate ionotropic receptor NMDA type subunit 2B (GRIN2B) residues 1,295–1,310, and with the regulatory segment residues 275–295 of CAMK2D. The alignment showed that the human BBLN amino acid sequence displays major features, which are typical for CAMK2 kinase domain interaction and which are present in CAMK2 substrates (Extended Data Fig. 7b). Notably, major interaction sites of GRIN2B (residues 1,295–1,310) and of the regulatory segment residues 275–295 with the CAMK2 kinase domain^28,29^ were present in BBLN residues 55–74 (Extended Data Fig. 7b). BBLN could thus interact with the previously identified binding site for activating substrates in the CAMK2(D) kinase domain^28^. In agreement with this notion, we found that the interaction of BBLN with the CAMK2D kinase domain was strongly reduced in the presence of the kinase domain-binding peptide of GRIN2B (Extended Data Fig. 7c).

We generated different BBLN mutants to analyze the interaction of BBLN with CAMK2D. The BBLN–SxxA mutant with removal of all phosphorylation sites (BBLN–S2A, Y28A, S33A, S40A, S62A, T66A, S79A and S82A) was not phosphorylated by CAMK2D (Extended Data Fig. 7d) and showed a strongly reduced interaction with the CAMK2D kinase domain, as determined by co-enrichment (Extended Data Fig. 7e). For comparison, BBLN–Mut1 (BBLN–S2A, T66A, S79A and S82A) and BBLN–Mut2 (Y28A, S33A and S40A) showed a partially reduced phosphorylation by CAMK2D (Extended Data Fig. 7d). However, the interaction with the CAMK2D kinase domain was still effective and comparable to wild-type BBLN (Extended Data Fig. 7e). This observation is in agreement with other CAMK2 substrates such as GRIN2B, in which exchange of a single CAMK2 phosphorylation site to alanine did not disrupt the binding to CAMK2 (ref. ^30^). The BBLN–SxxA mutant with impaired interaction with the CAMK2D kinase domain also showed a strongly reduced CAMK2D-enhancing effect in vitro, whereas BBLN–Mut1 and BBLN–Mut2 were still able to enhance CAMK2D autophosphorylation on T287 (Extended Data Fig. 7a). Together, these in vitro data strongly indicate that BBLN functions similarly as other CAMK2-activating substrates^28^. BBLN does not induce the active ‘on’ state but could sustain/enhance the activating CAMK2D autophosphorylation on T287, which is triggered by the presence of Ca^2+^/calmodulin.

## BBLN triggered cardiac dysfunction by CAMK2D activation

**Extended Data Fig. 8:**
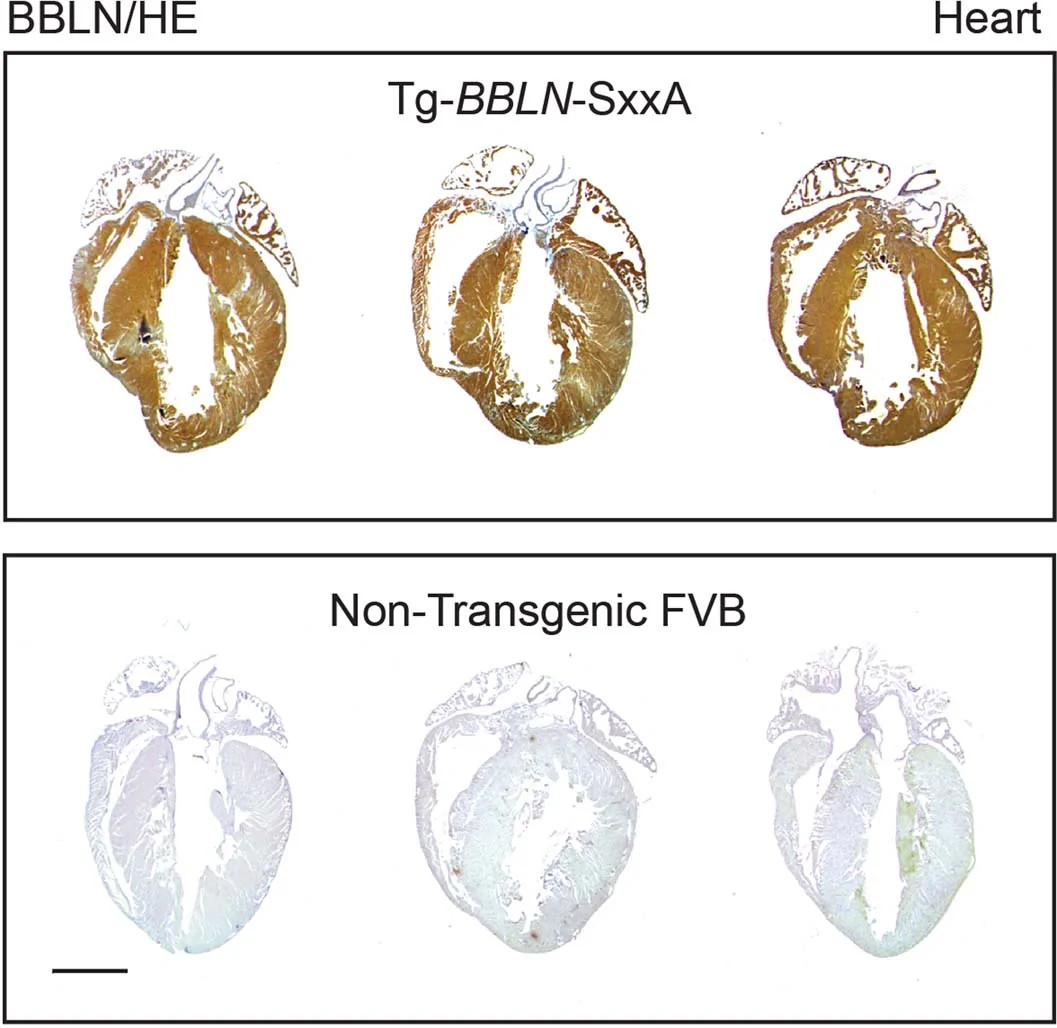
Immuno-histological analysis of Tg-*BBLN*-SxxA mouse hearts. The cardiac BBLN (and BBLN-SxxA) protein was detected on cardiac specimens of 8-month-old, male Tg-*BBLN*-SxxA mice (upper panels) and age- and sex-matched non-transgenic FVB mice (lower panels) by immunohistology with anti-BBLN antibodies. Counterstaining was performed with hematoxylin (HE); bar: 2 mm; n = 3 mice/group.

In vivo experiments confirmed a major role of BBLN-stimulated CAMK2D activity in the pathologic cardiac phenotype of Tg-*BBLN* mice because downregulation of the CAMK2D protein by lentiviral transduction of a *Camk2d*-targeting micro (mi)RNA prevented the BBLN-induced increase in cardiac contents of autophosphorylated phospho-T287–CAMK2D, reduced the cardiac phosphorylation of the CAMK2D substrate RYR2 on S2813 and retarded the BBLN-induced cardiac dysfunction (Fig. 4e,f). Likewise, the BBLN–SxxA mutant, which was ineffective in vitro, did not enhance CAMK2D autophosphorylation and CAMK2D substrate phosphorylation in vivo, in transgenic Tg-*BBLN–SxxA* mice (Fig. 4e). Moreover, the inactive BBLN–SxxA mutant did not cause cardiac dysfunction and cardiac hypertrophy (Fig. 4f and Extended Data Fig. 8). Nevertheless, at present, we cannot completely rule out that CAMK2(D)-independent effects may contribute to the unaltered cardiac phenotype of Tg-*BBLN–SxxA* mice.

**Extended Data Fig. 9:**
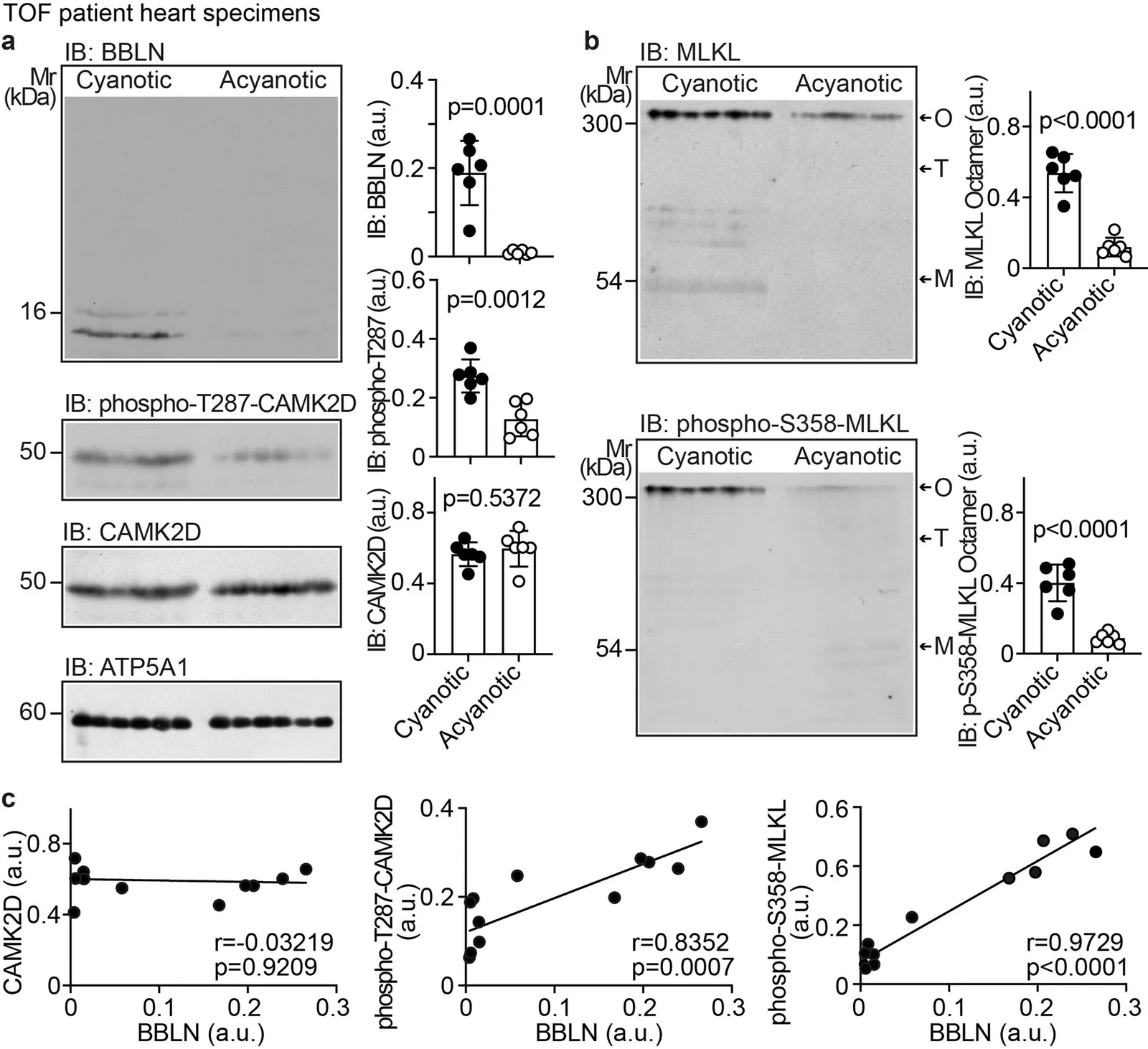
Immunoblot detection of activated phospho-T287-CAMK2D and necroptotic features in TOF patient heart specimens. **a**, Cardiac contents of BBLN, phospho-T287-CAMK2D, and total CAMK2D were determined by immunoblot (IB) of cardiac proteins in heart specimen lysates from cyanotic and acyanotic TOF patients. The left panels show immunoblot images, and the right panels show quantitative data. The lower control immunoblot detects ATP5A1. **b**, Immunoblot detection of MLKL and phospho-S358-MLKL octamers in heart specimen lysates of cyanotic and acyanotic TOF patients. Data (**a**, **b**) are mean ± s.d. P-values were determined by the unpaired, two-tailed t-test (n = 6 patients per group, 3 females, 3 males; age cyanotic TOF patients: 23.17 ± 4.5 months; age acyanotic TOF patients: 29.5 ± 7.6 months; df=10; **a**, t = 6.0614, 4.4782, 0.6390; p = 0.0001218, 0.001182, 0.5372; **b**, t = 8.4943, 7.09; p = 0.000006939, 0.00003335). **c**, Correlation analyses were performed between cardiac contents of BBLN and total CAMK2D (left), BBLN and phospho-T287-CAMK2D (middle), and BBLN and phospho-S358-MLKL (right). Pearson correlation (r) and p-values (two-tailed) were determined of n = 6 cyanotic and 6 acyanotic TOF patients (left panel: p = 0.9209; middle panel: p = 0.0007209; right panel: p = 1.095e-7). Source data

Similar to transgenic Tg-*BBLN* mouse hearts, cardiac specimens of human TOF patients with increased BBLN contents and cyanosis also showed increased phospho-T287–CAMK2D contents compared with those of acyanotic TOF patients, whereas total CAMK2D protein levels were not significantly different between the two groups (Extended Data Fig. 9a). Taken together, our data strongly suggest that BBLN triggered cardiac dysfunction and cardiac hypertrophy by CAMK2D activation.

## BBLN induced the cardiac inflammasome and cardiac fibrosis

**Fig. 5:**
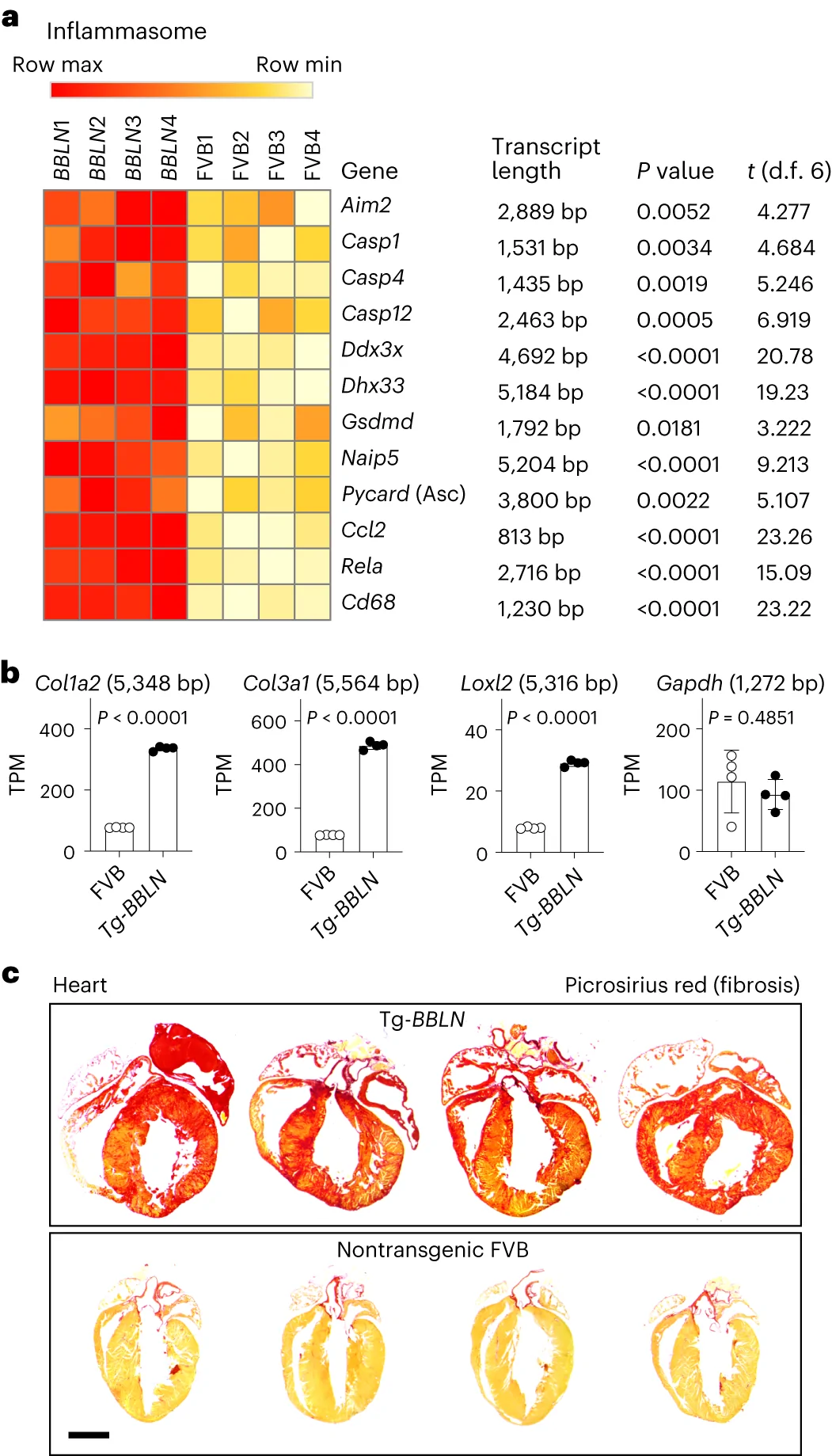
BBLN-induced upregulation of cardiac inflammasome transcripts and cardiac fibrosis. **a**, Upregulated cardiac inflammasome transcripts in transgenic, 8-month-old, male Tg-*BBLN* mice (Tg-1) compared with age- and sex-matched, nontransgenic FVB mice were identified by Gene Ontology (GO) analysis of NGS transcriptome data. The heat map shows members of the canonical inflammasome complex (GO: 0061702) and proinflammatory CAMK2D-regulated transcripts of ref. 31. *P* values were determined by MeV (unpaired, two-tailed *t*-test, just alpha; *n* = 4 mice per group; d.f. 6; *P* = 0.005223, 0.003382, 0.001927, 0.000451, 8.083 × 10^−7^, 0.00000128, 0.01809, 0.00009222, 0.002207, 4.136 × 10^−7^, 0.000005349 and 4.188 × 10^−7^). **b**, NGS data of prototypical transcripts involved in fibrotic cardiac remodeling of 8-month-old, male Tg-*BBLN* mice (Tg-1) compared with age- and sex-matched nontransgenic FVB mice. *P* values were determined by an unpaired, two-tailed *t*-test (*n* = 4 mice per group; d.f. 6; *t* = 70.86, 47.35, 40.73 and 0.7437; *P* = 5.316 × 10^−10^, 5.946 × 10^−9^, 1.465 × 10^−8^ and 0.4851). **c**, Picrosirius red staining of paraffin-embedded heart sections shows prominent fibrotic cardiac remodeling of 8-month-old, male Tg-*BBLN* mice (Tg-1) compared with age- and sex-matched, nontransgenic FVB mice (*n* = 4 mouse hearts per group; scale bar, 2 mm). Source data

The CAMK2D-induced cardiac dysfunction involves proinflammatory immune system-mediated, fibrotic cardiac remodeling^31^. Likewise, BBLN as a CAMK2D enhancer, triggered the immune system pathway, with prominent upregulation of heart failure-enhancing transcripts in Tg-*BBLN* mice and TOF patient heart specimens with cyanosis (cf. Figs. 2j and 3). A specific search for upregulated inflammasome-related transcripts identified eight members of the canonical inflammasome complex (GO:0061702) and four additional proinflammatory CAMK2D-regulated transcripts^31^ in Tg-*BBLN* hearts (Fig. 5a). Concomitantly, transcripts detecting fibrotic cardiac remodeling^32^ were increased, for example, *Col1a2*, *Col3a1* and the collagen-cross-linking enzyme, lysyl oxidase-like 2, *Loxl2* (Fig. 5b). Together with upregulated inflammatory and profibrotic transcripts, Tg-*BBLN* hearts had cardiac fibrosis as detected by picrosirius red staining of collagen fibers (Fig. 5c).

**Extended Data Fig. 10:**
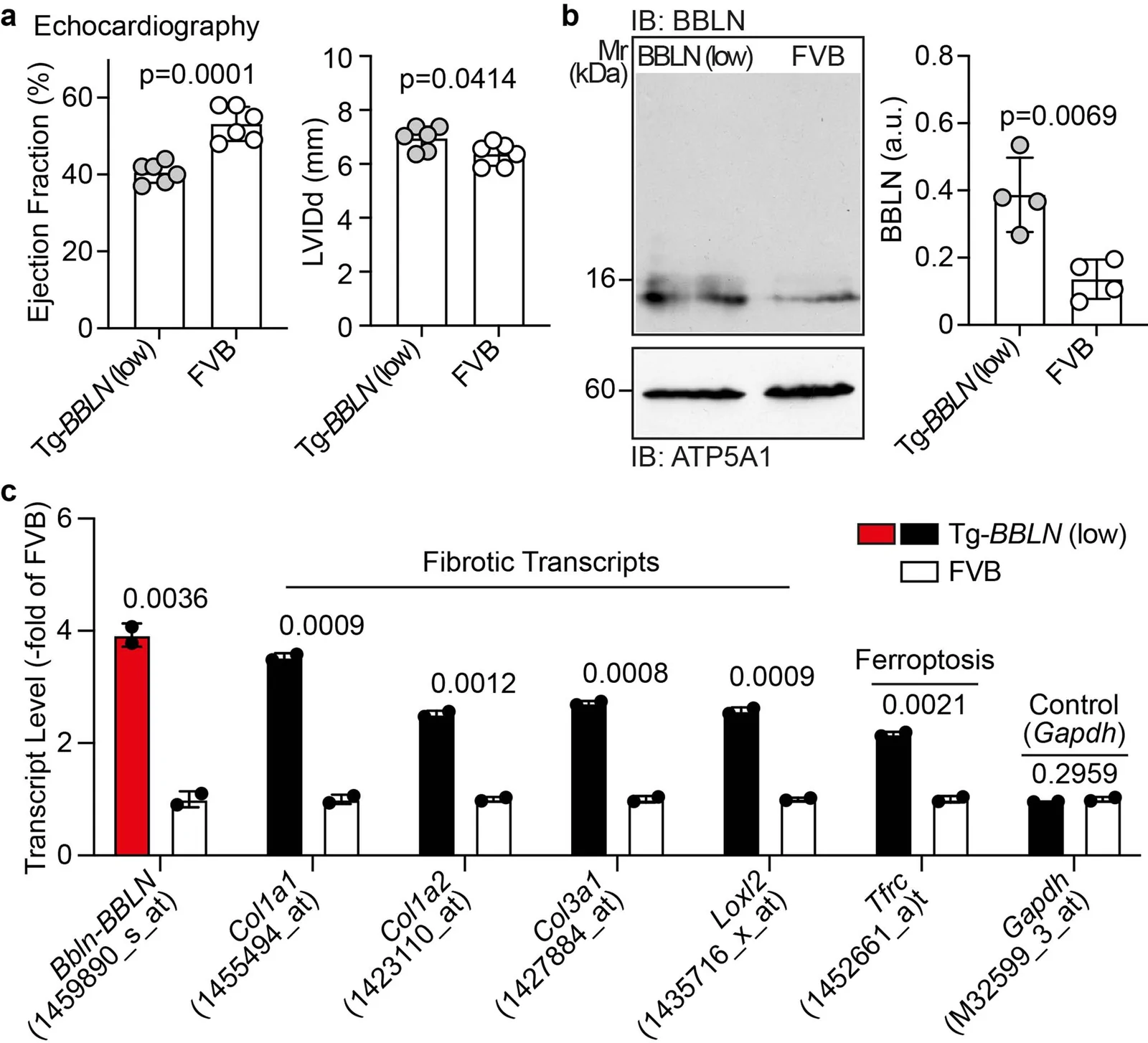
Cardiac dysfunction and up-regulation of cardiac remodeling transcripts in Tg-*BBLN* (low) hearts with 4-fold increased BBLN protein levels. **a**, Echocardiography documents a significantly reduced left ventricular ejection fraction (**a**, left panel) and cardiac dilation with increased left ventricular diameter in diastole (**a**, right panel) in Tg-*BBLN* (low) mice with 4-fold increased cardiac BBLN protein contents in comparison to age- and sex-matched non-transgenic FVB mice. Data are mean ± s.d., n = 6 male mice per group, age 8 months (unpaired, two-tailed t-test; df=10; t = 5.986, 2.3392; p = 0.0001346, p = 0.04139). **b**, Cardiac BBLN protein contents were determined by immunoblot (IB) in hearts of Tg-*BBLN* (low) mice in comparison to those of non-transgenic FVB mice (mean ± s.d.; n = 4 male mice per group; age 8 months; mean ± s.d.; unpaired, two-tailed t-test; df=6; t = 4.024; p = 0.006927). **c**, Whole genome microarray gene expression profiling detects up-regulation of fibrotic remodeling transcripts in Tg-*BBLN* (low) hearts with 4-fold increased *Bbln*-*BBLN* transcript levels compared to those of non-transgenic FVB hearts. As a control, *Gapdh* transcript levels were not different between groups. Data are mean ± s.d. (n = 4 mouse hearts per gene chip; 2 gene chips per group; unpaired, two-tailed t-test, just alpha; df=2; t = 16.61, 32.39, 28.01, 34.2, 31.98, 21.92, 1.4024; p = 0.003607, 0.0009516, 0.001272, 0.0008539, 0.0009761, 0.002075, 0.2959). Source data

Upregulation of key transcripts of cardiac fibrosis and heart remodeling such as *Col1a1*, *Col1a2*, C*ol3a1* and the collagen-cross-linking enzyme *Loxl2* were also detected in Tg-*BBLN* hearts with only a four-fold increase in BBLN protein levels (Extended Data Fig. 10a–c). Moreover, these mice with moderately increased cardiac BBLN levels developed features of compensated heart failure at an age of 8 months, with a significantly reduced left ventricular EF of 40.5 ± 2.7% compared with 53.2 ± 4.5% of nontransgenic FVB mice, and showed a significant cardiac enlargement in echocardiographic examination (Extended Data Fig. 10a,b). Thus, moderately elevated cardiac BBLN levels are sufficient to cause fibrotic cardiac remodeling and features of heart failure.

## BBLN enhanced CAMK2D-mediated features of necroptosis

**Fig. 6:**
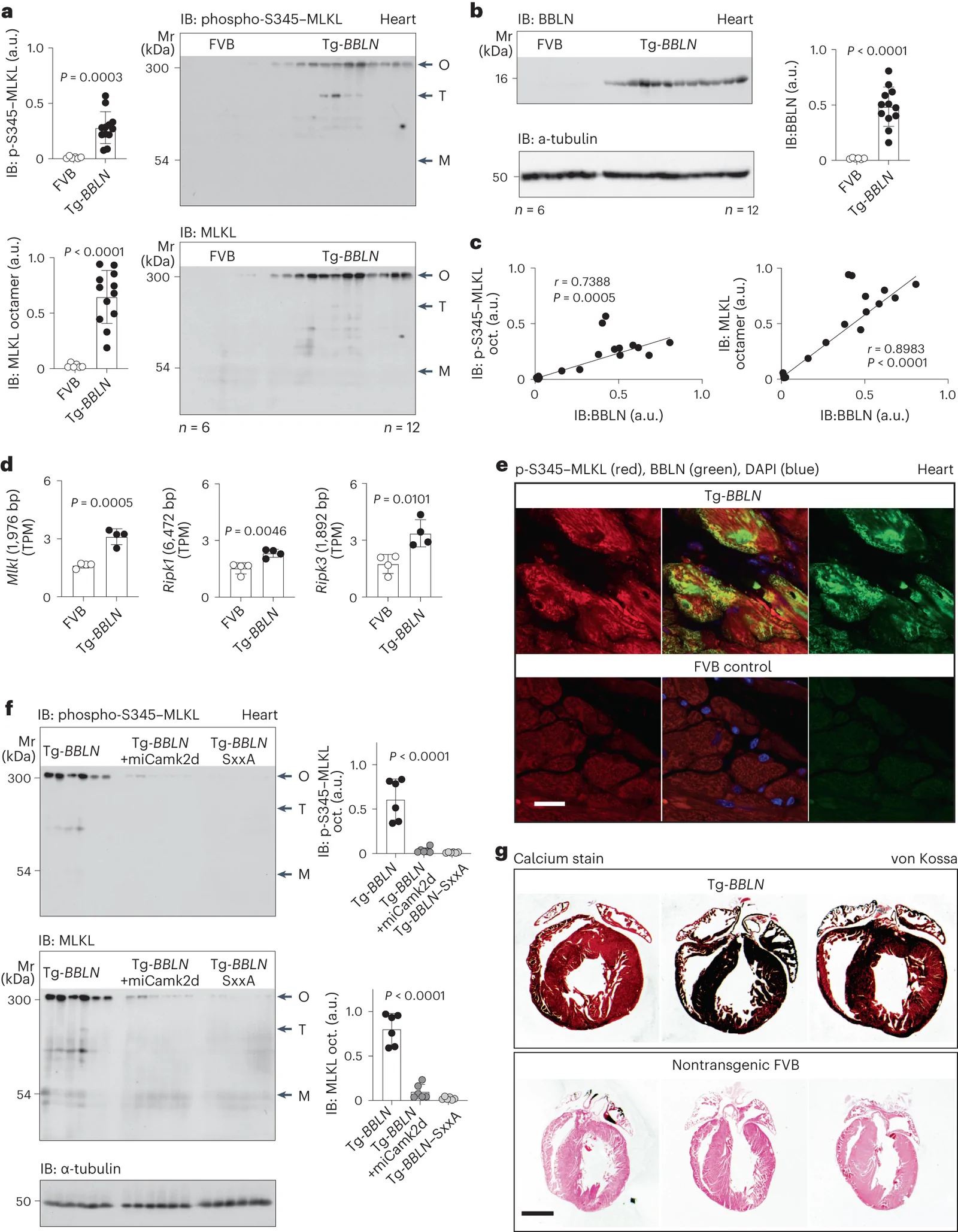
BBLN enhanced CAMK2D-mediated features of necroptosis. **a**, IB determination of cardiac phospho-S345–MLKL (top) and total MLKL (bottom) contents of 8-month-old, nontransgenic FVB and transgenic Tg-*BBLN* mice. Predominant MLKL octamers as a feature of necroptosis were quantified. MLKL octamers (O), tetramers (T) and monomers (M) are marked by arrows. Quantitative IB data (left) and IB images (right) (mean ± s.d., *n* = 6 FVB mice, 3 male and 3 female, and *n* = 12 Tg-*BBLN* mice (Tg-2), 6 male and 6 female; unpaired, two-tailed *t*-test; d.f. 16, *t* = 4.521 and 6.240; *P* = 0.000348 and 0.00001181). **b**, IB determination of cardiac BBLN contents in FVB and Tg-*BBLN* mice, which were used in **a**. IB detection of BBLN (top left) and quantitative IB data (right) (mean ± s.d., *n* = 6 FVB, 3 male and 3 female, and *n* = 12 Tg-*BBLN* mice, 6 male and 6 female; unpaired, two-tailed *t*-test; d.f. 6, *t* = 6.354; *P* = 0.000009572). The lower left control IB detects α-tubulin. **c**, Linear regression analysis between cardiac BBLN contents and phospho-S345–MLKL octamers (left), and cardiac BBLN contents and MLKL octamers (right). Linear regression analysis was performed, and the Pearson correlation coefficients (*r*) and *P* values (two tailed) were determined (*n* = 18, that is, 6 FVB and 12 Tg-*BBLN* hearts; *P* = 0.0005 and <0.0001). **d**, Cardiac transcript levels (TPM) of *Mlkl*, *Ripk1* and *Ripk3* were determined by NGS (mean ± s.d., unpaired, two-tailed *t*-test, just alpha; *n* = 4 male mice per group; age: 8 months; d.f. 6, *t* = 6.917, 4.3836 and 3.696; *P* = 0.0004517, 0.00465 and 0.01014). **e**, Immunofluorescence colocalization of BBLN with p-S345–MLKL on heart specimens of an 8-month-old, male Tg-*BBLN* mouse (Tg-2) and an age- and sex-matched, nontransgenic FVB mouse (scale bar, 40 μm). The immunofluorescence is representative of four mouse hearts per group. **f**, Quantitative IB determination of phospho-S345–MLKL and total MLKL octamers (O) in heart lysates of Tg-*BBLN* (Tg-2), Tg-*BBLN*+miCamk2d and Tg-*BBLN–*SxxA mice. IB images (left) and quantitative data (right) (mean ± s.d.; *n* = 6 male mice per group; age 8 months; one-way ANOVA and Tukey’s test; *F*(2,15) = 37.78 and 88.23; *P* = 0.000006554 and 0.00000408 Tg-*BBLN* versus the two other groups (top) *P* = 4.714 × 10^−8^ and 1.184 × 10^−8^ Tg-*BBLN* versus the two other groups (bottom)). The lower control IB detects α-tubulin. **g**, Detection of calcium deposits by von Kossa calcium staining on cardiac specimens of Tg-*BBLN* and nontransgenic FVB control mice (*n* = 3 male mice per group; age 8 months; scale bar, 2 mm). Source data

The transcriptome analysis also detected BBLN-dependent upregulation of the programmed cell death pathway (including apoptosis and necroptosis) in TOF patient heart specimens and Tg-*BBLN* mouse hearts (cf. Fig. 2j). Among different forms of programmed cell death, necroptosis is an important CAMK2D-induced pathomechanism of cell death in the ischemic and failing heart^33–37^. Tg-*BBLN* mice had increased cardiac contents of aggregated and activated phospho-S345–mixed lineage kinase domain-like protein (MLKL) octamers (Fig. 6a), which are the effectors of necroptosis^33–37^. The cardiac contents of phosphorylated p-S345–MLKL octamers and total MLKL octamers were positively correlated with cardiac BBLN protein levels (Fig. 6b,c). Tg-*BBLN* hearts showed upregulation of the *Mlkl* transcript together with the necrosome assembly protein, receptor-interacting protein kinase 1 (*Ripk1*), and the MLKL-phosphorylating Ripk3 (Fig. 6d). The increased BBLN protein level could contribute to necrosome assembly and necroptosis by enhancement of CAMK2D kinase activity, which is directly involved in necroptosis^33^. In agreement with this notion, BBLN was colocalized with phospho-S345–MLKL in necroptotic areas of failing Tg-*BBLN* hearts with cardiac tissue damage (Fig. 6e). In addition, the BBLN-triggered induction of phospho-S345–MLKL octamers was retarded by RNAi-mediated downregulation of *Camk2d* by lentiviral transduction of miCamk2d (Fig. 6f). As a control, the phosphorylation-deficient, inactive BBLN–SxxA mutant did not cause features of necroptosis (Fig. 6f). Similar to Tg-*BBLN* mice, TOF patient heart specimens with BBLN upregulation also showed higher levels activated phospho-T287–CAMK2D and phospho-S358–MLKL octamers (Extended Data Fig. 9a,b). There was a positive correlation between BBLN and phospho-T287–CAMK2D, and between BBLN and phospho-S358–MLKL octamers in TOF patient heart specimens (Extended Data Fig. 9c). Together, these data show that BBLN-enhanced CAMK2D activation promoted features of cardiac necroptosis.

## BBLN-triggered calcium overload and DES degradation

Concomitant with necroptotic cell death, which is associated with calcium overload^37^, Tg-*BBLN* hearts were characterized by calcium overload, which was detected by von Kossa staining (Fig. 6g). Calcium accumulation was accompanied by disturbance of the intracellular calcium-handling proteins in Tg-*BBLN* hearts (Extended Data Fig. 6). Notably, the cardiac RYR2 and SERCA2 (ATP2A2) contents were downregulated at the protein and transcript level in Tg-*BBLN* hearts (Extended Data Fig. 6a–c). Likewise, in TOF patient hearts, the *RYR2* and *SERCA2* transcript levels were negatively correlated with *BBLN* (Extended Data Fig. 6d). With these features, Tg-*BBLN* mouse hearts and human heart specimens from TOF patients with cyanosis resemble patients with ischemic cardiomyopathy, whose hearts also show decreased RYR2 and SERCA2 levels^38,39^.

**Fig. 7:**
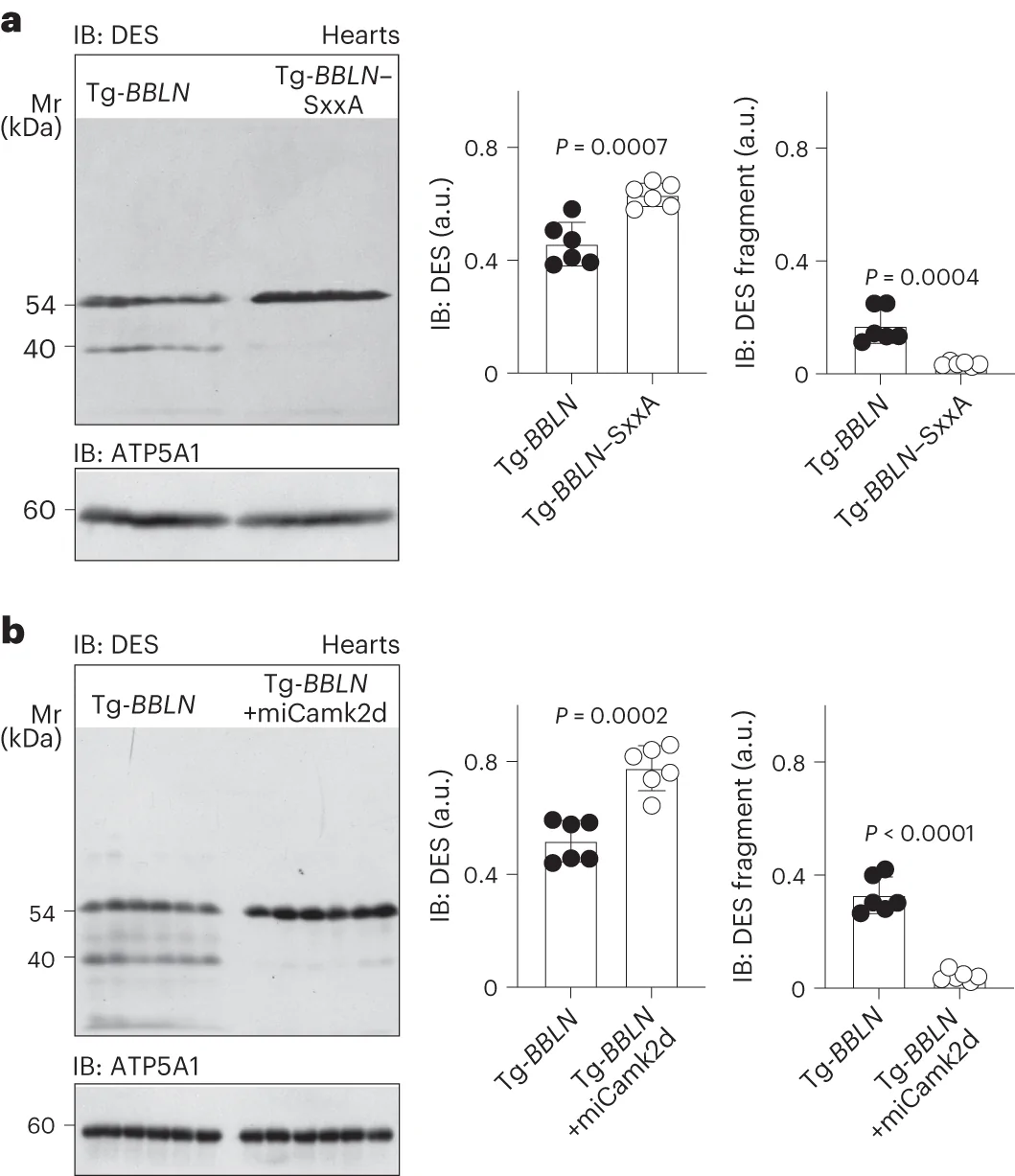
Induction of DES degradation by BBLN. **a**, Cardiac contents of DES were determined by IB analysis of cardiac lysates prepared from male, 8-month-old Tg-*BBLN* (Tg-2) and Tg-*BBLN*–SxxA mice. **b**, IB detection of cardiac DES was performed in male, 8-month-old Tg-*BBLN* mice (Tg-2) without and with lentiviral transduction of an miRNA targeting *Camk2d* by RNAi (+miCamk2d). IB images (left) and quantitative IB data (right) of intact DES and the major DES fragment. Control IBs detect ATP5A1. Data are mean ± s.d. (*n* = 6 mice per group). *P* values were determined by an unpaired, two-tailed *t*-test (d.f. 10; **a**, *t* = 4.870 and 5.281; *P* = 0.0006519 and 0.0003571; **b**, *t* = 5.825 and 10.40; *P* = 0.0001673 and 0.000001111). Source data

Calcium enhances the degradation of the major intermediate filament protein, desmin (DES), in heart failure^40,41^, and BBLN interacts with intermediate filament proteins^42^. Tg-*BBLN* mice with signs of heart failure and calcium accumulation also showed DES fragmentation, in contrast, the DES protein was largely intact in transgenic Tg-*BBLN–SxxA* mice with expression of the inactive BBLN–SxxA mutant (Fig. 7a). BBLN-induced DES fragmentation was related to BBLN-enhanced CAMK2D activation, because downregulation of CAMK2D protein levels by miCamk2d retarded the BBLN-induced DES fragmentation (Fig. 7b). Together, these data provide strong evidence that BBLN causes features of cardiac degeneration by direct activation of CAMK2D.

## Upregulation of BBLN by chronic pressure overload in mice

**Fig. 8:**
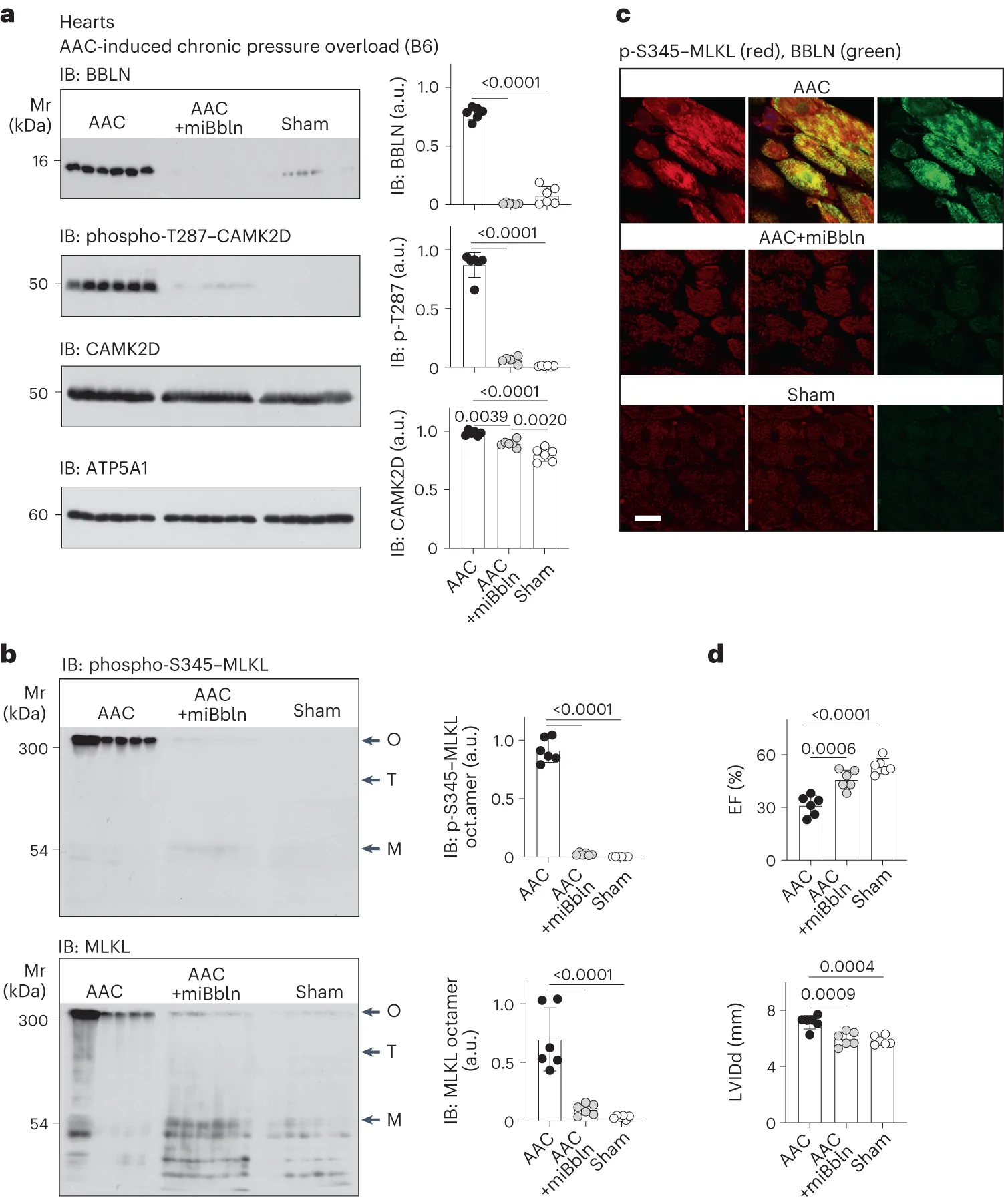
Chronic pressure overload triggered BBLN and features of necroptosis. **a**, Quantitative IB determination of cardiac contents of BBLN and activated phospho-T287–CAMK2D and total CAMK2D in male, 4-month-old B6 mice with 2 months of AAC, 4-month-old B6-miBbln mice with 2 months of AAC (AAC+miBbln) and 4-month-old sham-operated B6 mice (sham). IB images (left) and quantitative IB data (right). The lower control blot detects ATP5A1. **b**, IB determination of cardiac phospho-S345–MLKL (upper) and total MLKL (lower) contents of male, 4-month-old B6 mice with 2 months of AAC (AAC), 4-month-old B6-miBbln mice with 2 months of AAC (AAC+miBbln) and 4-month-old sham-operated B6 mice (sham). Predominant MLKL octamers as a feature of necroptosis were quantified. MLKL octamers (O), tetramers (T) and monomers (M) are marked with arrows. IB images (left) and quantitative IB data (right). **c**, Immunofluorescence colocalization of BBLN with p-S345–MLKL on heart specimens of a 4-month-old B6 mouse with 2 months of AAC (AAC), a 4-month-old B6-miBbln mouse with 2 months of AAC (AAC+miBbln) and a 4-month-old sham-operated B6 mouse (sham). The immunofluorescence is representative of four mouse hearts per group (scale bar, 40 μm). **d**, The left ventricular EF and the LVIDd were determined by echocardiography. Data are mean ± s.d. (*n* = 6 male mice per group; one-way ANOVA and Tukey’s test; **a**, *F*(2,15) = 379.4, 354.4 and 32.97; top: *P* = 0, 1.246 × 10^−12^ and 0.09708; middle: *P* = 1.727 × 10^−12^, 3.11 × 10^−13^ and 0.3463; bottom: *P* = 0.003928, 0.000002048 and 0.001971; **b**, *F*(2,15) = 458.4 and 31.79; top: *P* = 0, 0 and 0.808; bottom: *P* = 0.00002672, 0.00000835 and 0.7711. **d**, *F*(2,15) = 28.47 and 15.68; top: *P* = 0.0005565, 0.000006051 and 0.05481; bottom: *P* = 0.0009046, 0.000402 and 0.9084. Source data

Human BBLN is an *EGR1*-responsive gene, which is upregulated in TOF patient heart specimens with cyanosis (Extended Data Fig. 1a–c and ref. ^15^). However, the *EGR1* response element is not present in the mouse *Bbln* gene (Extended Data Fig. 1a). Nevertheless, previous studies showed that the murine and rat *Bbln* were triggered in different heart failure models (Extended Data Fig. 1e,f). To clarify the role of endogenous *Bbln*, we investigated the pathophysiologic profile of endogenous *Bbln* upregulation in the mouse because the human and murine BBLN amino acid sequences have 94% identity (Extended Data Fig. 1d). Endogenous *Bbln* upregulation was induced in a murine heart failure model, which was triggered by chronic pressure overload imposed by abdominal aortic constriction (AAC) (Fig. 8). Two months of AAC led to a significant increase in cardiac murine BBLN protein contents together with increased activated phospho-T287–CAMK2D levels (Fig. 8a). Concomitantly, the necroptosis effector, that is, the aggregated phospho-S345–MLKL octamer, was triggered (Fig. 8b), and showed colocalization with BBLN on cardiac specimens with necroptosis (Fig. 8c). Downregulation of endogenously expressed *Bbln* by transgenic expression of a *Bbln*-targeting miRNA under control of the ubiquitous cytomegalovirus (CMV) promoter led to decreased cardiac phospho-T287–CAMK2D contents, reduced the levels of aggregated phospho-S345–MLKL octamers as effectors of necroptosis and improved the cardiac function in heart failure induced by AAC (Fig. 8a–d). Thus, chronic pressure overload promoted the upregulation of the endogenous BBLN protein in the mouse. These pathologically elevated endogenous cardiac BBLN protein levels contributed to cardiac dysfunction and necroptotic cardiac remodeling in vivo, in the AAC-induced heart failure model.

The in vivo function of the human BBLN protein, which is encoded by the chromosome 9 open reading frame 16 (C9orf16; ref. ^43^) was largely unknown. This study shows that increased cardiac levels of the human and murine BBLN protein promoted cardiac dysfunction and adverse cardiac remodeling in Tg-*BBLN* mice and in mice with chronic pressure overload-induced cardiac dysfunction. BBLN triggered cardiac damage in a protein dosage-dependent manner by interaction with CAMK2D and enhancement of the activating CAMK2D autophosphorylation on T287 in vitro and in vivo. Phosphorylated phospho-T287–CAMK2D exerts autonomous kinase activity, which is independent of its stimuli, calcium and calmodulin^23^. By CAMK2D activation, BBLN promoted CAMK2D-dependent adverse cardiac remodeling with cardiac dysfunction, fibrosis and necroptosis. Vice versa, BBLN-induced cardiac pathologies and necroptosis were retarded by RNAi-mediated CAMK2D protein downregulation. Furthermore, a BBLN mutant with defective CAMK2D interaction (BBLN–SxxA), which did not act as an activating CAMK2D substrate, was largely inert in vitro and did not stimulate cardiac dysfunction nor negative cardiac remodeling in vivo, in transgenic Tg-*BBLN*–SxxA mice.

In vitro experiments found that BBLN interacts with the CAMK2D kinase domain and acts as an activating CAMK2D substrate. Sequence alignment showed that the human BBLN amino acid sequence displayed major features, which are characteristic for CAMK2 kinase domain interaction and which are conserved in CAMK2-activating substrates^28,29^. Likewise, the interaction of BBLN with the CAMK2D kinase domain was reduced in the presence of the prototypical CAMK2-activating peptide of GRIN2B. In addition to CAMK2D activity enhancement, human BBLN interacts with intermediate filaments^42^. In this respect, human BBLN resembles the *Caenorhabditis elegans* ortholog of BBLN, which also interacts with intermediate filaments^44^. However, the major CAMK2-activating features are apparently missing in the amino acid sequence of the *C.* *elegans* ortholog of BBLN, with only 15.9% identity with the human BBLN amino acid sequence^43,44^. Therefore, the pathological phenotypes of the human BBLN protein and the *C.* *elegans* ortholog could be different.

We found that BBLN was upregulated in human heart specimens of TOF patients with cyanosis. Upregulation of the human BBLN protein could be triggered by hypoxia in TOF patients with cyanosis because the human BBLN gene is an EGR1-responsive gene, and EGR1 was found to be induced in cyanotic TOF patient hearts^15^. *BBLN* expression correlated with markers of adverse cardiac remodeling in right ventricular TOF patient heart specimens with cyanosis and in right ventricular heart tissue of Tg-*BBLN* mice. The increased BBLN levels could contribute to the cardiac deterioration of TOF patients because elevated cardiac BBLN levels caused cardiac remodeling and cardiac dysfunction in vivo, in Tg-*BBLN* mice subjected to pressure overload, in a protein dosage-dependent manner

Taken together, this study identified BBLN as a pathologic factor in TOF patients with hypoxia and in mice overexpressing BBLN under cardiac pressure overload. The upregulated BBLN protein, at least in the mouse model, promotes adverse cardiac remodeling and predisposes to an increased heart failure risk. One of the limitations of the current study is that in vivo work was not done in a mouse model of TOF, but rather in a model of pressure overload as a proxy. As hypoxia is a major pathological feature of TOF patients worldwide, the identification of the elucidated BBLN–CAMK2D pathway may account for a substantial disease burden of many TOF patients worldwide^45^. In addition, BBLN could contribute to the cardiac deterioration of patients with other hypoxia-induced forms of heart disease. Consequently, future treatment modalities of hypoxic conditions in the heart could aim to target pathologic BBLN functions, for example, by RNAi-mediated downregulation of BBLN or by interference with the BBLN-enhanced CAMK2D kinase activation.

## Methods

### Ethical compliance

The research complies with all relevant ethical regulations. Animal experiments were approved by the local committees on animal research (Kantonales Veterinäramt Zurich ZH215/2020, date of approval 15 March 2021; Kantonales Veterinäramt Zurich 145-G, date of approval 14 February 2013; Kantonales Veterinäramt Zürich 126/2009, date of approval 4 August 2009; Medical Research Center (MRC), Ain Shams University Hospital, Cairo, date of approval 2 January 2007). The study protocol analyzing human heart specimens from pediatric TOF patients was performed in compliance with all relevant ethical regulations and was approved by the ethical committee of the MRC, Ain Shams University Hospital, Cairo, Egypt (date of approval 18 October 2006). Informed consent was obtained from all parents. There was no participant compensation.

### Generation of transgenic mice and animal experiments

The following transgenic mouse lines were generated and used: FVB/N-Tg(MHCBBLN) Sjaa (Tg-1), FVB/N-Tg(MHCBBLN)2 Sjaa (Tg-2), FVB/N-Tg(MHCBBLN)3 Sjaa (Tg-*BBLN* low), FVB/N-Tg(MHCBBLN-SxxA) Sjaa and B6-Tg(CMVmiBBLN) Sjaa. Chronic pressure overload was imposed by AAC for 2 months, starting at an age of 8 weeks. Endogenously expressed *Camk2d* was downregulated for 4 weeks by lentiviral transduction of 7-month-old transgenic Tg-*BBLN* mice by intraperitoneal administration of a replication-incompetent lentivirus that expressed a pre-miRNA targeting *Camk2d* by RNAi (5 × 10^8^ copies per mouse). The lentiviral expression plasmid was generated by insertion of double-stranded oligonucleotides into the pLenti6/V5-Dest Gateway Vector (Invitrogen): miCamk2d top strand: 5′-TGCTGTTCAAGAGACGGCAGATTCTAGTTTTGGCCACTGACTGACTAGAATCTCGTCTCTTGAA-3′; miCamk2d bottom strand: 5′- CCTGTTCAAGAGACGAGATTCTAGTCAGTCAGTGGCCAAAACTAGAATCTGCCGTCTCTTGAAC-3′. Transgenic mouse lines have myocardium-specific expression of *BBLN* and mutated *BBLN–SxxA* (S2A, Y28A, S33A, S40A, S62A, T66A, S79A and S82A) under control of the alpha-MHC promoter. Tg-CMVmiBbln mice have ubiquitous expression of miBbln targeting endogenously expressed *Bbln* in mice by RNAi under control of the CMV immediate early promoter/enhancer. The miBbln expression plasmid was generated by insertion of double-stranded oligonucleotides into the BamHI-XhoI restriction sites of the pcDNA6.2 GW plasmid (Invitrogen): miBbln top strand: 5′-TGCTGTGGAGTTGATGGCAGCATACTGTTTTGGCCACTGACTGACAGTATGCTCATCAACTCCA-3′; miBbln bottom strand: 5′-CCTGTGGAGTTGATGAGCATACTGTCAGTCAGTGGCCAAAACAGTATGCTGCCATCAACTCCAC-3′. Identity of PCR-amplified DNA sequences of all plasmids was routinely controlled and confirmed by DNA sequencing. Sperm of transgenic mouse lines were cryopreserved in the JAX repository (FVB/N-Tg(MHCBBLN)2 Sjaa, JAX ID 911828; FVB/N-Tg(MHCBBLN)3 Sjaa, JAX ID 400645; B6-Tg(CMVmiBBLN) Sjaa, JAX ID 913527), and in the Janvier repository (FVB/N-Tg(MHCBBLN) Sjaa, Janvier ID 181.085 ETH Zurich; FVB/N-Tg(MHCBBLN-SxxA) Sjaa, Janvier ID 182.423 ETH Zurich), and are available upon reasonable request.

This study used male mice and female mice, and investigated the phenotype of transgenic mice in comparison to age- and sex-matched nontransgenic mice at an age of 3–4 months and 8 months as indicated. Sample size of animal experiments was predetermined. No randomization was performed. Experiments with transgenic mice were routinely performed with mice from at least three different breeder pairs. At the end of the observation period, mice were anesthetized by intraperitoneal (i.p.) injection of ketamine and medetomidine (75 mg kg^−1^ and 0.5 mg kg^−1^ body weight, respectively), and cardiac function parameters were measured in the parasternal long-axis view by an observer, who was blinded to the genotype by echocardiography in M-mode with a Vivid 7 ultrasound system using a M12L linear array transducer (GE Healthcare). Data evaluation was performed with the EchoPAC software version 3.0 (GE Healthcare), and the left ventricular cardiac EF was determined by the formula of Teichholz. For isolation of RNA and proteins, transcardial perfusion of mice was performed with phosphate-buffered saline (PBS), under terminal anesthesia (ketamine and xylazine, 200 mg kg^−1^ and 60 mg kg^−1^ body weight, respectively, i.p.). Hearts were immediately frozen in liquid nitrogen or processed for histology.

Animal experiments were approved by the local committees on animal research (Kantonales Veterinäramt Zurich ZH215/2020, date of approval 15 March 2021; Kantonales Veterinäramt Zurich 145-G, date of approval 14 February 2013; Kantonales Veterinäramt Zürich 126/2009, date of approval 4 August 2009; MRC, Ain Shams University Hospital, Cairo, date of approval 2 January 2007).

### Human TOF heart specimens

The study analyzed human heart specimens of the RVOT from pediatric patients during clinically indicated primary cardiac repair surgery for TOF. For RNA extraction, cardiac specimens (muscle bundles that are routinely resected from the RVOT as part of surgery) were recovered of 11 pediatric patients with sporadic TOF cardiac defects and cyanosis (repeated oxygen saturation measurements of ≤91.0% on room air, mean 85.3 ± 3.3%, and episodes of cyanotic spells; age 23.2 ± 5.0 months; four males and seven females). For RNA extraction, cardiac specimens were collected in RNAlater (Qiagen GmbH, no. 1017980), immediately frozen and transferred to −80 °C for long-term storage. In addition, cardiac specimens were obtained from other 16 TOF patients with cyanosis (oxygen saturation ≤91.0%, mean 86.7 ± 3.5% and episodes of cyanotic spells; age 24.4 ± 5.7 months; seven males and nine females) and 16 TOF patients without cyanosis (oxygen saturation ≥92%, mean 94.3 ± 1.6% and no episodes of cyanotic spells; age 28.8 ± 6.8 months; eight males and eight females). These 32 cardiac specimens were cut into two pieces and used for histological analysis (collected in 10% formalin), and protein extraction (collected in radioimmunoprecipitation assay (RIPA) buffer supplemented with protease/phosphatase inhibitor cocktail, immediately frozen and transferred to −80 °C for long-term storage).

All pediatric patients had sporadic TOF cardiac defects without any other malformation and had no 22q11 deletion. Informed consent was obtained from all parents. There was no participant compensation. The study protocol was performed in compliance with all relevant ethical regulations and was approved by the ethical committee of the MRC, Ain Shams University Hospital, Cairo, Egypt (date of approval 18 October 2006).

### Cardiac transcriptome profiling by NGS and whole-genome microarray gene expression analysis

For transcriptome analysis by NGS, right and left ventricular mouse heart specimens from 3–4-month-old male Tg-*BBLN* (Tg-1) mice, and age- and sex-matched nontransgenic FVB mice were dissected, pulverized under liquid nitrogen and total RNA was isolated by the RNeasy Midi kit (Qiagen GmbH, cat. no. 75144). For transcriptome sequencing by NGS, RNA libraries were prepared using standard Illumina protocols in frame of the INVIEW Transcriptome Discover protocol (Eurofins Genomics Germany GmbH). The hearts of 8-month-old, male Tg-*BBLN* (Tg-1) mice and nontransgenic FVB controls were similarly processed for NGS. Illumina paired-end read sequencing (2 × 150 bp) with guaranteed 30 million read pairs per sample was done by the Genome Sequencer Illumina HiSeq, in the sequence mode HiSeq 4000. RNA sequencing reads in FASTQ format were imported into the program CLC Genomics workbench 20 version 20.0.4 (Qiagen Bioinformatics) and mapped to the reference genome (Mouse GRCm39) in frame of the RNA Sequencing Workflow of CLC Genomics Workbench 20. NGS transcriptome profiling of right ventricular heart tissue of Tg-*BBLN* mice was validated by the detection of right ventricle-specific transcript changes in comparison to left ventricular tissue (Extended Data Fig. 4). Transcript selection is based on literature data of differential gene expression between right and left ventricles from hypoxic rats, heart failure rats and/or right ventricular heart failure patients^46–48^.

Whole-genome microarray gene expression profiling of 11 human TOF heart specimens was performed with frozen cardiac specimens from 11 pediatric TOF patients with cyanosis (four males and seven females; age 23.2 ± 5.0 months). The synthesis of complementary DNA, labeling and hybridization of fragmented cRNA (15 μg) with the gene chip (Affymetrix GeneChip Human Genome U133 Plus 2.0 Array) were performed according to the Affymetrix protocol (Affymetrix GeneChip Expression Analysis Technical Manual Rev. 5). Microarray gene expression profiling was similarly performed of hearts from 8-month-old, male Tg-*BBLN* (low) mice and compared with those of age- and sex-matched nontransgenic FVB mice using the Mouse Genome MG430 2.0 Array (Affymetrix). The signals were processed using Affymetrix GeneChip Operating Software (v.1.4; Affymetrix). To compare samples and experiments, the trimmed mean signal of each array was scaled to a target intensity of 300.

### IB detection of proteins

For IB detection of proteins, hearts were pulverized under liquid nitrogen, and proteins were extracted with RIPA buffer (150 mM NaCl, 1% NP40, 0.5% sodium deoxycholate, 0.1% sodium dodecyl sulfate (SDS) and 25 mM Tris, pH 7.4) supplemented with a protease/phosphatase inhibitor cocktail. Frozen human heart specimens, which were collected in RIPA buffer (supplemented with protease/phosphatase inhibitors), were thawed and lysed by sonication on ice. Insoluble material was removed by centrifugation followed by delipidation of solubilized cardiac proteins by the addition of acetone/methanol (12:2) at a final concentration of 83% for 90 min at 4 °C. The precipitate was washed with ice-cold acetone and dissolved for 90 min at room temperature in 6 M urea-containing SDS-sample buffer (with 2% SDS, 0.1 M dithiothreitol (DTT) and supplemented with a protease/phosphatase inhibitor cocktail). After the addition of iodoacetamide (10 mM), samples were stored at −80 °C until further use. Proteins were separated by 6 M urea-containing SDS–polyacrylamide gel electrophoresis (PAGE) (7.5% for proteins ≥40 kDa, and 10% for proteins <40 kDa) under reducing conditions. For IB detection, proteins were transferred to polyvinylidene fluoride membranes. After a blocking step, incubation with affinity-purified antibodies or F(ab’)_2_ fragments of the respective antibodies (preabsorbed to mouse/human serum proteins) at a dilution of 1:2,000–1:4,000 in blocking buffer was performed, and unbound primary antibodies were removed by washing steps. Thereafter, incubation with F(ab’)_2_ fragments of affinity-purified peroxidase-coupled secondary antibodies, preabsorbed to mouse/human serum proteins (dilution 1:40,000), or peroxidase-coupled protein A was performed. After additional washing steps, bound antibodies were visualized by enhanced chemiluminescent detection (Amersham ECL Prime, Cytiva RPN2236; Amersham ECL Select, Cytiva RPN2235).

### AP of CAMK2D and CAMK2D-interacting proteins

To identify CAMK2D-interacting proteins, CAMK2D was AP from human heart specimens of TOF patients. The CAMK2D protein co-enrichment study pooled cardiac specimens of ten TOF patients, which were solubilized for 30 min at 4 °C in solubilization buffer (1% sodium deoxycholate, 0.05% SDS and 0.05% Tween 20 in PBS, pH 7.4, supplemented with protease/phosphatase inhibitors). Insoluble material was removed by centrifugation, and the supernatant was diluted 1:5 with PBS (supplemented with protease/phosphatase inhibitors) and applied to the immunoaffinity matrix (affinity-purified anti-CAMK2D antibodies coupled to Affigel 10; 5 mg IgG coupled to 1 ml of Affigel 10, Bio-Rad Laboratories, cat. no. 1536099). Polyclonal anti-CAMK2D antibodies were raised in rabbit against full-length recombinant His_6_-CAMK2D expressed in and purified from baculovirus-infected Sf9 insect cells. After an overnight incubation at 4 °C, unbound proteins were removed by washing steps with PBS (20 column volumes), and bound proteins were rapidly eluted with 0.25 M NH_4_OH and 10% dioxane, pH 11. The pH of the eluate was immediately adjusted to pH 7.4. Eluted proteins were concentrated by centrifugation through Amicon Ultra centrifugal filters (Amicon Ultra-4 Centrifugal Filter Unit, molecular weight cutoff 3 kDa, UFC800308), dissolved in 2× SDS–Laemmli sample buffer and separated by SDS–PAGE under reducing conditions. Immunoaffinity-enriched CAMK2D protein and co-enriched BBLN were identified by IB detection. Co-enriched BBLN was also identified by nano-LC–ESI–MS/MS analysis (see below).

### Identification of BBLN by nano-LC–ESI–MS/MS analysis

After enrichment of CAMK2D by AP from cardiac specimens of TOF patients, co-enriched BBLN was identified by nano-LC–ESI–MS/MS analysis. Isolated proteins were concentrated, dissolved in 2×SDS–Laemmli sample buffer, and separated by SDS–PAGE under reducing conditions. After staining of the SDS-containing polyacrylamide gel by Coomassie brilliant blue, visualized protein bands were cut out and subjected to nano-LC–ESI–MS/MS analysis. The nano-LC–ESI–MS/MS analysis and protein identification were performed by Proteome Factory AG. Data have been deposited to the PRIDE Proteomics Identifications Database (dataset identifier PXD044695).

### Immunohistology and immunofluorescence

Immunohistological detection of BBLN was performed after antigen retrieval on cardiac specimens of TOF patients, and on longitudinal cardiac sections of Tg-*BBLN* mice and nontransgenic FVB mice, with affinity-purified, polyclonal anti-BBLN antibodies. Bound antibodies were visualized by an enzyme substrate reaction (DAB Enhanced Liquid Substrate System, cat. no. D3939, Sigma-Aldrich). Myocardial calcium accumulation as an indicator of necroptosis/necrosis was determined on cardiac specimens by the von Kossa calcium staining method (Diagnostic BioSystems, cat. no. KT028). Collagen staining of heart specimens by picrosirius red was performed by the Picrosirius Red Stain Kit (ab150681, Abcam). Immunohistological sections were imaged with a DMI6000 microscope equipped with a DFC 420 camera, and an MZ125 stereomicroscope equipped with a DFC 295 camera (Leica Microsystems). Colocalization studies of BBLN with CAMK2D, and of BBLN with phospho-S345–MLKL were performed by immunofluorescence applying (1) rabbit polyclonal anti-BBLN antibodies and mouse monoclonal anti-CAMK2D antibody (WH0000817M2; Sigma-Aldrich), and (2) rabbit polyclonal anti-BBLN antibodies and mouse monoclonal anti-phospho-S345–MLKL antibody (MABC1158, Clone 7C6.1; EMD Millipore Corporation) (dilution 1:200). Secondary antibodies were labeled with Alexa Fluor 488 and Alexa Fluor 568, respectively (dilution 1:4,000). Nuclei were stained with 4,6-diamidino-2-phenylindole. Sections were imaged with a confocal laser scanning microscope (Leica TCS SPE, and Leica SP8 Falcon; Center for Microscopy and Image Analysis at the University of Zurich).

### Antibodies

The following antibodies were used for IB detection, immunohistochemistry and immunofluorescence: rabbit monoclonal anti-ATP2A2/SERCA2 antibody (9580; D51B11; Cell Signaling Technology); rabbit polyclonal anti-C9orf16 (anti-BBLN) antibodies (HPA020725; Prestige Antibodies; Sigma Life Sciences); rabbit polyclonal anti-CAMK2D antibodies (H00000817-DO1P; Abnova); mouse monoclonal anti-CAMK2D antibody, clone 1A8 (WH0000817M2; Sigma-Aldrich); rabbit monoclonal anti-CAMK2D antibody [EPR13095] (ab181052; abcam); rabbit polyclonal anti-phospho-Thr287 CAMKII (beta, gamma, delta) antibodies (PA5-37833; Invitrogen by ThermoFisher Scientific); rabbit monoclonal anti-phospho-Thr286/287 CAMK2 (alpha, beta, gamma, delta) antibody (D21E4; 12716; Cell Signaling Technology); rabbit polyclonal anti-phospho-Thr305 CAMK2 (alpha, beta, gamma, delta) antibodies (Thr307 in mouse CAMK2D) (Abnova PAB29254; B1SA01040G00470); rabbit monoclonal anti-Desmin antibody [Y66] (ab32362, abcam); mouse monoclonal anti-ATP5A antibody [15H4C4] (ab14748; abcam); rabbit monoclonal anti-MLKL antibody (D6W1K) (mouse specific; 37705; Cell Signaling Technology); rabbit monoclonal anti-phospho-S345–MLKL antibody [EPR9515 (ref. ^2^)] (ab 196436; abcam); rat monoclonal anti-MLKL antibody [3H1] (ab243142; abcam); mouse monoclonal anti-phospho-S345-MLKL antibody (MABC1158, Clone 7C6.1; EMD Millipore Corporation); rabbit monoclonal anti-phospho-S358-MLKL (D6H3V) antibody (mAb No. 91689; Cell Signaling Technology); rabbit monoclonal anti-MLKL (D2I6N) antibody (mAb No. 14993 Cell Signaling Technology); rabbit polyclonal anti-RYR2 antibodies (Invitrogen PA5-77717; ThermoFisher Scientific); rabbit polyclonal anti-phospho-Ser2814 RYR2 antibodies (CABP0624; AssayGenie); mouse monoclonal anti-α-Tubulin antibody, clone DM1A (T6199; Sigma); peroxidase-conjugated AffiniPure F(ab’)2 Fragment Goat Anti-Mouse IgG Fcγ Fragment Specific (115-036-071; Jackson ImmunoResearch Laboratories); peroxidase-conjugated AffiniPure F(ab’)2 Fragment Goat Anti-Rabbit IgG, Fc Fragment-Specific (111-036-046; Jackson ImmunoResearch Laboratories); Protein A, Peroxidase Conjugate (539253-1MG; EMD Millipore Corporation); goat anti-Rabbit IgG (H + L) Highly Cross-Adsorbed Secondary Antibody, Alexa Fluor 488 (A11034; Invitrogen by ThermoFisher Scientific); goat anti-Mouse IgG (H + L) Cross-Adsorbed Secondary Antibody, Alexa Fluor 568 (A11004; Invitrogen by ThermoFisher Scientific).

The primary antibody dilution was 1:2,000–1:4,000 for IB detection, and 1:200 for immunohistochemistry and immunofluorescence. Secondary antibody dilution was 1:40,000 for IB detection, 1:500 for immunohistochemistry and 1:4,000 for immunofluorescence.

### Expression and purification of recombinant proteins

Recombinant, hexahistidine-tagged His_6_-CAMK2D was expressed in *Spodoptera frugiperda* (Sf9) cells. Proteins were extracted with lysis buffer (300 mM NaCl, 50 mM HEPES, pH 7.5 supplemented with 1% NP40, 1 mM phenylmethylsulfonyl fluoride and protease inhibitor cocktail) and purified with Ni-NTA affinity chromatography. His_6_-BBLN, His_6_-BBLN–SxxA (BBLN–S2A, Y28A, S33A, S40A, S62A, T66A, S79A and S82A), His_6_-BBLN–Mut1 (BBLN–S2A, T66A, S79A and S82A), His_6_-BBLN–Mut2 (BBLN–Y28A, S33A and S40A), His_6_-PDC and Flag-His_6_-CAMK2D residues 8–275 (inactive kinase domain D136N mutant)^28^ were expressed with the pET-3d expression system (Novagen, no. 69421). Proteins were extracted from bacterial pellets with lysis buffer (300 mM NaCl, 50 mM HEPES, 10 mM imidazole, 10 mM 2-mercaptoethanol and 8 M urea, pH 7.5), and purified by Ni-NTA affinity chromatography. After washing and protein renaturation steps with buffer (300 mM NaCl, 50 mM HEPES and 20 mM imidazole, pH 7.5, 10 mM 2-mercaptoethanol) supplemented with decreasing concentrations of urea (4 M, 2 M and 0 M), bound proteins were eluted with elution buffer (300 mM NaCl, 50 mM HEPES and 500 mM imidazole, pH 7.5, 10 mM 2-mercaptoethanol). After buffer exchange and a protein concentration step, proteins were used for the in vitro phosphorylation assay.

### In vitro phosphorylation and binding assays

In vitro phosphorylation of PDC, and autophosphorylation of CAMK2D were performed in an assay volume of 50 μl by incubation of purified, recombinant His_6_-CAMK2D (50 nM or 200 nM, as indicated) with or without 600 nM His_6_-PDC in reaction buffer (20 mM Tris–HCl, pH 7.5) supplemented with 2.5 μM calmodulin, 4 mM CaCl_2_ and 2 mM ethylenediaminetetraacetic acid and 5 mM MgCl_2_ in the presence of increasing concentrations of purified recombinant His_6_-BBLN (0–30 μM), His_6_-BBLN–SxxA, His_6_-BBLN–Mut1, His_6_-BBLN–Mut2 (30 μM) or control protein (bovine serum albumin). As indicated, Ca^2+^+calmodulin was omitted. The phosphorylation reaction was started by the addition of 50 μM or 100 μM ATP supplemented with [γ-^32^P]-ATP (1 × 10^6^ disintegrations per minute, specific activity 3,000 Ci mmol^−1^, PerkinElmer). After an incubation for 10 min at 30 °C, the reaction was stopped by the addition of 5× SDS–Laemmli buffer. After thermal denaturation, proteins were separated by SDS–PAGE under reducing conditions, and phosphorylated proteins were visualized and quantified by autoradiography or by IB detection of phospho-T287–CAMK2D.

The binding of BBLN, BBLN–Mut1 and BBLN–Mut2 to Flag-His_6_-CAMK2D (kinase domain residues 8–275, D136N mutant)^28^ was determined by a binding assay. Purified Flag-His_6_-CAMK2D kinase domain (residues 8–275, D136N) (1 μM) was immobilized on anti-FLAG-antibody affinity agarose (20 μl beads) by incubation for 2–4 h, at 4 °C, in incubation buffer (25 mM Tris–HCl, 150 mM NaCl, 0.1% bovine serum albumin and 0.01% NP40, pH 7.4) followed by the addition of 1 μM BBLN protein in the absence or presence of 30 μM peptide (GRIN2B 1289–1310: KAQKKNRNKLRRQHSYDTFVDL; control peptide GRIN2B 1095–1119: SAKSRREFDEIELAYRRRPPRSPDH)^28,29^, and incubation for 4 h, at 4 °C. After three washing steps with incubation buffer, elution was performed with SDS–PAGE sample buffer. Eluted proteins were visualized and quantitated after SDS–PAGE by IB detection.

### Statistical analyses and software

The presented data show biological replicates unless otherwise stated. All the nonhuman data of this study have been confirmed at least three times with consistent results. Applied statistical tests are indicated in the figure legends, and data are presented as mean ± s.d. Comparisons between two groups were made with the unpaired, two-tailed *t*-test, and for comparisons between more than two groups, ordinary one-way analysis of variance (ANOVA) with a post-test as indicated was used. Survival analyses were performed by Kaplan–Meier survival analysis with a log-rank (Mantel–Cox) test. Linear regression analysis was performed for linear dependence analysis between two variables, and the Pearson correlation (*r*) was determined. A p-value of <0.05 was considered as statistically significant. Only transcripts showing correlation with *BBLN* in human RVOT specimens of cyanotic TOF patients with a *P* value of <0.05 and a Pearson correlation (*r*) of ≥+0.6 or ≤−0.6 were included in the overrepresentation analysis. Statistical analyses were performed with R, and the linear regression analysis was performed with Excel 2019 (version 16.0). GraphPad Prism (version 9.3.1) was used for creation of graphs. NGS transcriptome data comparisons between two groups were performed by MeV, and the unpaired, two-tailed *t*-test (just alpha)^49^. The heat map was generated by Morpheus (https://software.broadinstitute.org/morpheus). The overrepresentation analysis was performed with g:GOSt of g:Profiler (version e107_eg54_p17_bf42210, database updated on 15.09.2022). *P* values were determined with Fisher’s one-tailed test^50^. Multiple testing correction was performed with the G:SCS algorithm of g:GOSt^50^. Multiple sequence alignment was performed with the Clustal Omega (CLUSTAL O, version 1.2.4) tool from European Molecular Biology Laboratory’s European Bioinformatics Institute^51^. Pairwise sequence alignment was performed with EMBOSS Needle. A search of the National Center for Biotechnology Information GEO dataset browser identified upregulation of *BBLN*-*Bbln* in GEO datasets GDS2008 (GSE2299, ref. ^52^), GDS5302 (GSE54372, ref. ^53^) and GDS3018 (GSE4286, ref. ^54^).

### Data analysis

All NGS data analyses were performed with CLC Genomics Workbench 20 version 20.0.4 (Qiagen Bioinformatics) and mapped to the reference genome (Mouse GRCm39) in frame of the standard RNA Sequencing Workflow of CLC Genomics workbench 20. The following mapping settings were used: mismatch count: 2; insertion cost: 3; deletion cost: 3; length fraction: 0.8; similarity fraction: 0.8; maximum number of hits for a read: 10; expression value: transcripts per million (TPM).

### Reporting summary

Further information on research design is available in the Nature Portfolio Reporting Summary linked to this article.

## Supplementary information

Supplementary Fig.1

Reporting Summary

Supplementary Dataset 1 Overrepresentation analysis of genes with positive correlation with BBLN in TOF patient hearts and upregulation in right ventricles of Tg-BBLN mouse hearts.

Supplementary Dataset 2 Overrepresentation analysis of genes with negative correlation with BBLN in TOF patient hearts and downregulation in right ventricles of Tg-BBLN mouse hearts.

## Source data

Source Data Fig. 1 Statistical source data.

Source Data Fig. 1 Unprocessed western blots.

Source Data Fig. 2 Statistical source data.

Source Data Fig. 2 Unprocessed western blots.

Source Data Fig. 3 Statistical source data.

Source Data Fig. 4 Statistical source data.

Source Data Fig. 4 Unprocessed western blots.

Source Data Fig. 5 Statistical source data.

Source Data Fig. 6 Statistical source data.

Source Data Fig. 6 Unprocessed western blots.

Source Data Fig. 7 Statistical source data.

Source Data Fig. 7 Unprocessed western blots.

Source Data Fig. 8 Statistical source data.

Source Data Fig. 8 Unprocessed western blots.

Source Data Extended Data Fig. 1 Statistical source data.

Source Data Extended Data Fig. 2 Statistical source data.

Source Data Extended Data Fig. 3 Statistical source data.

Source Data Extended Data Fig. 4 Statistical source data.

Source Data Extended Data Fig. 5 Statistical source data.

Source Data Extended Data Fig. 6 Statistical source data.

Source Data Extended Data Fig. 6 Unprocessed western blots.

Source Data Extended Data Fig. 7 Statistical source data.

Source Data Extended Data Fig. 7 Unprocessed western blots.

Source Data Extended Data Fig. 9 Statistical source data.

Source Data Extended Data Fig. 9 Unprocessed western blots.

Source Data Extended Data Fig. 10 Statistical source data.

Source Data Extended Data Fig. 10 Unprocessed western blots.

## Data Availability

NGS data and whole-genome microarray gene expression data that support the findings of the study have been deposited in the National Center for Biotechnology Information GEO database and are accessible through GEO Series accession numbers GSE241022, GSE241024, GSE241030 and GSE241161. MS data of BBLN identification by nano-LC–ESI–MS/MS analysis have been deposited to PRIDE Proteomics Identifications Database (dataset identifier PXD044695). Immunohistology and immunofluorescence image source files have been deposited in the online repository Eidgenoessische Technische Hochschule Zurich Research Collection (https://doi.org/10.3929/ethz-b-000630673). All other data analyzed during this study are included in the main article and associated files. Source data are provided with this paper.
