# Supplementary material for: BBLN triggers CAMK2D pathology in mice under cardiac pressure overload and potentially in unrepaired hearts with tetralogy of Fallot
Source: Nat Cardiovasc Res. 2023 Oct 26;2(11):1044–59. doi: 10.1038/s44161-023-00351-6 (PMC11041739; doi:10.1038/s44161-023-00351-6)
Supplement: Supplementary file 1 — Supplementary Fig.1 [file 44161_2023_351_MOESM1_ESM.pdf]

nature cardiovascular research

Article https://doi.org/10.1038/s44161-023-00351-6

# BBLN triggers CAMK2D pathology in mice under cardiac pressure overload and potentially in unrepaired hearts with tetralogy of Fallot

In the format provided by the authors and unedited

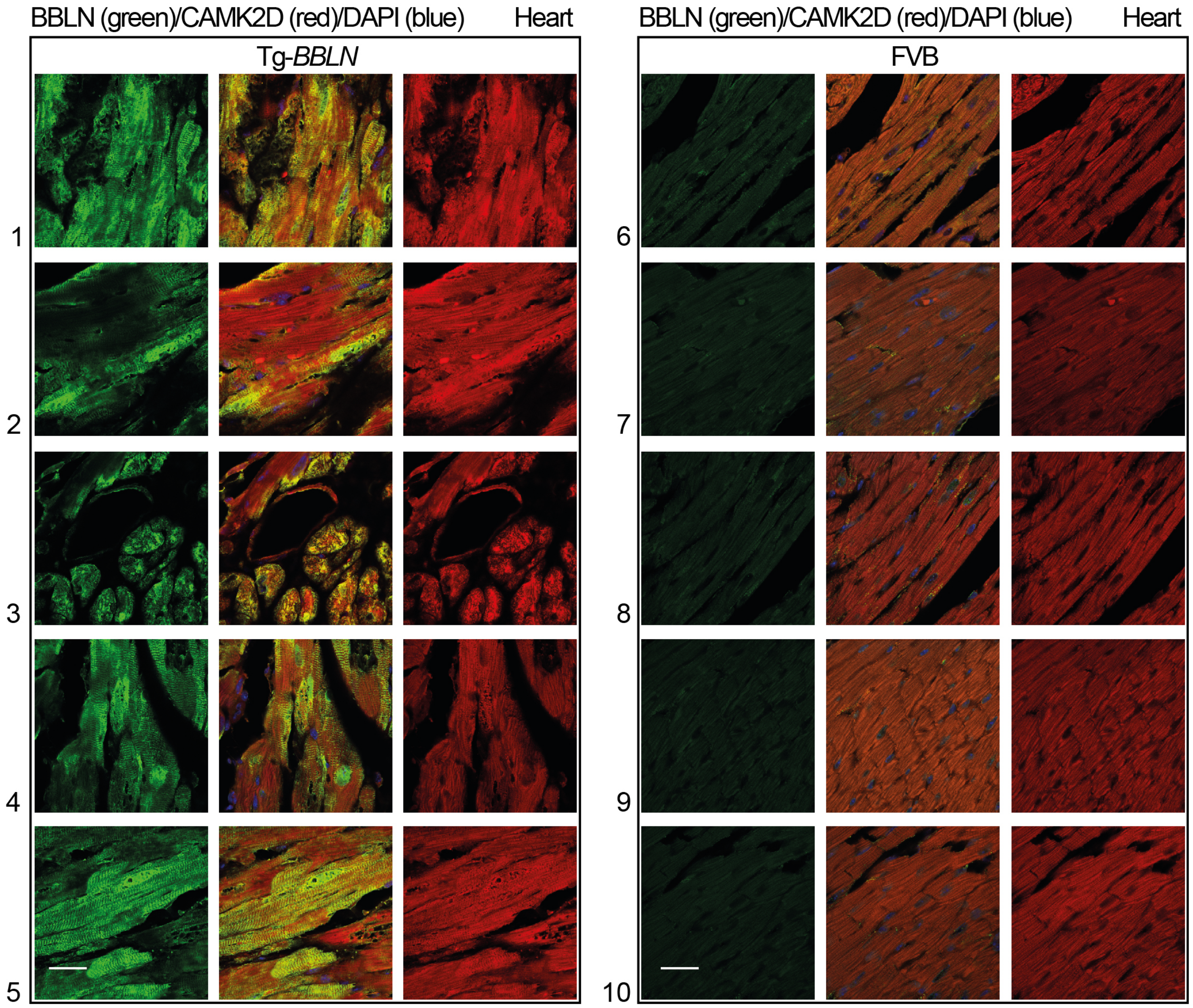


**Supplementary Figure 1 (related to Figure 4a).** BBLN is co-localized with CAMK2D in transgenic Tg-*BBLN* (Tg-1) mouse hearts. Immunofluorescence co-localization of BBLN with CAMK2D on heart specimens of 8-month-old, male Tg-*BBLN* mice (left panels, no. 1-5). Controls are 8-month-old, male non-transgenic FVB mice (right panels, no. 6-10); bar: 40 μm; n=5 mice per group.
