# Supplementary material for: BBLN triggers CAMK2D pathology in mice under cardiac pressure overload and potentially in unrepaired hearts with tetralogy of Fallot
Source: Nat Cardiovasc Res. 2023 Oct 26;2(11):1044–59. doi: 10.1038/s44161-023-00351-6 (PMC11041739; doi:10.1038/s44161-023-00351-6)
Supplement: Supplementary file 2 — Reporting Summary [file 44161_2023_351_MOESM2_ESM.pdf]

nature portfolio

Corresponding author(s): Ursula Quitterer

Last updated by author(s): Sep 12, 2023

# Reporting Summary

Nature Portfolio wishes to improve the reproducibility of the work that we publish. This form provides structure for consistency and transparency in reporting. For further information on Nature Portfolio policies, see our Editorial Policies and the Editorial Policy Checklist.

## Statistics

For all statistical analyses, confirm that the following items are present in the figure legend, table legend, main text, or Methods section.

| n/a | Confirmed | |
|---|---|---|
| ☐ | ☒ | The exact sample size (*n*) for each experimental group/condition, given as a discrete number and unit of measurement |
| ☐ | ☒ | A statement on whether measurements were taken from distinct samples or whether the same sample was measured repeatedly |
| ☐ | ☒ | The statistical test(s) used AND whether they are one- or two-sided<br>*Only common tests should be described solely by name; describe more complex techniques in the Methods section.* |
| ☒ | ☐ | A description of all covariates tested |
| ☐ | ☒ | A description of any assumptions or corrections, such as tests of normality and adjustment for multiple comparisons |
| ☐ | ☒ | A full description of the statistical parameters including central tendency (e.g. means) or other basic estimates (e.g. regression coefficient) AND variation (e.g. standard deviation) or associated estimates of uncertainty (e.g. confidence intervals) |
| ☐ | ☒ | For null hypothesis testing, the test statistic (e.g. *F*, *t*, *r*) with confidence intervals, effect sizes, degrees of freedom and *P* value noted<br>*Give P values as exact values whenever suitable.* |
| ☒ | ☐ | For Bayesian analysis, information on the choice of priors and Markov chain Monte Carlo settings |
| ☒ | ☐ | For hierarchical and complex designs, identification of the appropriate level for tests and full reporting of outcomes |
| ☐ | ☒ | Estimates of effect sizes (e.g. Cohen's *d*, Pearson's *r*), indicating how they were calculated |

*Our web collection on statistics for biologists contains articles on many of the points above.*

## Software and code

Policy information about availability of computer code

| | |
|---|---|
| Data collection | A Vivid 7 echocardiograph equipment with the EchoPAC PC 3.0 Software was used for echocardiography data collection and analysis. Immunohistology sections were imaged with a DMI6000 microscope equipped with a DFC 420 camera (Leica Microsystems, Wetzlar, Germany), and an MZ125 stereomicroscope equipped with a DFC 295 camera (Leica Microsystems, Wetzlar, Germany). A Leica TCS SPE microscope and a Leica SP8 Falcon microscope were used for the confocal microscopy image data collection. |
| Data analysis | GraphPad Prism (Version 9.3.1) was used for creation of graphs. NGS data comparisons between two groups were performed by MeV, which used the unpaired, two-tailed t-test (just alpha). Statistical analyses were performed with R, and the linear regression analysis was performed with Microsoft Excel (version 16.58). The signals of microarray gene expression chips were processed using Affymetrix GeneChip Operating Software (GCOS; v.1.4; Affymetrix). All NGS data analyses were performed with CLC Genomics workbench 20 version 20.0.4 (QIAGEN Bioinformatics, QIAGEN Aarhus A/S) and mapped to the reference genome (Mouse GRCm39) in frame of the standard RNA Sequencing Workflow of CLC Genomics workbench 20. The following mapping settings were used: Mismatch count: 2; Insertion cost: 3; Deletion cost: 3; Length fraction: 0.8; Similarity fraction: 0.8; Maximum number of hits for a read: 10; Expression value: TPM. No new code was generated. Protein identification from the nanoHPLC-ESI-MS/MS analyses was performed by MS/MS ion search with the Mascot search engine. The heat map was generated by Morpheus (https://software.broadinstitute.org/morpheus). The over-representation analysis was performed with g:GOsSt of g:Profiler (version e107_eg54_p17_bf42210, database updated on 15/09/2022). The over-representation analysis determined the statistically significant enrichment by the cumulative hypergeometric probability test. Adjusted P-values (-log10) were determined measuring the randomness of the intersection between the query and the indicated Reactome pathway. The statistical domain scope was set to “all known genes” (effective domain size: 62649), and the significance threshold (g:SCS threshold) was set to 0.05. As data source the Reactome pathway database was used. P-values were determined by g:GOSt of g:Profiler with Fisher's one-tailed test. Multiple testing correction was performed with the G:SCS algorithm of g:GOSt. Multiple sequence alignment was performed with the Clustal Omega (CLUSTAL O, version |

1.2.4) tool from EMBL-EBI. The NCBI GEO Database was used to identify GEO datasets with up-regulation of the human BBLN gene, the murine Bbln gene and the rat Bbln gene.

For manuscripts utilizing custom algorithms or software that are central to the research but not yet described in published literature, software must be made available to editors and reviewers. We strongly encourage code deposition in a community repository (e.g. GitHub). See the Nature Portfolio guidelines for submitting code & software for further information.

## Data

Policy information about availability of data

All manuscripts must include a data availability statement. This statement should provide the following information, where applicable:

- Accession codes, unique identifiers, or web links for publicly available datasets
- A description of any restrictions on data availability
- For clinical datasets or third party data, please ensure that the statement adheres to our policy

NGS data, and whole genome microarray gene expression data that support the findings of the study have been deposited in the NCBI GEO database and are accessible through GEO Series accession numbers GSE241022, GSE241024, GSE241030, and GSE241161. MS data of BBLN identification by nano-LC-ESI-MS/MS analysis have been deposited to the PRIDE Proteomics Identifications Database (dataset identifier PXD044695). Immunohistology and immunofluorescence image source files have been deposited in the online repository ETH Research Collection (doi: 10.3929/ethz-b-000630673). All other data analyzed during this study are included in the main article and associated files. Source data are provided with this manuscript.

## Research involving human participants, their data, or biological material

Policy information about studies with human participants or human data. See also policy information about sex, gender (identity/presentation), and sexual orientation and race, ethnicity and racism.

**Reporting on sex and gender**

For RNA extraction, cardiac specimens were recovered of 11 paediatric patients with sporadic TOF cardiac defects and cyanosis (4 male, 7 female; age 23.2±5.0 months). Sex of these 11 paediatric patients was confirmed by whole genome microarray gene expression data. In addition, cardiac specimens (muscle bundles that are routinely resected from the RV outflow tract as part of surgery) were obtained from other 16 TOF patients with cyanosis (7 male, 9 female; age 24.4±5.7 months) and of 16 TOF patients without cyanosis (8 male, 8 female; age 28.8±6.8 months).

**Reporting on race, ethnicity, or other socially relevant groupings**

The manuscript did not report on race, ethnicity, or other social grouping.

**Population characteristics**

The study analysed human heart specimens of the right ventricular outflow tract (RVOT), which were recovered from pediatric patients during clinically indicated primary cardiac repair surgery for TOF at the Ain Shams University Hospital, Cairo, Egypt. For RNA extraction, cardiac specimens (muscle bundles that are routinely resected from the RV outflow tract as part of surgery) were recovered of 11 pediatric patients with sporadic TOF cardiac defects and cyanosis (repeated oxygen saturation measurements of ≤91.0% on room air, mean 85.3 ± 3.3 %, and episodes of cyanotic spells; age 23.2±5.0 months; 4 male, 7 female). In addition, cardiac specimens (muscle bundles that are routinely resected from the RV outflow tract as part of surgery) were obtained from other 16 TOF patients with cyanosis (oxygen saturation ≤ 91.0 %, mean 86.7±3.5% and episodes of cyanotic spells; age 24.4±5.7 months; 7 male, 9 female) and from 16 TOF patients without cyanosis (oxygen saturation ≥ 91.3 %, mean 94.2±1.7% and no episodes of cyanotic spells; age 28.8±6.8 months; 8 male, 8 female). All pediatric patients had sporadic TOF cardiac defects without any other malformation and no 22q11 deletion.

**Recruitment**

The study analysed human heart specimens of the right ventricular outflow tract (RVOT), which were recovered from pediatric patients during clinically indicated cardiac repair surgery for TOF, at the Ain Shams University Hospital, Cairo, Egypt. Informed consent was obtained from all parents. There was no participant compensation.

**Ethics oversight**

The study protocol analyzing human heart specimens from pediatric TOF patients was performed in compliance with all relevant ethical regulations and approved by the ethical committee of the Medical Research Center (MRC), Ain Shams University Hospital, Cairo, Egypt (date of approval 18.10.2006). Informed consent was obtained from all parents. There was no participant compensation.

Note that full information on the approval of the study protocol must also be provided in the manuscript.

# Field-specific reporting

Please select the one below that is the best fit for your research. If you are not sure, read the appropriate sections before making your selection.

☒ Life sciences ☐ Behavioural & social sciences ☐ Ecological, evolutionary & environmental sciences

For a reference copy of the document with all sections, see nature.com/documents/nr-reporting-summary-flat.pdf

# Life sciences study design

All studies must disclose on these points even when the disclosure is negative.

**Sample size**

Sample size of animal experiments was predetermined and based on the expected effect size, which was observed during initial mouse

| | |
|---|---|
| Sample size | phenotyping studies. Sample size determination was performed in frame of the statistical pre-evaluation of the study by Novustat GmbH (Wollerau, Switzerland). Sample size determination defined the minimum number of animals allowing statistical analysis while considering the 3Rs rule on reduction, refinment and replacement of animal use for scientific experiments. |
| Data exclusions | Data from mice with visual malformations independent from the mouse genotype, as determined by an independent veterinarian, were excluded. In addition, data from mice with (healed) bite wounds were also excluded. |
| Replication | All the non-human data of this study were confirmed at least three times in independent experiments, i.e., with offspring from at least three different pairs of breeders. |
| Randomization | Study groups of mice were matched by age, sex and genetic background, as indicated. Mice were not randomized but all experiments were performed with mice from at least three different pairs of breeders. |
| Blinding | Echocardiography measurements, histology analyses, confocal imaging experiments and protein detection by immunoblot were performed by investigators who were blinded to the genotype. In addition, data analyses of these experiments were performed in a blinded manner. Cardiac transcriptome analysis by NGS of Tg-BBLN and non-transgenic FVB mice was determined by GATC Biotech, an EUROFINS company, without knowing the genotype. Similarly, whole genome microarray gene expression profiling was performed by CORE Facility researchers, who did not know the genotypes and/or study groups. Protein identification by nano-LC-ESI-MS/MS analysis was performed by members of Proteome Factory AG (Berlin, Germany), who did not know the study groups. |

# Reporting for specific materials, systems and methods

We require information from authors about some types of materials, experimental systems and methods used in many studies. Here, indicate whether each material, system or method listed is relevant to your study. If you are not sure if a list item applies to your research, read the appropriate section before selecting a response.

## Materials & experimental systems

| n/a | Involved in the study |
|---|---|
| ☐ | ☒ Antibodies |
| ☒ | ☐ Eukaryotic cell lines |
| ☒ | ☐ Palaeontology and archaeology |
| ☐ | ☒ Animals and other organisms |
| ☒ | ☐ Clinical data |
| ☒ | ☐ Dual use research of concern |
| ☒ | ☐ Plants |

## Methods

| n/a | Involved in the study |
|---|---|
| ☒ | ☐ ChIP-seq |
| ☒ | ☐ Flow cytometry |
| ☒ | ☐ MRI-based neuroimaging |

## Antibodies

| | |
|---|---|
| Antibodies used | The following antibodies were used for immunoblot detection, immunohistochemistry and immunofluorescence.<br>Primary antibody dilution was 1:2000-1:4000 for immunoblot detection, and 1:200 for immunohistochemistry and immunofluorescence. Secondary antibody dilution was 1:40,000 for immunoblot detection, 1:500 for immunohistochemistry, and 1:4000 for immunofluorescence.<br>rabbit monoclonal anti-ATP2A2/SERCA2 antibody (9580; D51B11; Cell Signaling Technology)<br>rabbit polyclonal anti-C9orf16 (anti-BBLN) antibodies (HPA020725, Prestige Antibodies, Sigma Life Sciences);<br>rabbit polyclonal anti-CAMK2D antibodies (H00000817-DO1P; Abnova);<br>mouse monoclonal anti-CAMK2D antibody, clone 1A8 (WH0000817M2; Sigma-Aldrich);<br>rabbit monoclonal anti-CAMK2D antibody [EPR13095] (ab181052, abcam);<br>rabbit polyclonal anti-phospho-Thr287 CAMKII (beta, gamma, delta) antibodies (PA5-37833; Invitrogen, ThermoFisher Scientific);<br>rabbit monoclonal anti-phospho-Thr286/287-CAMK2 (alpha, beta, gamma, delta) antibody (D21E4, 12716, Cell Signaling Technology);<br>rabbit polyclonal anti-phospho-Thr305-CAMK2 (alpha, beta, gamma, delta) antibodies (Thr307 in mouse CAMK2D) (Abnova PAB29254; B1SA01040G00470);<br>rabbit monoclonal anti-Desmin antibody [Y66] (ab32362, abcam);<br>mouse monoclonal anti-ATP5A antibody [15H4C4] (ab14748, abcam);<br>rabbit monoclonal anti-MLKL antibody (D6W1K) (mouse specific; 37705, Cell Signaling Technology);<br>rabbit monoclonal anti-phospho-S345-MLKL antibody [EPR9515(2)] (ab 196436; abcam);<br>rat monoclonal anti-MLKL antibody [3H1] (ab243142; abcam);<br>mouse monoclonal anti-phospho-S345-MLKL antibody (MABC1158, Clone 7C6.1, EMD Millipore Corporation);<br>rabbit monoclonal anti-phospho-S358-MLKL (D6H3V) (mAb No. 91689, Cell Signaling Technology);<br>rabbit monoclonal anti-MLKL (D2I6N) (mAb No. 14993; Cell Signaling Technology);<br>rabbit polyclonal anti-RYR2 antibodies (Invitrogen PA5-77717; ThermoFisher Scientific);<br>rabbit polyclonal anti-phospho-Ser2814-RYR2 antibodies (CABP0624; AssayGenie);<br>mouse monoclonal anti-alpha-Tubulin antibody, clone DM1A (T6199; Sigma);<br>peroxidase-conjugated AffiniPure F(ab')2 Fragment Goat Anti-Mouse IgG Fc gamma Fragment Specific (minimal crossreaction to human, bovine and horse serum proteins) (115-036-071, Jackson ImmunoResearch Laboratories Inc.);<br>peroxidase-conjugated AffiniPure F(ab')2 Fragment Goat Anti-Rabbit IgG, Fc Fragment-Specific (minimal cross-reaction to human serum proteins) (111-036-046; Jackson ImmunoResearch Laboratories Inc.);<br>protein A, Peroxidase Conjugate (539253-1MG; EMD Millipore Corp.);<br>goat anti-Rabbit IgG (H+L) Highly Cross-Adsorbed Secondary Antibody, Alexa Fluor 488 (A11034; Invitrogen by ThermoFisher |

Scientific);
goat anti-Mouse IgG (H+L) Cross-Adsorbed Secondary Antibody, Alexa Fluor 568 (A11004; Invitrogen by ThermoFisher Scientific).

Validation

rabbit monoclonal anti-ATP2A2/SERCA2 antibody (9580; D51B11; Cell Signaling Technology; Lot 3); https://www.cellsignal.com/products/primary-antibodies/atp2a2-serca2-d51b11-rabbit-mab/9580.

rabbit polyclonal anti-C9orf16 (anti-BBLN) antibodies (HPA020725; Prestige Antibodies; Sigma Life Sciences; Lot R10107; Lot A105533); https://www.sigmaaldrich.com/CH/de/product/sigma/hpa020725; validated in BBLN-transfected HEK cells vs. non-transfected cells, and in Tg-BBLN transgenic mice vs. non-transgenic mice (this work).

rabbit polyclonal anti-CAMK2D antibodies (H00000817-DO1P; Abnova; Lot 08289WUIz); https://www.abnova.com/products/products_detail.asp?catalog_id=H00000817-D01P; validated in CAMK2D-transfected HEK cell line vs. non-transfected cells.

mouse monoclonal anti-CAMK2D antibody, clone 1A8 (WH0000817M2; Sigma-Aldrich; Lot G6161-1A8); https://www.sigmaaldrich.com/CH/de/product/sigma/wh0000817m2; validated in CAMK2D-transfected HEK cells vs. non-transfected cells; and enhanced validation by RNAi.

rabbit monoclonal anti-CAMK2D antibody [EPR13095] (ab181052; abcam; Lot GR3279442-1); https://www.abcam.com/camkii-delta-antibody-epr13095-ab181052.html; validated with CAMK2D-knockout HEK-293T cell lysate vs. wild-type HEK-293T cell lysate.

rabbit polyclonal anti-phospho-Thr287 CAMKII (beta, gamma, delta) antibodies (PA5-37833; Invitrogen, ThermoFisher Scientific; Lot SK2476684; Lot TJ2655242); https://www.thermofisher.com/antibody/product/Phospho-CaMKII-beta-gamma-delta-Thr287-Antibody-Polyclonal/PA5-37833; validated by cell treatment with ionomycin, which increased the specific signal; absent staining in the presence of blocking peptide.

rabbit monoclonal anti-phospho-Thr286/287 CAMK2 (alpha, beta, gamma, delta) antibody (D21E4; 12716; CST; Lot 5); https://www.cellsignal.com/products/primary-antibodies/phospho-camkii-thr286-d21e4-rabbit-mab/12716; validated with cell extracts of [illegible]-phosphatase-treated cells vs. untreated cells.

rabbit polyclonal anti-phospho-Thr305 CAMK2 (alpha, beta, gamma, delta) antibodies (Thr307 in mouse CAMK2D) (Abnova PAB29254; B1SA01040G00470; Lot 8630); https://www.abnova.com/products/products_detail.asp?catalog_id=PAB29254; validated in western blot with 3T3 cells, which were treated without and with antigen-specific peptide.

rabbit monoclonal anti-Desmin antibody [Y66] (ab32362, abcam; Lot GR152193-45); https://www.abcam.com/desmin-antibody-y66-cytoskeleton-marker-ab32362.html.

mouse monoclonal anti-ATP5A antibody [15H4C4] (ab14748; abcam; Lot GR3238328-3; lot GR3306993-21; Lot GR3306993-10); https://www.abcam.com/atp5a-antibody-15h4c4-mitochondrial-marker-ab14748.html.

rabbit monoclonal anti-MLKL antibody (D6W1K) (mouse specific; 37705: CST; Lot 4); https://www.cellsignal.com/products/primary-antibodies/mlkl-d6w1k-rabbit-mab-mouse-specific/37705;

rabbit monoclonal anti-phospho-S345-MLKL antibody [EPR9515(2)] (ab 196436; abcam; Lot GR3334380-2, Lot GR3334380-4); https://www.abcam.com/mlkl-phospho-s345-antibody-epr95152-ab196436.html; validated by dot blot analysis with phosphorylated peptide vs. unphosphorylated peptide, which gave no signal, and by cell treatment with TNFalpha, SMac mimetic and z-VAD vs. untreated cells, which gave no signal.

rat monoclonal anti-MLKL antibody [3H1] (ab243142; abcam; Lot GR3357550-4); https://www.abcam.com/mlkl-antibody-3h1-ab243142.html.

mouse monoclonal anti-phospho-S345 MLKL antibody (MABC1158, Clone 7C6.1; Lot 3471964; EMD Millipore Corporation); https://www.merckmillipore.com/CH/de/product/Anti-phospho-MLKL-Ser345-Antibody-clone-7C6.1,MM_NF-MABC1158; validated by cell treatment with TNF+zVAD.

rabbit monoclonal anti-phospho-S358-MLKL (D6H3V) (mAb No. 9168; Lot 4; CST); https://www.cellsignal.com/products/primary-antibodies/phospho-mlkl-ser358-d6h3v-rabbit-mab/91689.

rabbit monoclonal anti-MLKL (D2I6N) (mAb No. 14993; Lot 3; CST) https://www.cellsignal.com/products/primary-antibodies/mlkl-d2i6n-rabbit-mab/14993.

rabbit polyclonal anti-RYR2 antibodies (Invitrogen PA5-77717; Lot XI3697745; ThermoFisher Scientific); https://www.thermofisher.com/antibody/product/RyR2-Antibody-Polyclonal/PA5-77717.

rabbit polyclonal anti-phospho-Ser2814 RYR2 antibodies (CABP0624; Lot 3516978205; AssayGenie); https://www.assaygenie.com/phospho-ryr2-s2814-rabbit-polyclonal-antibody-cabp0624/.

mouse monoclonal anti-alpha-Tubulin antibody, clone DM1A (T6199; Sigma; Lot 029M4842V); https://www.sigmaaldrich.com/CH/de/product/sigma/t6199.

peroxidase-conjugated AffiniPure F(ab')2 Fragment Goat Anti-Mouse IgG Fc gamma Fragment Specific (minimal crossreaction to human, bovine and horse serum proteins) (115-036-071, Jackson ImmunoResearch Laboratories Inc., West Grove, USA; lot 151768; lot 151642; lot 148365; lot 136859); https://www.jacksonimmuno.com/catalog/products/115-036-071.

peroxidase-conjugated AffiniPure F(ab')2 Fragment Goat Anti-Rabbit IgG, Fc Fragment-Specific (minimal cross-reaction to human serum proteins) (111-036-046; Jackson ImmunoResearch Laboratories Inc., West Grove, USA; lot 144587; lot 149886; lot 150552); https://www.jacksonimmuno.com/catalog/products/111-036-046.

protein A, Peroxidase Conjugate (539253-1MG; EMD Millipore Corp. USA ; Lot 3192062 ; Lot K3595168); https://www.merckmillipore.com/CH/de/product/Protein-A-Peroxidase-Conjugate,EMD_BIO-539253.

goat anti-Rabbit IgG (H+L) Highly Cross-Adsorbed Secondary Antibody, Alexa Fluor 488 (A11034; Invitrogen by ThermoFisher Scientific; lot 1937195); https://www.thermofisher.com/antibody/product/Goat-anti-Rabbit-IgG-H-L-Highly-Cross-Adsorbed-Secondary-Antibody-Polyclonal/A-11034.

goat anti-Mouse IgG (H+L) Cross-Adsorbed Secondary Antibody, Alexa Fluor 568 (A11004; Invitrogen by ThermoFisher Scientific; lot 1906485); https://www.thermofisher.com/antibody/product/Goat-anti-Mouse-IgG-H-L-Cross-Adsorbed-Secondary-Antibody-Polyclonal/A-11004.

## Animals and other research organisms

Policy information about studies involving animals; ARRIVE guidelines recommended for reporting animal research, and Sex and Gender in Research

| | |
|---|---|
| Laboratory animals | Mice were on a FVB/N background and C57Bl/6N (B6) background, as indicated. All the mice were housed in groups of 2-4 in individually ventilated cages under specified pathogen-free conditions with controlled ambient temperature (18-23 °C) and humidity levels (40-50 %), a 12 h light-dark cycle, and ad libitum access to food and water. No randomization was performed in animal experiments. The study used male and female mice, and investigated the phenotype of transgenic mice in comparison to age- and sex-matched control mice, i.e., non-transgenic FVB mice and B6 mice as indicated at an age of 3-4 months and 8 months. |
| Wild animals | The study did not involve wild animals. |
| Reporting on sex | The study used male and female mice, and investigated the phenotype of male and female transgenic mice in comparison to age- and sex-matched control mice, as indicated. |
| Field-collected samples | The study did not involve samples from the field. |
| Ethics oversight | The animal research study complies with all ethical regulations. All animal experiments were approved by the local committees on animal research (Kantonales Veterinäramt Zurich ZH215/2020, date of approval 15.03.2021; Kantonales Veterinäramt Zurich 145-G, approval 14.02.2013; Kantonales Veterinäramt Zürich 126/2009, date of approval 04.08.2009; Medical Research Center (MRC), Cairo, date of approval 02.01.2007). |

Note that full information on the approval of the study protocol must also be provided in the manuscript.
