# Supplementary figures and images for: BBLN triggers CAMK2D pathology in mice under cardiac pressure overload and potentially in unrepaired hearts with tetralogy of Fallot
Source: Nat Cardiovasc Res. 2023 Oct 26;2(11):1044–59. doi: 10.1038/s44161-023-00351-6 (PMC11041739; doi:10.1038/s44161-023-00351-6)

**Figure 1b**

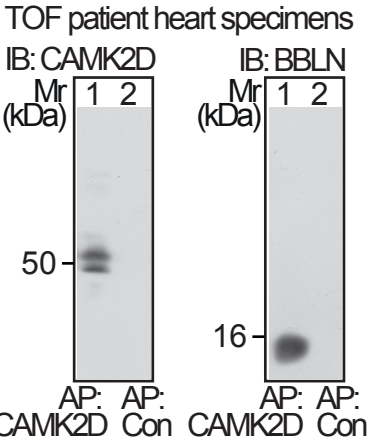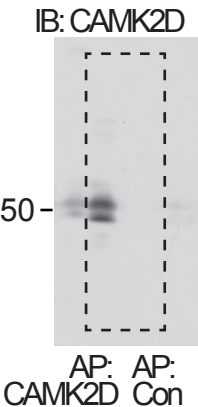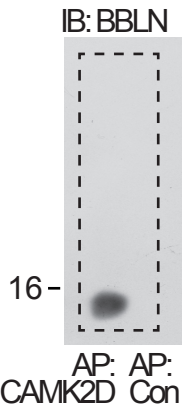

Supplement: Supplementary file 6 — Unprocessed western blots. [file 44161_2023_351_MOESM6_ESM.pdf]

**Figure 2c**

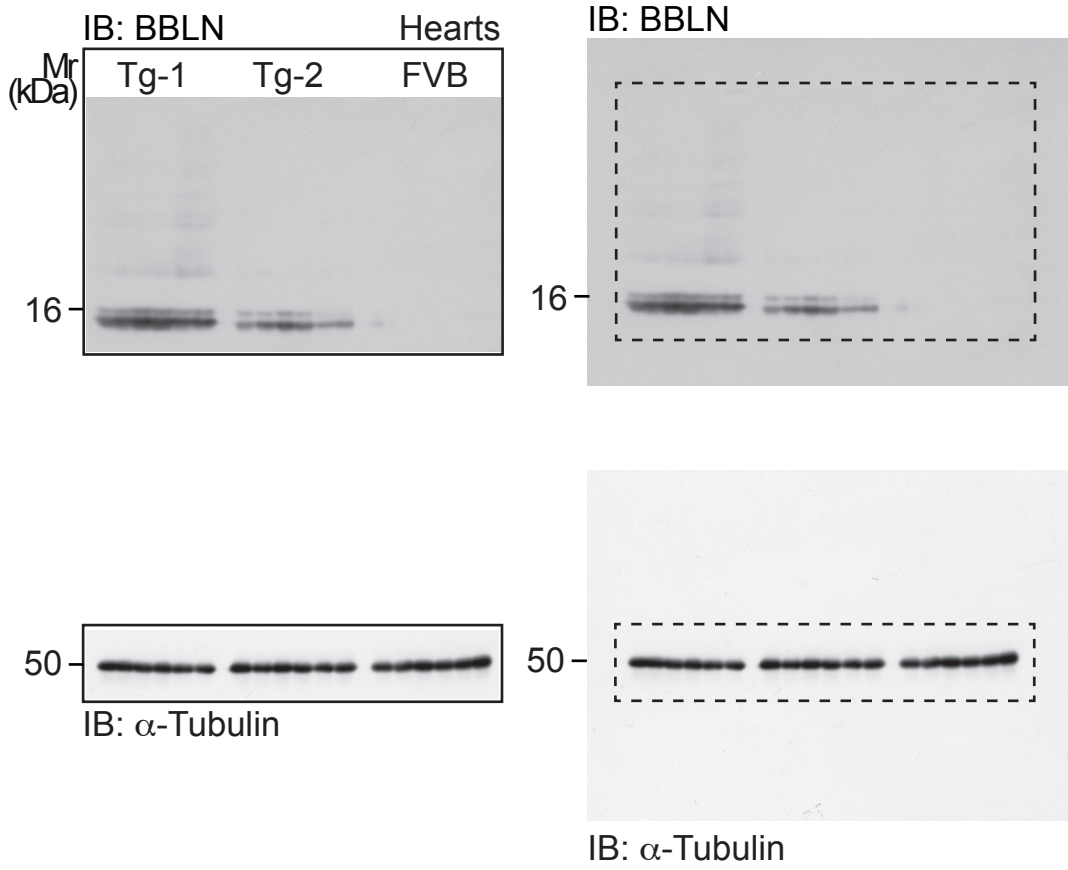

Supplement: Supplementary file 8 — Unprocessed western blots. [file 44161_2023_351_MOESM8_ESM.pdf]

**Figure 4b**

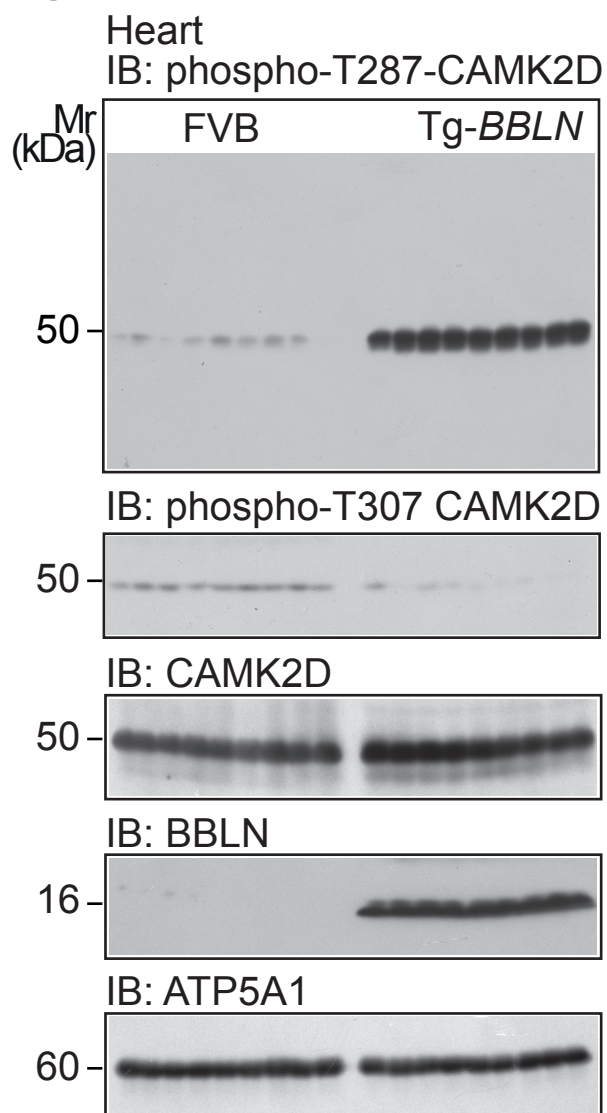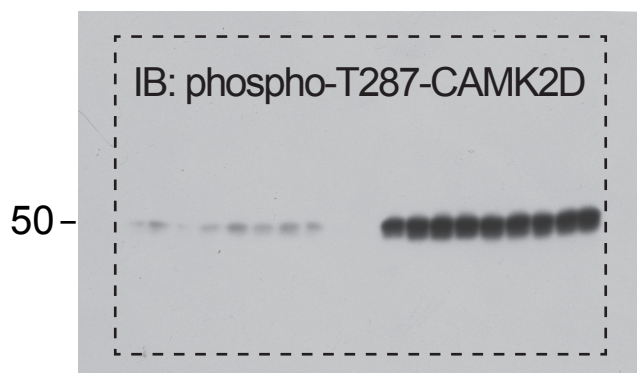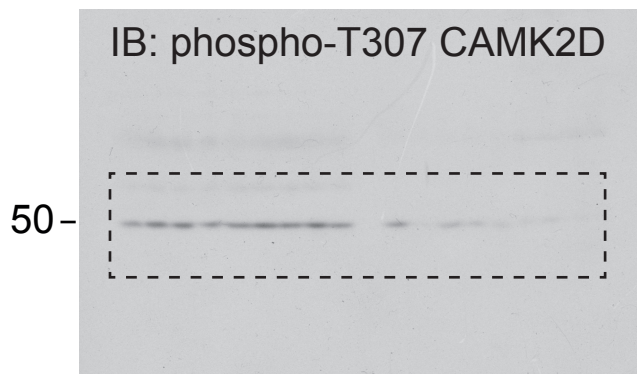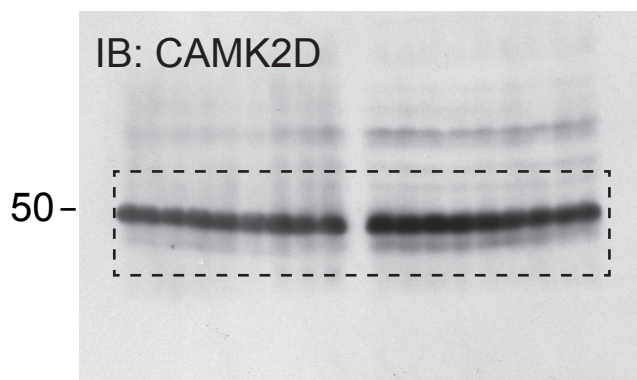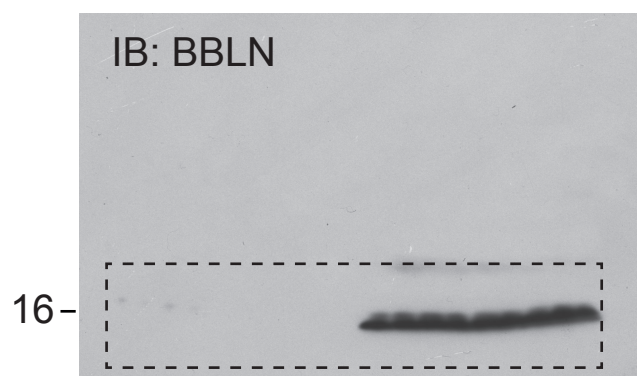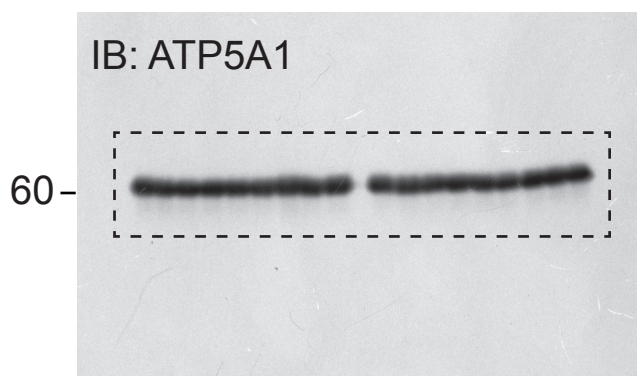

Figure 4c

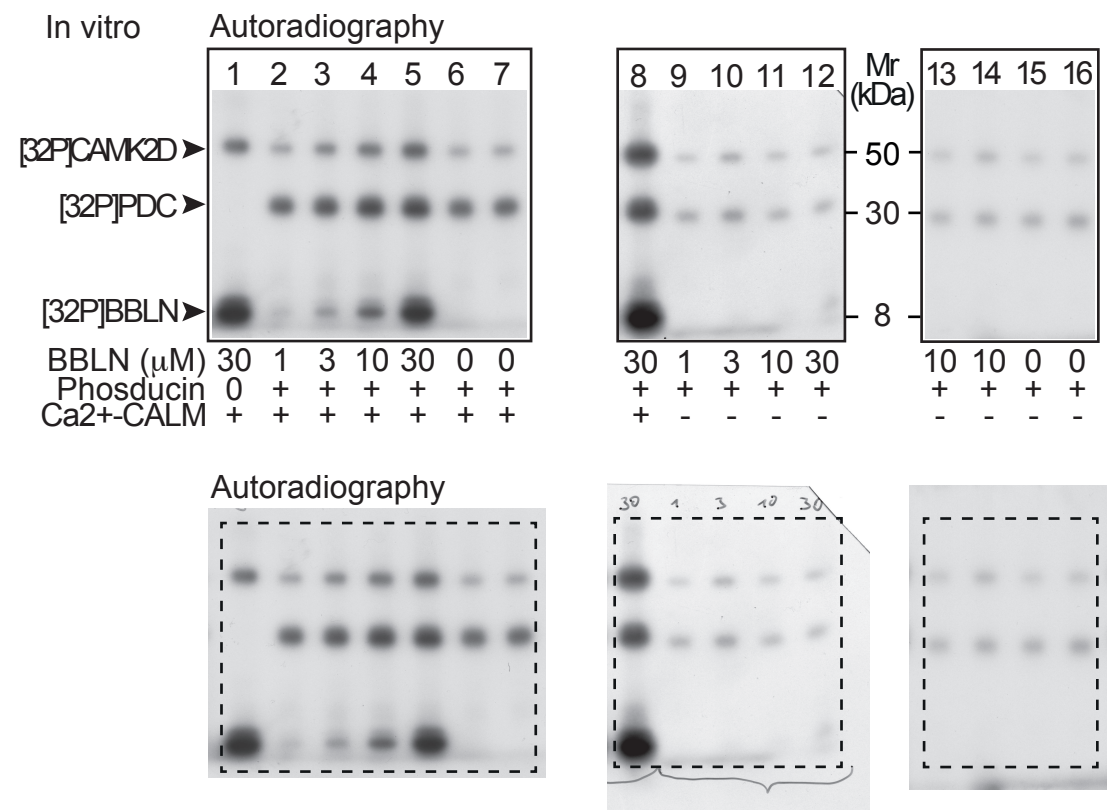

**Figure 4e**

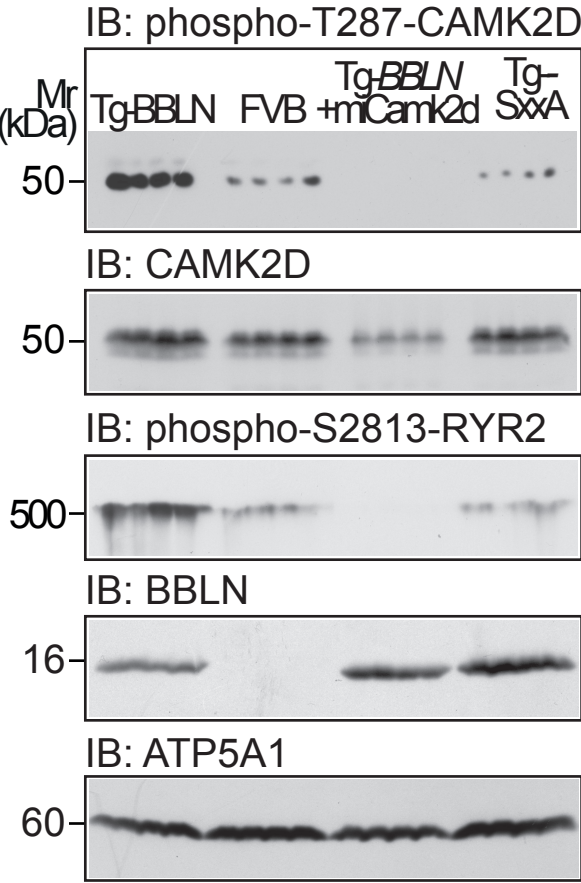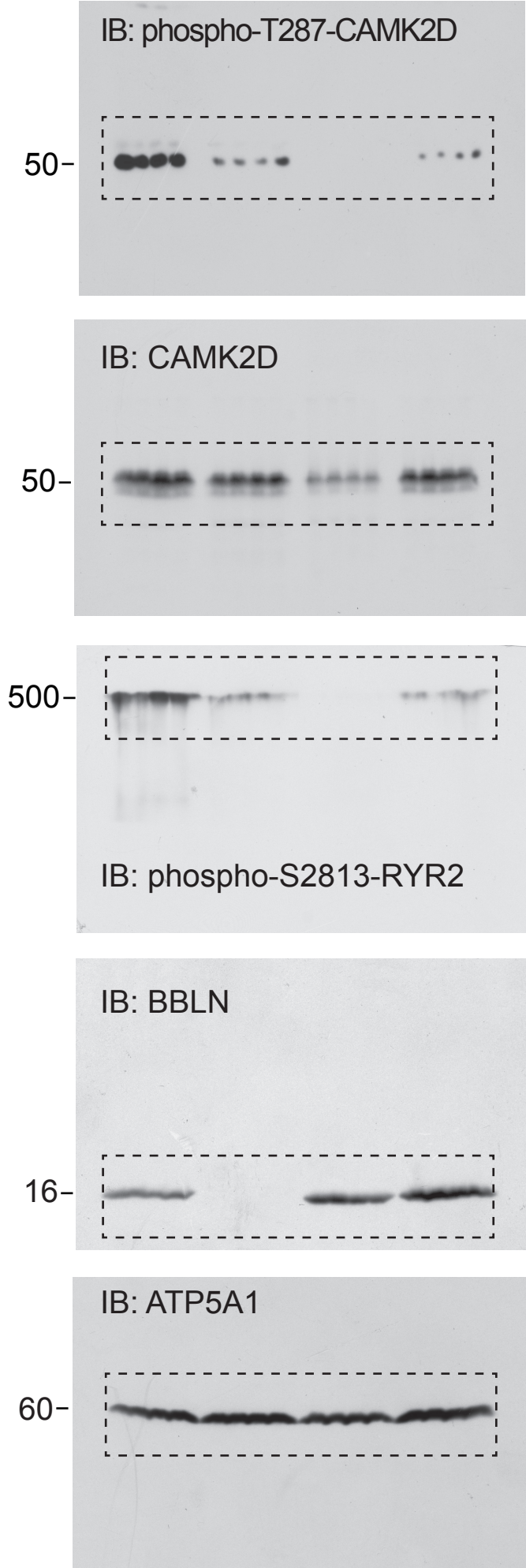

Supplement: Supplementary file 11 — Unprocessed western blots. [file 44161_2023_351_MOESM11_ESM.pdf]

**Figure 6a**

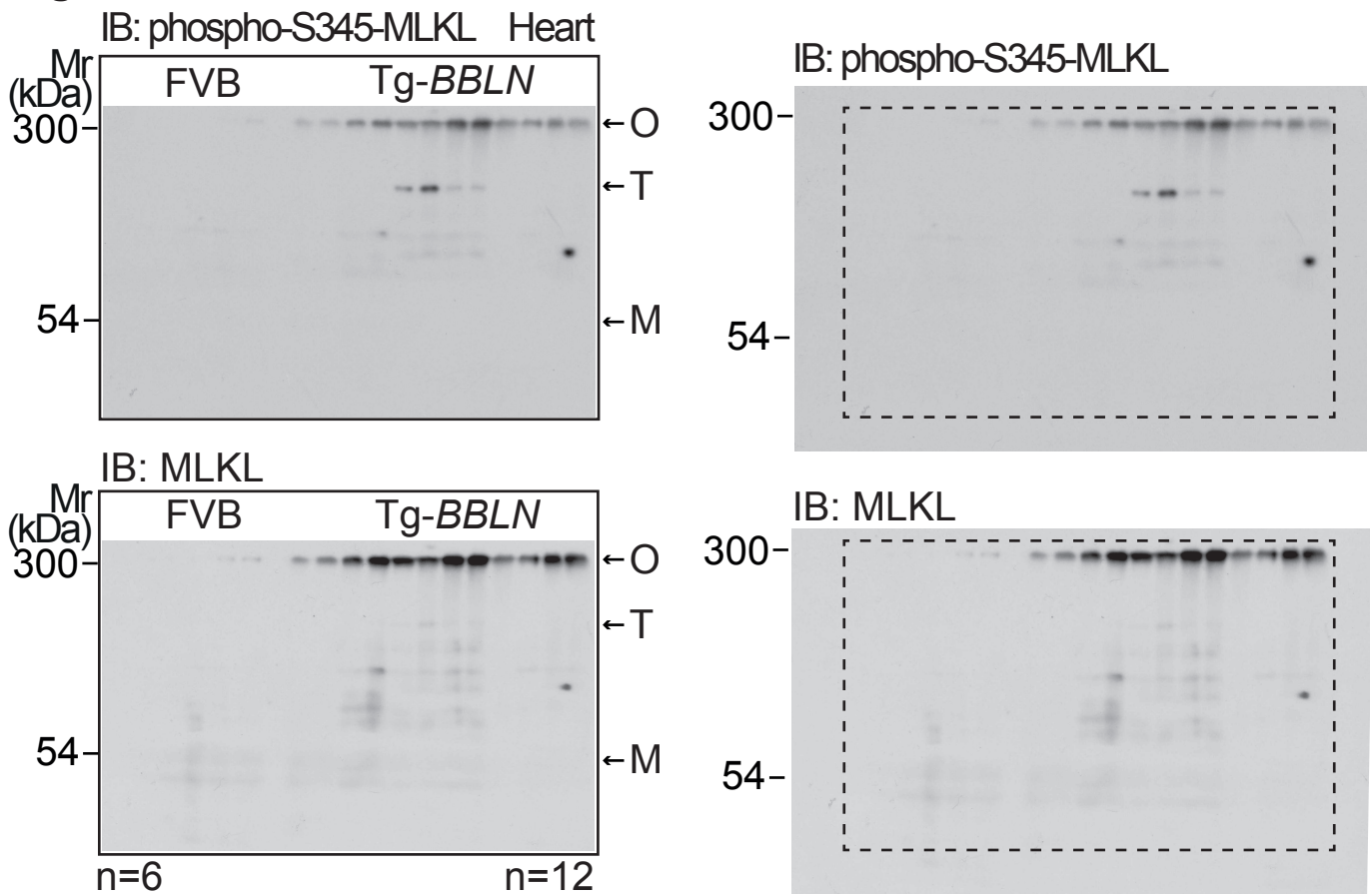

**Figure 6b**

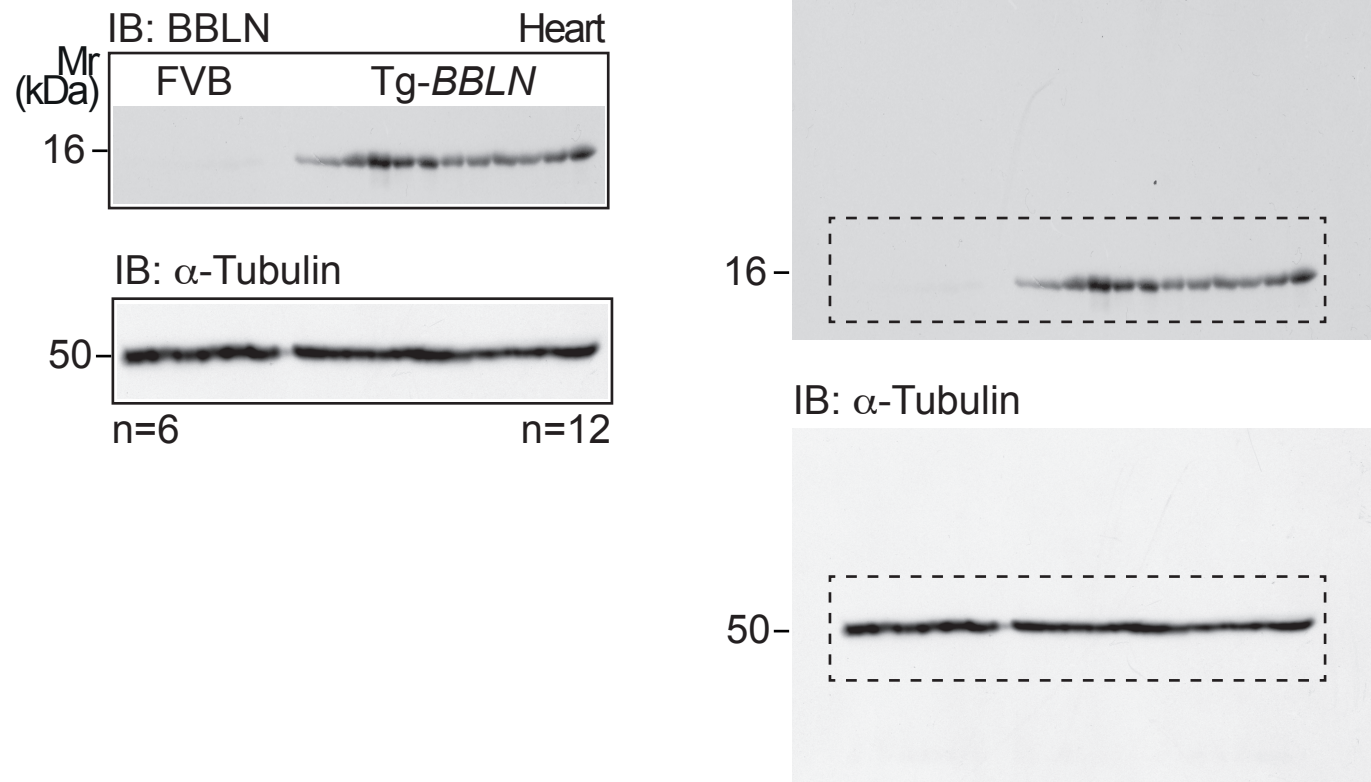

**Figure 6f**

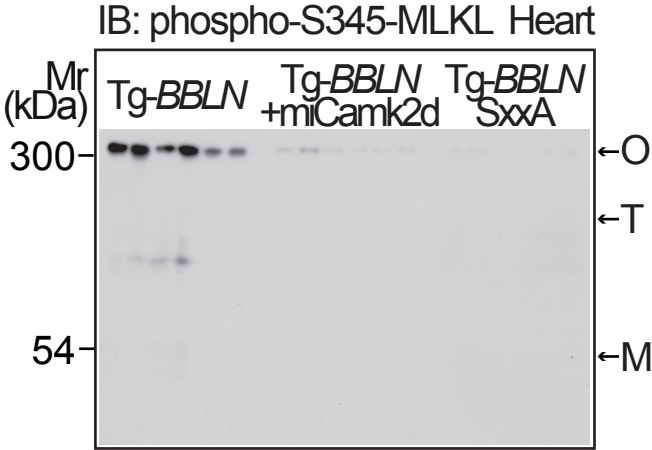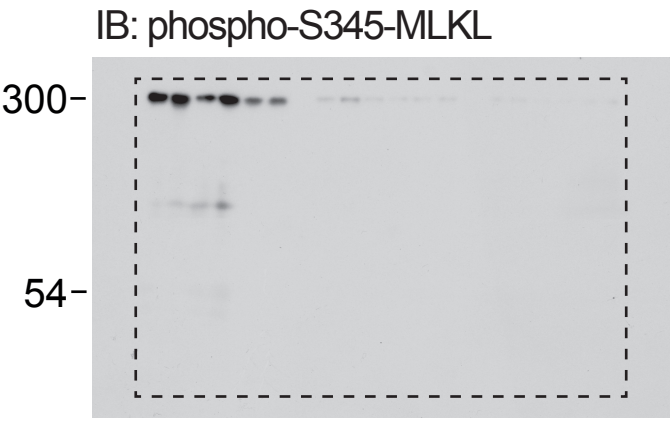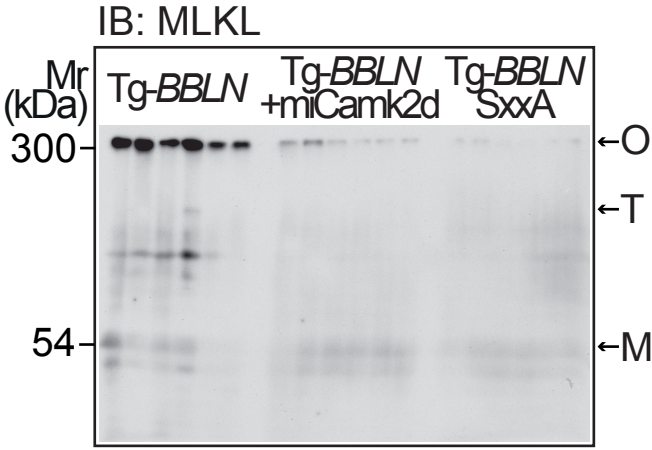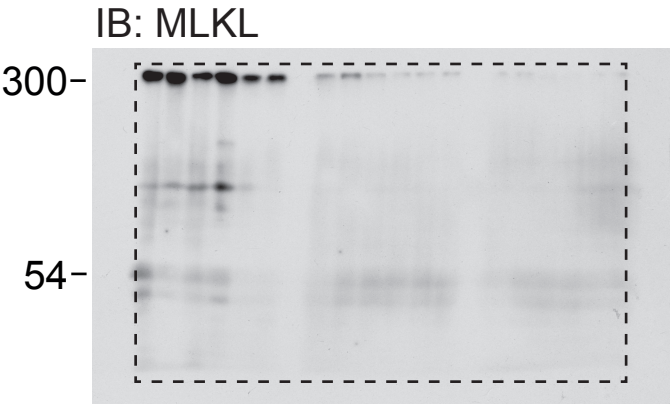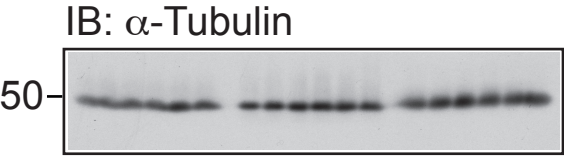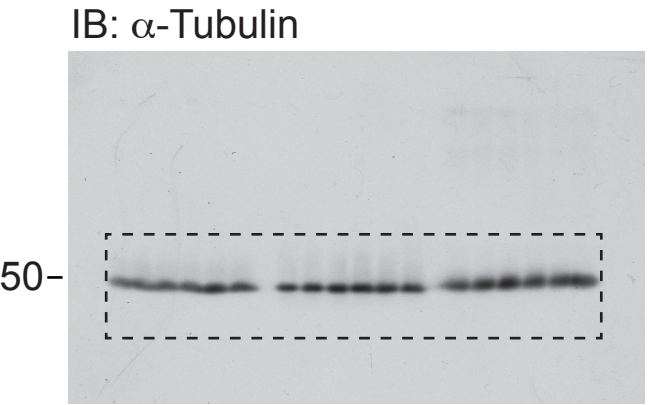

Supplement: Supplementary file 14 — Unprocessed western blots. [file 44161_2023_351_MOESM14_ESM.pdf]

**Figure 7a,b**

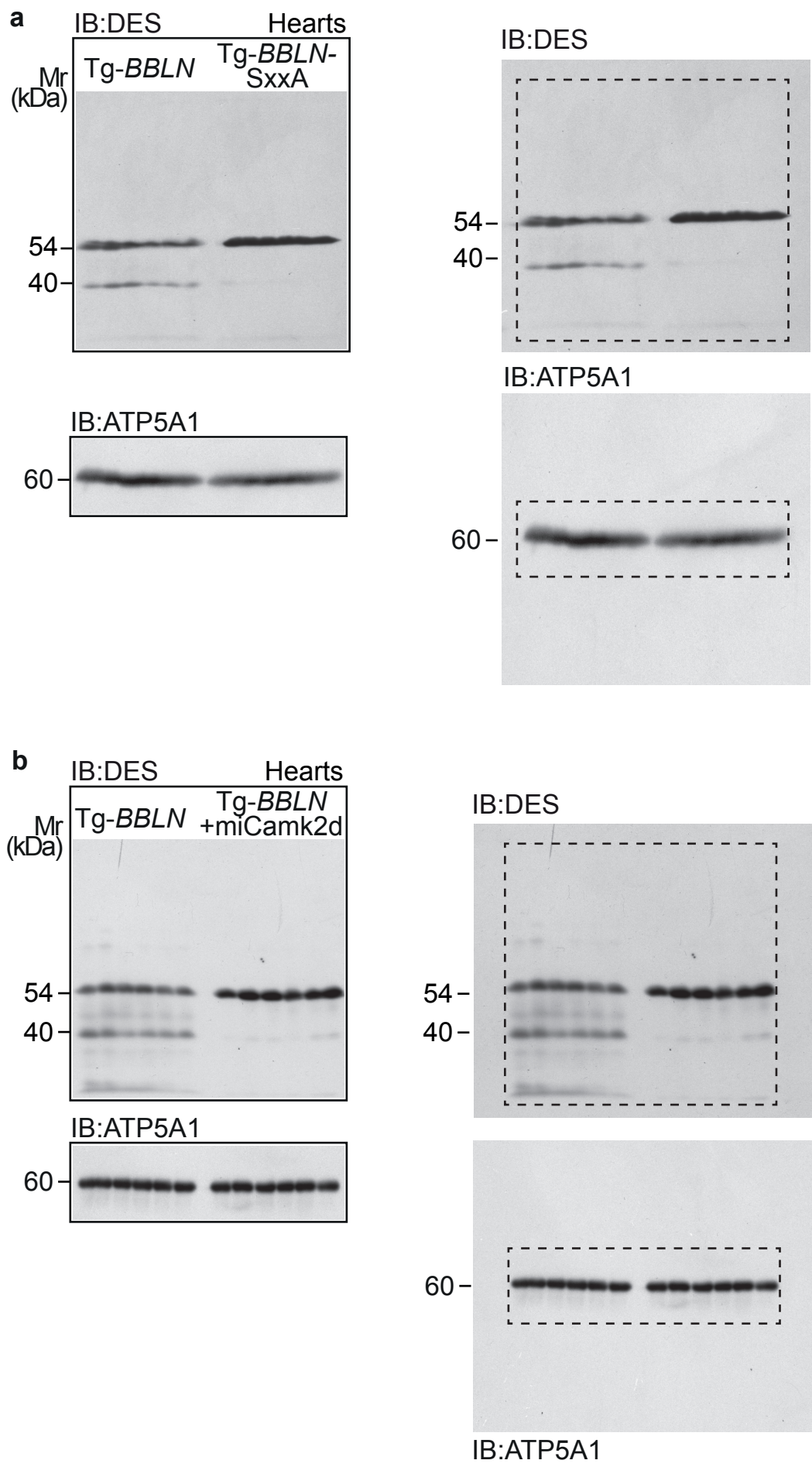

Supplement: Supplementary file 16 — Unprocessed western blots. [file 44161_2023_351_MOESM16_ESM.pdf]

Figure 8a

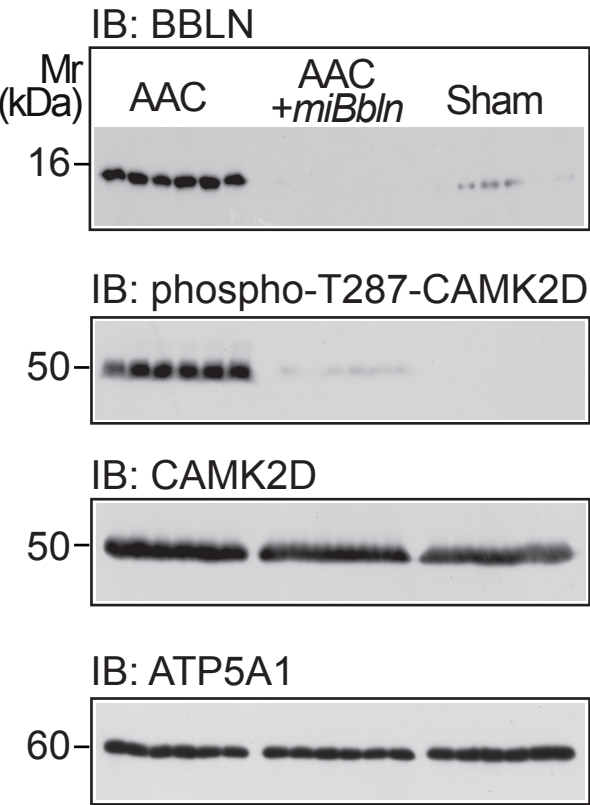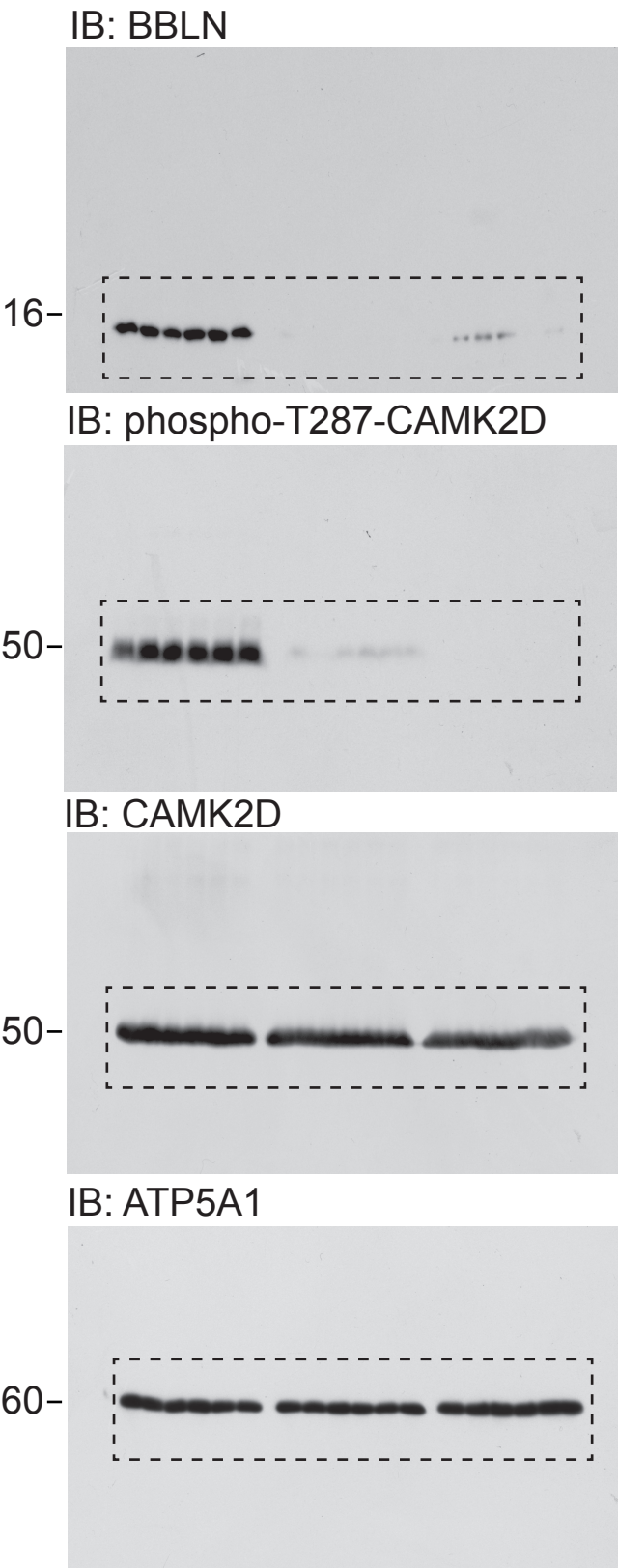

**Figure 8b**

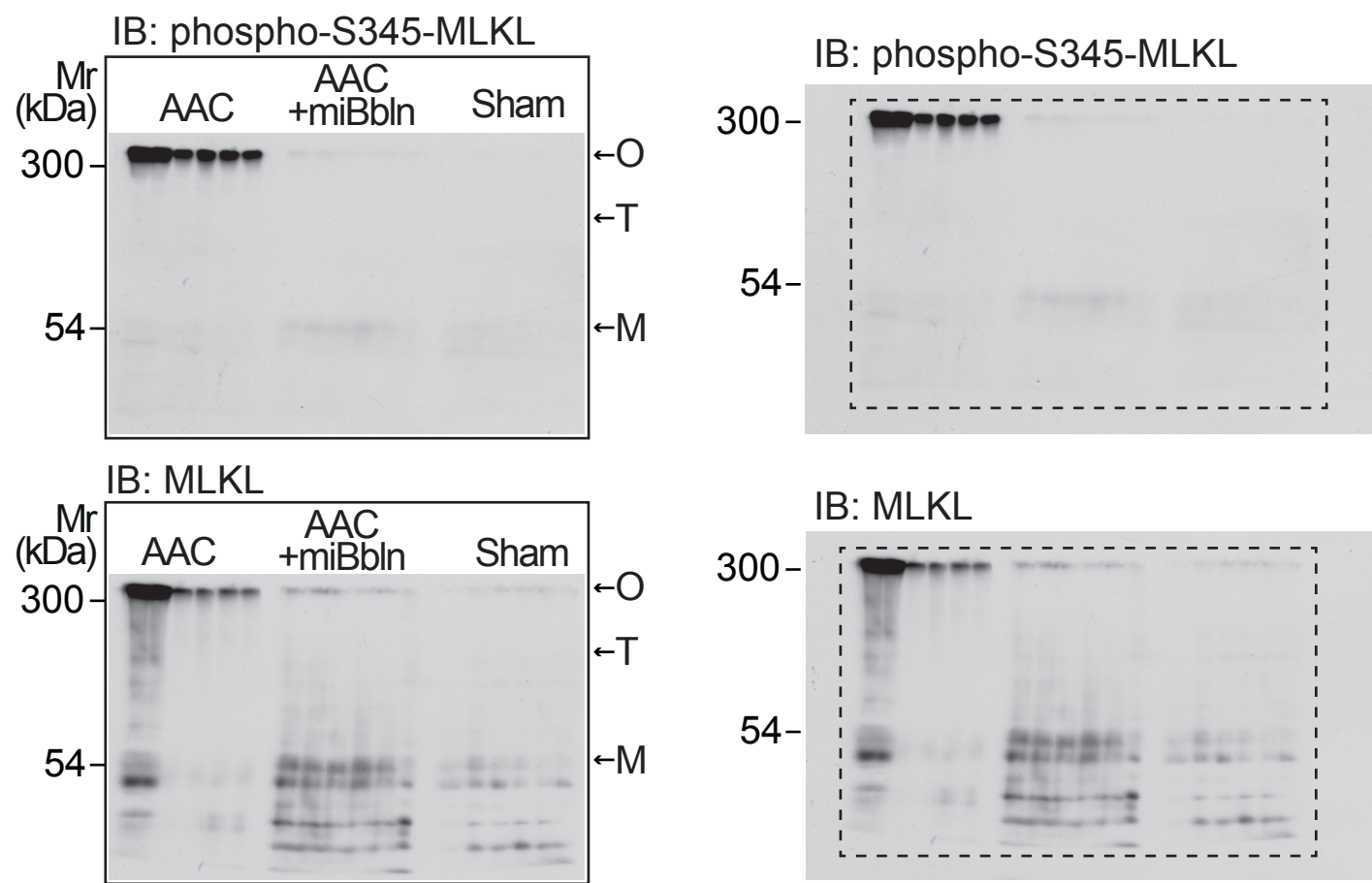

Supplement: Supplementary file 18 — Unprocessed western blots. [file 44161_2023_351_MOESM18_ESM.pdf]

Extended Data Fig. 6a,b

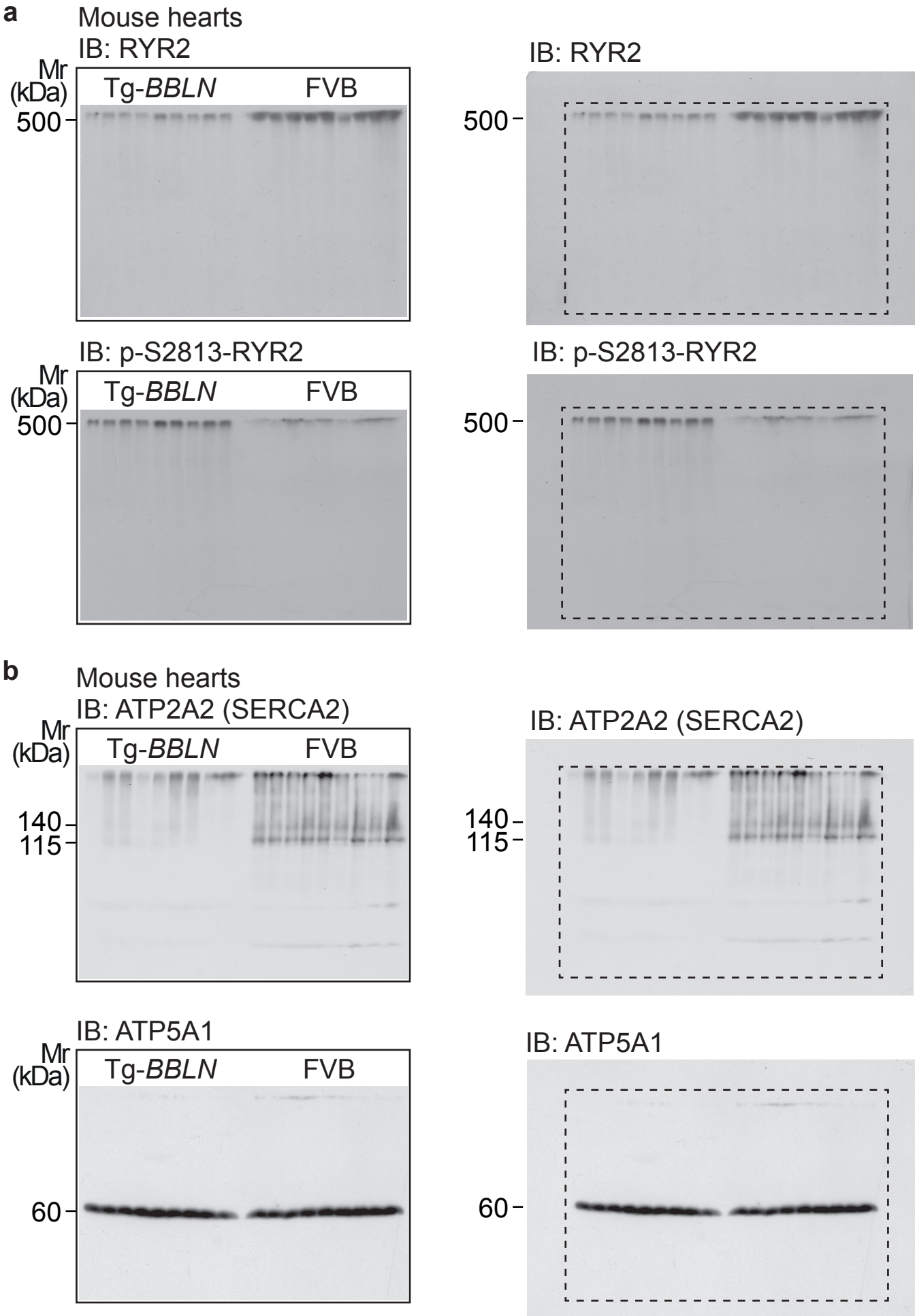

Supplement: Supplementary file 25 — Unprocessed western blots. [file 44161_2023_351_MOESM25_ESM.pdf]

**Extended Data Fig. 7a**

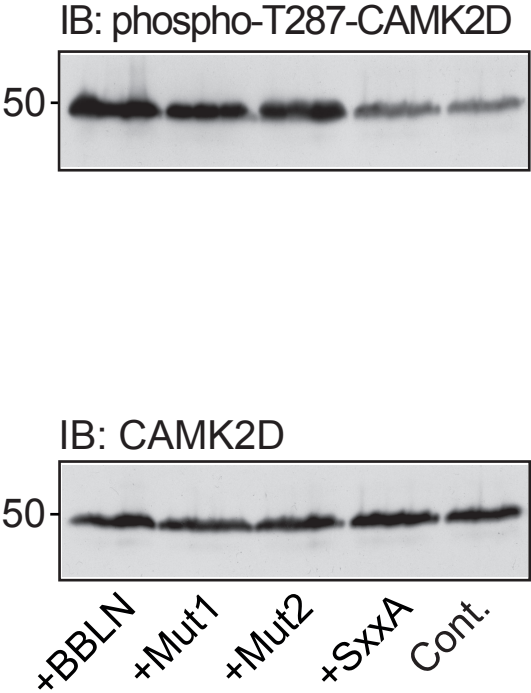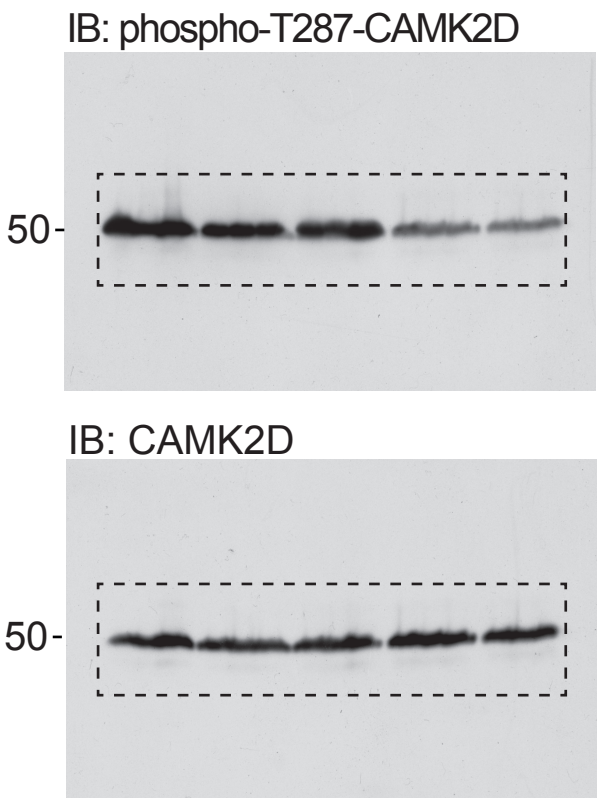

**Extended Data Fig. 7c**

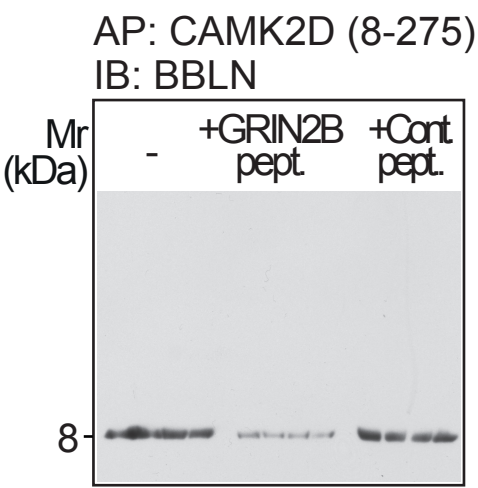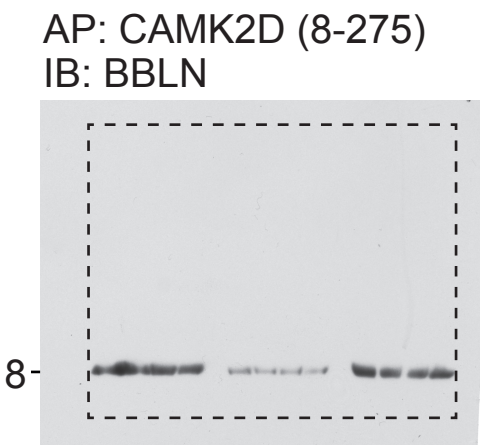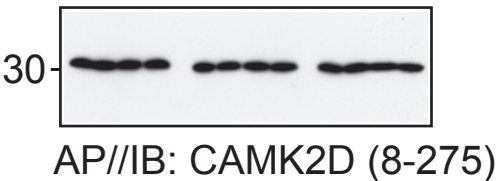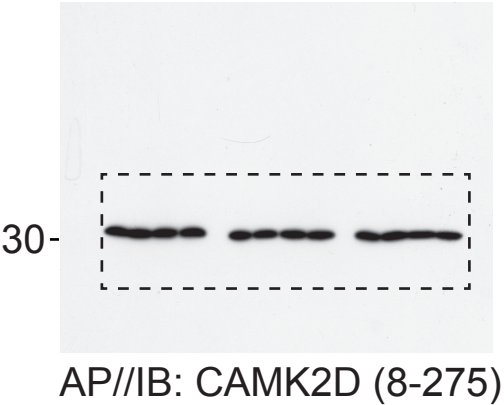

## Extended Data Fig. 7d

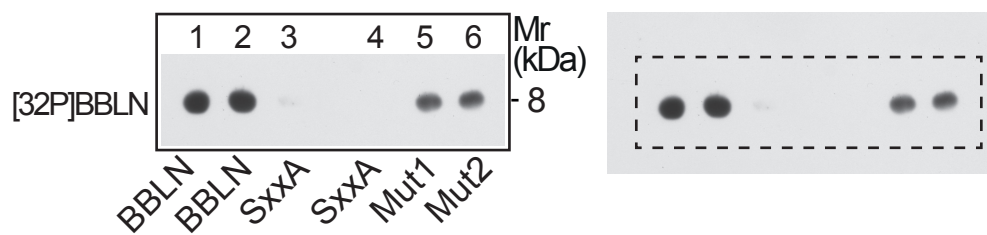

## Extended Data Fig. 7e

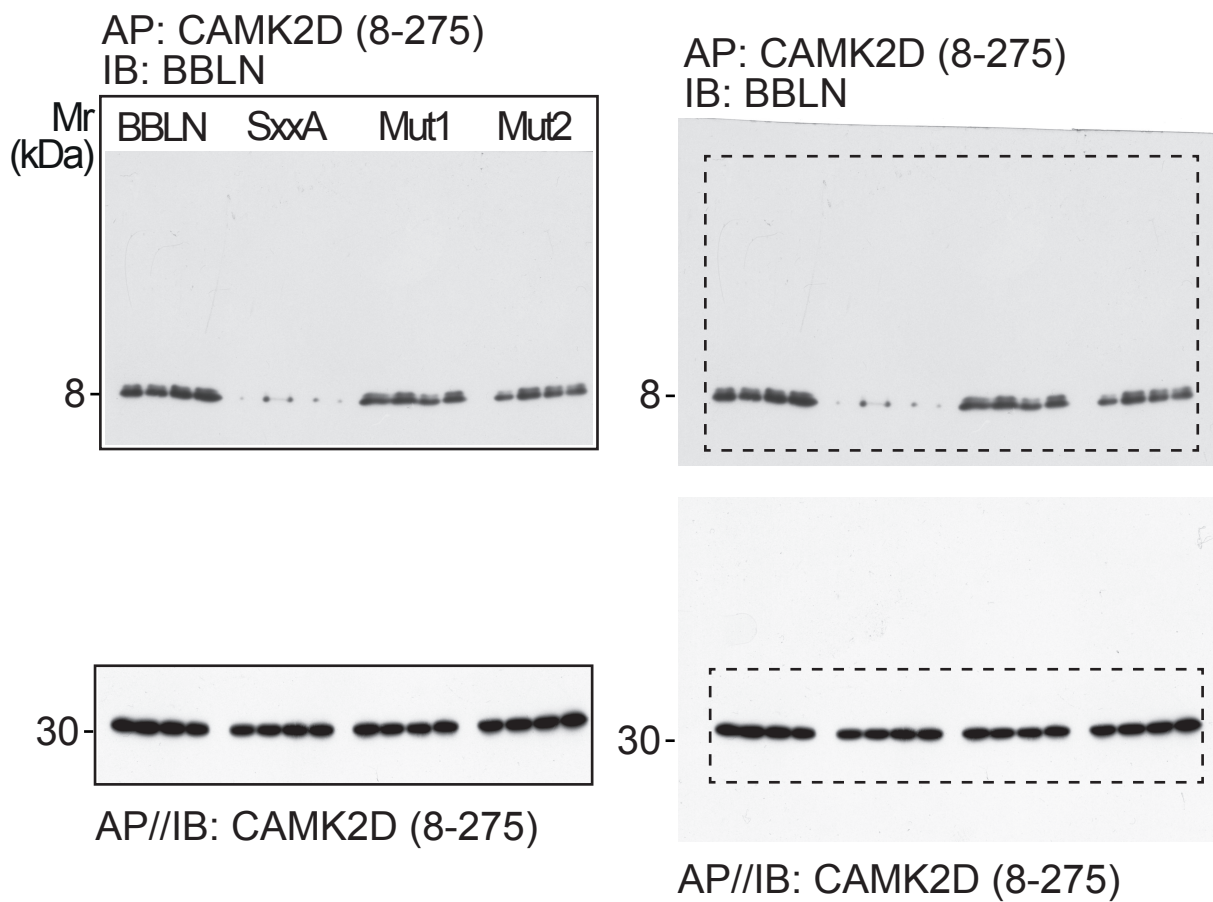

Supplement: Supplementary file 27 — Unprocessed western blots. [file 44161_2023_351_MOESM27_ESM.pdf]

Extended Data Figure 9a

TOF patient heart specimens

**a**

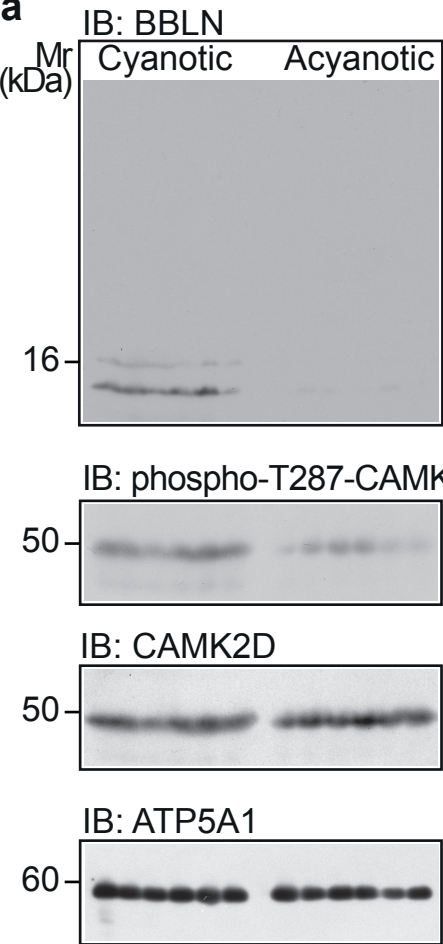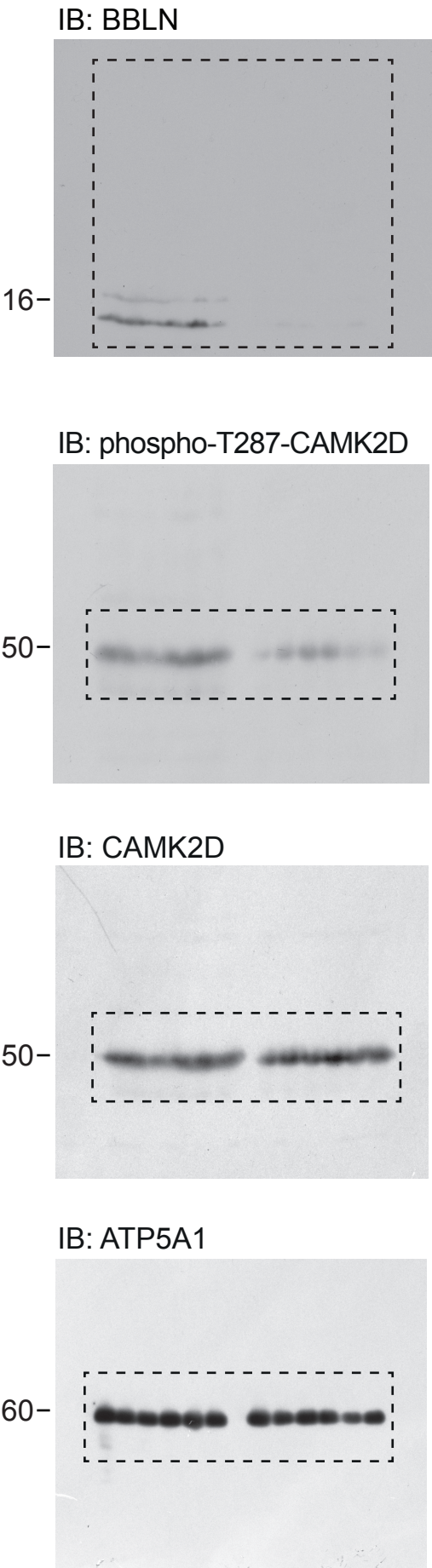

## Extended Data Figure 9b

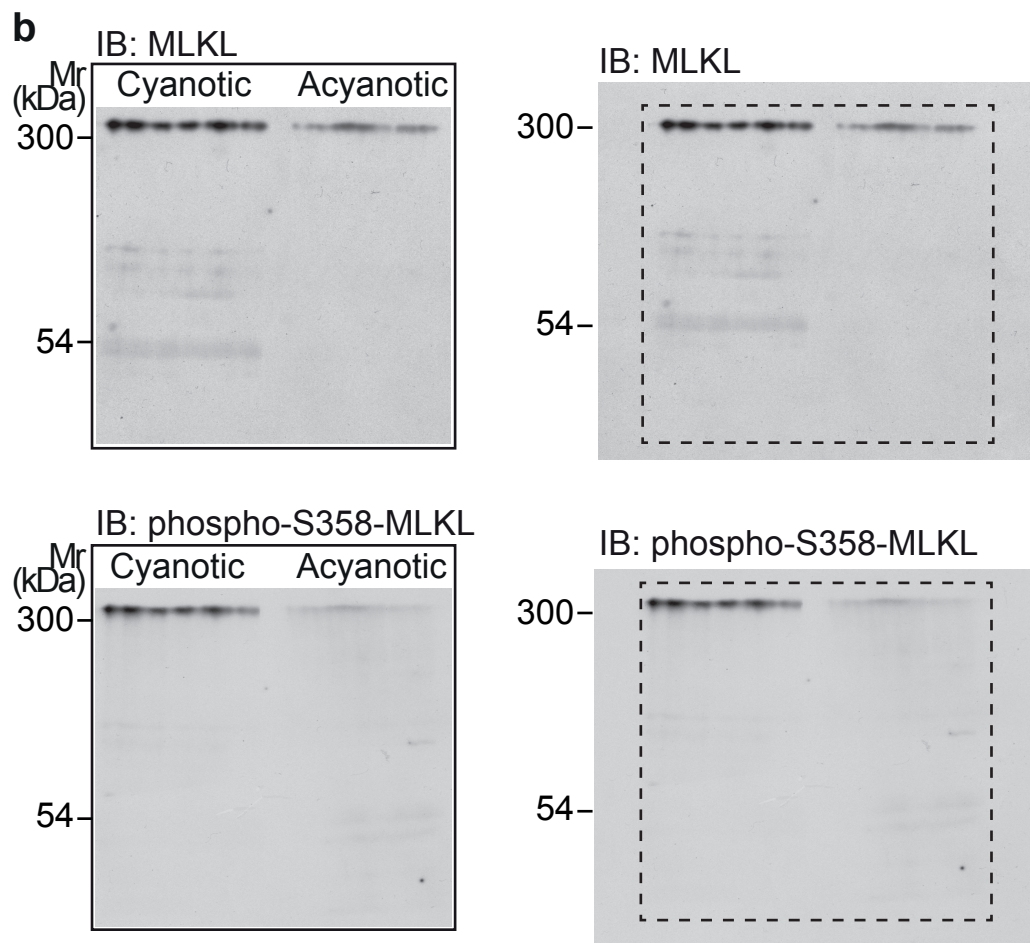

Supplement: Supplementary file 29 — Unprocessed western blots. [file 44161_2023_351_MOESM29_ESM.pdf]

### Extended Data Fig. 10b

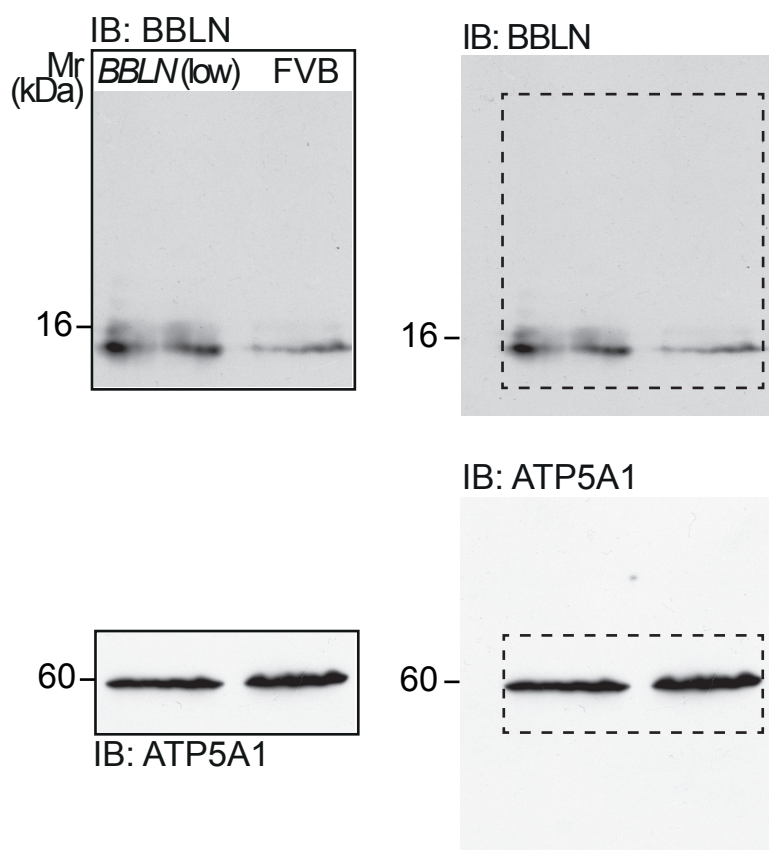

Supplement: Supplementary file 31 — Unprocessed western blots. [file 44161_2023_351_MOESM31_ESM.pdf]
